# Supplementary material for: Nutrient asymmetry challenges the sustainability of Ukrainian agriculture
Source: Commun Earth Environ. 2025 Nov 4;6(1):845. doi: 10.1038/s43247-025-02826-9 (PMC12588375; doi:10.1038/s43247-025-02826-9)
Supplement: Supplementary file 2 — Supplementary Information [file 43247_2025_2826_MOESM2_ESM.pdf]

Supplementary Information

for

Nutrient asymmetry challenges the sustainability of Ukrainian agriculture

Sergiy Medinets[1,2*†], Oene Oenema[3], Bryan M. Spears[1], Andriy Buyanovskiy[4], Volodymyr Medinets[2], William J. Brownlie[1], Eiko Nemitz[1], Massimo Vieno[1] and Mark A. Sutton[1*‡]

[1] UK Centre for Ecology and Hydrology, Edinburgh, UK
[2] Regional Centre for Integrated Environmental Monitoring, Odesa National I. I. Mechnikov University, Odesa, Ukraine
[3] Wageningen University & Research, Wageningen, The Netherlands
[4] Department of Geography of Ukraine, Soil Science and Land Cadastre, Faculty of Geology and Geography, Odesa National I. I. Mechnikov University, Odesa, Ukraine

[*]Corresponding authors
[†] sermed@ceh.ac.uk
[‡] ms@ceh.ac.uk

Table of Contents

Note 1: Supplementary Introduction **2**

Note 1.1: Ukraine exports of wheat, maize and sunflower 2

Note 2: Supplementary Results and Discussion **2**

Note 2.1: Mean N, P and K inputs with fertilizers and the outputs with crop yields 2

Manure application deficits. *3*

Note 2.2: Soil degradation and the current state of soils in Ukraine 3

Soil organic matter (SOM) as key indicator of soil quality. *3*

Soil degradation and SOM loss. *4*

Concentrations of plant-available nitrates, phosphates and potassium in Ukrainian soils. *5*

Technical details on the long-term agrochemical monitoring in Ukraine. *6*

Note 2.3: High N-mineralization rate in black soils (Chernozems) 7

Note 2.4: Mean N, P and K balances at country scale 7

Note 2.5: Mean N, P and K balances at county scales 7

Note 2.6: Mean N, P and K use efficiencies at country scale 7

Note 2.7: Mean N, P and K use efficiencies at county scales 8

Note 2.8: Detailed description of nutrient management scenarios 8

Scenario design and baseline calibration *8*

Supporting data and calculations *12*

Assumptions and limitations *13*

Note 2.9: Smart Fertilizer Planner 14

Principle and usage. *14*

Limitations and assumptions. *15*

Prerequisites and requirements. *15*

Further development. *15*

Other tools available. *15*

Note 2.10: Farming during wartime 16

Agricultural damages. *16*

Challenges. *16*

Positive shifts amid the war. *16*

Support programs for small farmers. *17*

New Agricultural Policy. *17*

Note 3: Supplementary Methods **18**

Note 3.1: Accounting for the data availability from the temporarily occupied areas of Ukraine 18

Note 3.2: Accounting for the total atmospheric deposition of N, P and K, including organic constituents 18

Note 3.3: Accounting for crop residue management 19

Note 3.4: Accounting for crop rotation practice 19

Note 3.5: Limitations of the partial nutrient budget approach recommended by EUNEP 19

Supplementary Tables **21**

Supplementary Figures **27**

Supplementary References **51**

Note 1: Supplementary Introduction

Note 1.1: Ukraine exports of wheat, maize and sunflower

In 2021/2022, Ukraine was the world's ninth-largest wheat producer and the fifth-largest exporter (**Supplementary Fig. 2**). In 2021, Ukraine exported 20 million metric tons of wheat grain, valued at $5.1 billion (USDA, 2022, 2024). The two primary markets were: (i) the Middle East and North Africa (MENA), notably Egypt and Türkiye, which together accounted for 50% of exports, and (ii) East and South Asia, largely Indonesia, Pakistan and Bangladesh, which took 32% (**Supplementary Fig. 3**; USDA, 2022; Glauber, 2024). Wheat is grown in all regions of Ukraine.

For maize, Ukraine was the eighth-largest producer globally and the fourth-largest exporter in 2021/2022 (**Supplementary Fig. 4**). Maize grain exports totaled 25 million metric tons, worth $5.9 billion (USDA, 2022, 2024). The largest destinations were East Asia, mainly China (35%) and the European Union (33%), with other key buyers from MENA, including Egypt, Iran and Türkiye (25%) (**Supplementary Fig. 3**; USDA, 2022; Glauber, 2024). Maize production is largely concentrated in central and northern Ukraine.

Ukraine has historically been the top global producer and exporter of sunflower meal, oil and seed. In 2021, sunflower oil exports amounted to $6.4 billion, making up 75% of Ukraine's total oil and fat product exports (USDA, 2022). Major importers included the European Union (32%), India (31%) and China (15%) (USDA, 2022). Sunflower meal export (value $1.3 billion) was primarily to China (48%), the EU (25%) and Türkiye (7%). Production is concentrated in eastern and southeastern Ukraine (USDA, 2022).

Note 2: Supplementary Results and Discussion

Note 2.1: Mean N, P and K inputs with fertilizers and the outputs with crop yields

We observed a gradual increase in the application of N per hectare of utilized agricultural area (UAA) for all three crops since early 2000s, with a slight boost from 2016 up to its maximum in 2020-2021, the last pre-war years (**Fig. 1**). In fact, a slow recovery of Ukrainian agriculture had started earlier since the implementation of reforms in 1997 after a long recession period: gradual stagnation during 1987-1991, the last years of the Soviet era, followed by a deep decline due to disruption of supply and marketing chains following independence on 24th August 1991 (Lerman, 2008).

Use of N fertilizer steadily increased during 2000-2021 and was associated with an increased N withdrawal with harvested yield; mean N yields of maize, wheat and sunflower over this period increased 2.6, 2.3 and 2.0 times at country level, respectively (**Fig. 1**).

Use of P and K fertilizers (sum or synthetic and organic) was low between 1990 and 2000, but thereafter increased gradually up to 2021. Overall, from 2000 to 2021, P and K fertilizer applications increased by 3.2 and 1.8 times for wheat, respectively, and by 5.8 and 4.22 times for maize, respectively (**Fig. 1**). Use of both P and K fertilizer increased significantly by 18.1 and 15.9 times for sunflower, respectively, between 2000 to 2021 (**Fig. 1**).

The relatively slow recovery rate of P and K fertilizer use compared to N fertilizer in Ukrainian agriculture (**Supplementary Fig. 7-8**) might be linked to the limited domestic production capacity of P and K fertilizers, most of which imported and costly, as well as the dramatic decrease in manure application (see 'Manure application deficits' below). According to FAOSTAT (**Supplementary Fig. 9;** FAO, 2024), Ukraine's own synthetic fertilizer production could meet 50-61% of its agricultural demand for N, but only 0-10% for P and none for K over 2019-2021. Hence, most synthetic P and K fertilizers had to be imported. We do not have access to detailed data on synthetic N, P and K fertilizer production in Ukraine, as such information is not published in national statistics due to the confidentiality requirements under the Law of Ukraine on the State Statistics (SSSU, 2025). However, publicly available data from mass media (Kernasyuk, 2024) indicate that synthetic N production by the three main fertilizer producers (OSTCHEM, Azot, Rivneazot) in the first half of 2024 was reported to be at least 497 Gg N (*i.e.*, projected annual production is similar to that of 2019-2021 - *ca.* 984 Gg yr^{-1}; **Supplementary Fig. 9 -** as reported by FAO), with 58% produced in the form of urea ammonium nitrate (UAN) solution. Based on reported wholesale prices for various synthetic N fertilizers (ammonium nitrate, calcium ammonium nitrate (CAN) and UAN) produced in Ukraine (Superagronom.com, 2024), we calculated the average price of one kg of N in synthetic fertilizer, which was around USD 1.32 as of June 2024.

Manure application deficits.

The dramatic decrease in the application of manure (organic fertilizers) since independence in 1991 was caused by two main factors: (i) a sharp decline in livestock farming (Supplementary Fig. 6), leading to a decrease in manure production (Supplementary Fig. 11), and (ii) the decoupling of animal husbandry from cropping systems following the collapse of collective farm agriculture in Ukraine (Lerman, 2008). Despite the recovery and subsequent rise of the poultry sector, the total amounts and utilization of animal manure in the country are limited, along with the near absence of proper manure management and control in Ukraine. The poultry sector itself is thought to be poorly connected with cropping systems, with lack of transparency, and a relatively low share of poultry in total manure N production across the country (~23% in 2021; Supplementary Fig. 11).

Our calculations (**Supplementary Fig. 11**) indicate that the Ukraine's animal husbandry sector produced around 730 Gg N manure, with 59%, 22%, 18% and 1% excreted by cattle, poultry, pigs and sheep & goats, respectively. This comprises a significant amount of N, equal to about 74% of domestically produced inorganic N fertilizers. Based on the estimated manure N production (**Supplementary Fig. 11**) and data on manure N application to farmland (**Supplementary Fig. 7**), we estimated that the share of manure N applied in manure N excreted likely was *ca.* 10% in 2021 (**Supplementary Fig. 10**), while the share of organic N fertilizer (largely manure) in the total N application in 2021 was *ca.* 5% (**Supplementary Fig. 7**). For comparison, these shares ranged between 32-38% (**Supplementary Fig. 10**) and 41-52% (**Supplementary Fig. 7**), respectively, during the period 1986-1990.

We estimated that only 10% of the total amount of manure N excreted was applied to agricultural fields in 2021, while the rest (about 90%) remained unutilized (no records on its further fate are available), so that it was probably wasted, being emitted to the atmosphere as ammonia (NH_3), di-nitrogen (N_2) and nitrogen oxides (N_2O, NO_x), as well as run-off and leached to the hydrosphere, thereby contributing to environmental pollution (see **Supplementary Fig. 10**). The total economic loss in the agricultural sector due to unutilized manure was estimated at USD 2.2 billion for unused NPK in 2021 (**Fig. 3**), based on the average wholesale prices of synthetic N, P and K in Ukrainian market in June 2024 (Agro-Pritok, 2024; Demetra-Agro, 2024; Dobrivo.com, 2024; Makosh, 2024; Superagronom.com; UAPG, 2024). This includes USD 866 million for unused N at USD 1.32 per kg N, USD 530 million for unused P at USD 2.76 per kg P, and USD 780 million for unused K at USD 1.43 per kg K (see **Fig. 3; Supplementary Fig. 10**). Although manure N was also not very efficiently utilized during 1986-1990 (as already mentioned: from 32% to 38%), the large amounts of manure N produced and the strong integration of the animal production sector with cropping systems in collective farms during that period made it possible to contribute significantly to the agricultural demands for nutrients and organic matter (**Supplementary Fig. 10**). By 2023, due to increased fertilizer prices, we noticed again a positive trend in manure use in cropping systems, but its share remains limited at 12% (**Supplementary Fig. 10**).

Note 2.2: Soil degradation and the current state of soils in Ukraine

Soil organic matter (SOM) as key indicator of soil quality.

Commonly, a distinction is made between mineral soils and organic soils. Organic soils (or peat soils) have a very high organic matter content (>20% in at least the top 40 cm) and naturally occur under very wet conditions; the area of peat soils in Ukraine is very small and therefore peat soils are not discussed here further. Mineral soils consist of mineral compounds (including sand, silt and clay), organic matter and soil organisms. On a volume basis, minerals make up about 45-50%, soil organic matter (SOM) about 2 to 5%, soil organisms (including bacteria and fungi) ~1%, while the remaining 40-50% are soil pores filled with air and water. Soil organic matters gives mineral soils often a dark color; the darker the soil the higher the SOM content. The dark color of the SOM-rich and dominant soil type in Ukraine is reflected in the name: Chernozems, which means black soils. About 60% of the total agricultural land and about 73% of the arable land in Ukraine are covered by Chernozems. These deep soils are highly fertile, have neutral soil pH (pH 6.5 to 8.0) and are productive naturally, because of the parent material, the climatic conditions, natural grassland vegetation and soil formation processes. Conversely, soils with low SOM content are considered to be less fertile and are vulnerable to erosion and degradation (Russell, 1973; Lehmann and Kleber, 2015).

The soil organic matter content and soil pH are considered to be the most important indicators for soil quality. Soil scientists have for long tried to unravel the process dynamics of organic matter and its dark color in soils. The traditional concept of "**humus**" as a chemically distinct, persistent material formed through the synthesis of large-molecular-weight "**humic substances**" via humification of organic compounds has been fundamentally challenged by modern soil science. Historically, humus was perceived as a stable end-product of the decomposition of crop and root residues, animal manure and soil organisms. It was thought to be dominated by recalcitrant macromolecules which can be isolated through alkaline extraction methods (Lehmann and Kleber, 2015). However, advances in analytical techniques, such as nuclear magnetic resonance spectroscopy and mass spectrometry, reveal that these "**humic substances**" are artifacts of

extraction procedures rather than intrinsic soil components. Instead, SOM is now understood as a dynamic continuum of progressively decomposing organic compounds, influenced by microbial activity, mineral interactions and environmental conditions (Sutton and Sposito, 2005; Lehmann and Kleber, 2015). This emergent view aligns with the "**soil continuum model**" (SCM), which emphasizes microbial accessibility and protection mechanisms over inherent chemical recalcitrance. Sutton and Sposito (2005) further dismantle the traditional paradigm by proposing that humic materials are transient, supramolecular associations of low molecular mass components stabilized by hydrophobic and hydrogen-bonding interactions, rather than discrete polymers. Consequently, the term "**humus**" in modern terminology reflects not a chemically unique entity but a heterogeneous mixture of organic fragments at varying decomposition stages, integrated with microbial biomass and mineral matrices. This shift underscores the need to abandon outdated "**humification**" frameworks and prioritize research on microbial ecology, spatial architecture and solubility dynamics to advance predictions of soil carbon cycling, water quality and climate feedbacks.

Soil degradation and SOM loss.

The Food and Agriculture Organization (FAO) has highlighted significant concerns regarding soil degradation in Ukraine, particularly the loss of SOM. An FAO report (2019) noted that soil erosion by wind and water affects approximately 13.4 million hectares, including 10.6 million hectares of arable land. Furthermore, the FAO indicated that degraded and unproductive arable land in Ukraine exceeds 20% (more than 6.5 million hectares) of the total arable land, with annual soil loss due to erosion ranging from 300 to 600 million tons (FAO, 2018). Soil degradation through SOM loss and erosion mainly occur as a result of annual soil cultivation, growth of annual crops, without winter crops or intercrops. As a result, soils are bare for large parts of the year, and then vulnerable to wind and water erosion.

It is noteworthy that conventionally in Soviet and later Ukrainian soil studies (up to the present), humus content rather than soil organic matter (SOM) is reported. Therefore, we will use ‘humus content’ (%) as the originally reported data, assuming that the trend of change reflects SOM content. Also, in Soviet and Ukrainian studies, the average ratio of humus content to soil organic carbon in black soils was conventionally taken as 1.88 (*e.g.*, Ponomareva and Plotnikova, 1980; Chesnyak, 1983; Orlov, 1990).

Baliuk *et al.* (2021) made an assessment of the soil status and soil threats in Ukraine. They estimated that the loss of humus (and hence SOM) and nutrients occurs on 43% of arable land, soil compaction on 39%, surface crust formation on 38%, water erosion (both surface and linear) on 20%, acidification on 14%, waterlogging on 14%, deflation on 11%, contamination with radionuclides on 11%, pesticide contamination on 9%, heavy metal contamination on 8%, salinization and alkalization on 4% of the total arable land area in Ukraine. Svitlychnyi *et al.* (2022) emphasized the significant role of water erosion, particularly surface wash and linear erosion on bare soils, which occurs following rainfall and snowmelt, and leads to a decline of land productivity and increase of soil degradation. Water erosion affects approximately 38.4% of agricultural land across Ukraine, with 40% of arable land being eroded (Baliuk *et al.*, 2010). Among these eroded lands, 4.5 million hectares are classified as moderately or strongly eroded soils, including 68,000 hectares where the humus horizon has been completely lost (Baliuk and Medvedev, 2012). In the 1980s-1990s, the estimated average annual soil loss from arable lands in Ukraine exceeded 15 t ha^{-1}, ranging from 5 to 30 t ha^{-1} yr^{-1}. The average annual loss of humus is 0.5 t ha^{-1}, and nutrients are lost at a rate of 0.6 t ha^{-1}, which is not compensated by the use of fertilizers (Baliuk *et al.*, 2009). Whereas, an agro-ecological assessment for the transition of the agricultural sector in Ukraine (Mishchenko and Gumeniuk, 2006) estimated soil organic matter loss at slightly higher rates, on the order of 0.6–1.0 t ha^{-1} yr^{-1}, during the 1990s, consistent with an overall 5-6% reduction in topsoil humus content over that period (**Supplementary Fig. 12**).

In addition to water erosion, other detrimental processes relate to the intensification of soil cultivation and the associated loss of SOM and degradation of the soil structural-physical properties of Chernozems. Next, large and heavy machinery have induced (sub)soil compaction, leading to decreased water permeability and reduced water release in the top soil.

In natural soils, the process of SOM build-up typically prevails over SOM mineralization, gradually accumulating organic matter. However, in intensively cultivated arable soils, this balance shifts towards intensified mineralization, reducing SOM content, which leads to increased risks of soil degradation. The cause of SOM loss in Chernozems thus stem from increased soil cultivation practices, growth of annual crops without cover crops, and possibly climate change. There is a need for crop rotations involving cereals and cash crops and cover crops, combined with minimum tillage. In addition, there is a need for appropriate inputs of NPK fertilizers, depending on soil fertility status and crop rotations. Where available, animal manure should be utilized by preference (Baliuk and Medvedev, 2012).

Estimates suggest that Ukraine's Chernozem soils have lost a significant portion of their SOM and humus content over the past century. Chesnyak (1983) estimated an average loss of 35-40% of the original humus content. In the 1930s, chernozems were reported to contain 6 to 9% humus, but these levels decline to below 6% in 1980s (Chesnyak *et al.*,

1983; Nosko, 1987; Nosko *et al.*, 1988, 1992). Later studies (Baliuk and Medvedev, 2012; Baliuk et al, 2021) found that over 140 years since the first measurements of humus content by V.V. Dokuchaev, the loss of humus (and hence SOM) in the Forest-Steppe region averaged 22% and in the Steppe region averaged 19.5%. The largest losses occurred during the 1970s, when the area planted with annual crops such as sugar beets and sunflowers significantly increased, combined with soil tillage after harvest. However, some SOM loss was mitigated by the application of manure combined with mineral fertilizers (which also stimulated crop growth, including crop residues, which were returned to soil). In subsequent years, reduced fertilizer use led to a gradual decrease in humus content (and consequently SOM content), from a mean of 3.36% between 1986 and 1990 to a mean of 3.14% between 2006 and 2010. It should be noted that the decline in livestock number decreased the need for straw as bedding material; instead the straw was returned to soil, which helped to slow down the decline in SOM content. However, it has been recently stated based on the results of long-term agrochemical monitoring (Romanova *et al.*, 2022; Romanova, 2023) that the humus content (and hence SOM content) of Ukrainian agricultural soils continued declining to 3.07% (2016–2020) on average across Ukraine (**Supplementary Fig. 12),** indicating ongoing SOM degradation through net mineralization and nutrient removal via harvested crops (*i.e.*, mining). It is noteworthy that all the data of agrochemical monitoring presented here were weighted averages at either the country or county scale for the full spectrum of agricultural lands, including croplands (rain-fed and irrigated), grasslands, pastures, fallows, orchards and peasant homesteads and gardens (Romanova *et al.*, 2022; Romanova, 2023). In 2016-2020, among the surveyed agricultural lands in Ukraine, soils containing 3.1-4.0% humus prevail (32.8% of total), followed by soils with 2.1-3.0% (29.0%). Soils with higher humus content (4.1-5.0%) account for 17.7%, while those with lower humus content (1.1-2.0%) make up 16.1% of the examined soils (based on average county-level data) (Romanova, 2023). During the period of 2016-2020, the humus content decreased in 10 regions, with the most significant changes occurring in the soils of Khersonska (S-KH), Khmelnytska (W-KM), Odeska (S-OD) and Kirovohradska (C-KI) counties (**Supplementary Fig. 13;** Romanova *et al.*, 2022; Romanova, 2023).

To sum up, years of intensive soil cultivation, growth of annual crops without cover crops and with the near absence of organic fertilizer use, have contributed to significant SOM losses in Ukrainian soils and to soil degradation. The latter was related also to soil erosion and soil compaction. To improve the SOM status of Chernozems in Ukraine, it is recommended to incorporate legumes, alfalfa and perennial grass crops into crop rotations and to strictly prohibit the burning of straw and crop residues. Effective measures to increase both the quantity and quality of SOM in soil include maintaining an organic matter balance through the use of animal and green manures, as well as recycled bio-based waste, minimizing soil disturbance (*e.g.*, no-till, strip-till, mini-till), optimizing crop rotation structures, mulching with plant residues and applying chemical ameliorants (where it is required).

Concentrations of plant-available nitrates, phosphates and potassium in Ukrainian soils.
Romanova (2023) also reported a slight increase in easily-hydrolyzed (assumed to be plant-available) N concentration (measured by the Cornfield method using 1M NaOH; DSTU 7863:2015) from 108 mg NO_3 kg^{-1} (2001-2005) to 111 mg NO_3 kg^{-1} dry soil (2016-2020). However, 90% of the surveyed soils were still characterized by low (101-150 mg NO_3 kg^{-1}) or very low (<101 mg NO_3 kg^{-1}) levels of easily-hydrolyzed N. This parameter appears to be highly variable throughout the year in agroecosystems, depending on factors such as soil microbial activity, agro-environmental conditions, sampling season and field management practices, and hardly can be used as a proxy of N status for agroecosystems.

During the 2016-2020 agrochemical survey (Romanova *et al.*, 2022; Romanova, 2023), 34.7% of the surveyed areas had an extractable labile (assumed to be plant-available) P content (measured by the Chirykov method using 0.5M acetic acid; DSTU 4115:2002; see below) of 51-100 mg P_2O_5 kg^{-1} dry soil, 33.4% had 101-150 mg P_2O_5 kg^{-1} dry soil, and 15.8% were characterized by an average to high content of mobile phosphorus compounds (151-200 mg P_2O_5 kg^{-1} dry soil). Additionally, 8.0% had more than 200 mg P_2O_5 kg^{-1} dry soil, while 6.1% had 21–50 mg P_2O_5 kg^{-1} dry soil, and 1.9% had less than 21 mg P_2O_5 kg^{-1} dry soil. The weighted average extractable labile phosphorus concentration increased from 105 mg P_2O_5 kg^{-1} dry soil in 1986-1990 to 117 mg P_2O_5 kg^{-1} dry soil in 2016–2020. However, decreases in phosphorus content were observed in specific counties, including Lvivska (W-LV), Odeska (S-OD), Sumska (C-SU), Khmelnytska (W-KM), Zaporizka (S-ZP), Chernihivska (W-CH) and Volynska (W-VO). The Chirykov method suggests that the average availability of extractable labile phosphorus content in soils should fall between 51 and 100 mg P_2O_5 kg^{-1} dry soil. This method, however, is most accurate for soils with low phosphorus-containing minerals and organics with a clay content of 40-45%, but often overestimates the actual P_2O_5 concentration in Ukrainian black soils (see below).

Monitoring over 2016-2020 revealed 28.4% of the surveyed areas to have an extractable labile (assumed to be plant-available) K content (measured by the Chirykov method using 0.5M acetic acid; DSTU 4115:2002; see below) of 121-180 mg K_2O kg^{-1} dry soil, 27.1% had 81-120 mg K_2O kg^{-1} dry soil, 18.0% had more than 180 mg K_2O kg^{-1} dry soil, and 17.6% had 41-80 mg K_2O kg^{-1} dry soil (Romanova *et al.*, 2022; Romanova, 2023). Additionally, 7.4% had 21-40 mg

K_2O kg^{-1} dry soil, and 1.6% had less than 21 mg K_2O kg^{-1} dry soil. The Chirykov method suggests that the average availability of extractable labile phosphorus (K_2O) content in soils should fall between 41 and 80 mg K_2O kg^{-1} dry soil. This method, however, is most accurate for soils with a clay content of 40-45%, but often overestimates the actual K_2O concentration in Ukrainian black soils (see below).

However, these results (Romanova *et al.*, 2022; Romanova, 2023) based on the Chirykov method (DSTU 4115:2002), should be interpreted with caution. It is well known that soils developed on loess rocks with heavy granulometric composition (clayed textures), such as chernozems, contain high amounts of phosphorus-containing apatites, feldspars and three-layer aluminosilicates. Although P and K in these minerals are not accessible to plants, they are extractable with acidic solutions, such as 0.5M acetic acid (Khrystenko *et al.*, 2008, 2013; Kramaryov *et al.*, 2015; Khrystenko, 2023). The number of K-containing minerals also increases with a higher proportion of fine particles in the soil, which can artificially inflate K content values. Therefore, the availability of labile P and K in chernozems is significantly overestimated, with actual levels likely being low to medium, which may be insufficient for plant growth in intensive agricultural systems, further triggering soil organic matter mineralization. This results in an artificial overestimation of the true availability of P and K for plant uptake and misinform farmers (Khrystenko *et al.*, 2008, 2013; Kramaryov *et al.*, 2015; Khrystenko, 2023). For instance, long-term field experiments have shown that after establishing a high phosphorus baseline in the chernozem soil and ceasing fertilizer application, the average annual decrease in P_2O_5 content was 4.8 mg P_2O_5 kg^{-1} dry soil (Khrystenko, 2023). Kramaryov *et al.* (2015) stated that the use of the Chyrikov method leads to a systematic overestimation of phosphate content by 40-80 mg P_2O_5 kg^{-1} in Ukrainian soils with a clay content over 45% and increased amounts of phosphorus-containing apatites. Extensive soil P testing is critical to avoid either over-application or soil stock depletion of P (Stutter *et al.*, 2012; Brownlie *et al.*, 2021). It is widely recommended to use more appropriate methods, such as the Olsen method (ISO 11263:1994) and the Karpinsky-Zamyatna method (DSTU 4727:2007) for P, and the Machihyn method (DSTU 4114:2002) for both P and K, to avoid overestimating the available P and K contents extracted from chernozems with elevated amount of phosphorus- and potassium-containing minerals/ substances (Khrystenko *et al.*, 2008, 2013; Kramaryov *et al.*, 2015; Khrystenko, 2023). Moreover, to utilize 'legacy' P stored in some agricultural soils, innovative solutions have been developed, including the use of phosphate-solubilizing microbes and phosphorus-efficient crop cultivars (Stutter *et al.*, 2012; Brownlie *et al.*, 2021).

Technical details on the long-term agrochemical monitoring in Ukraine.

Monitoring including procedures, sampling, sample averaging and analysis, as conducted by the state organization ‘Institute of Soil Protection of Ukraine’ since 1965, are here summarized to help readers better understand and interpret the results. This summary is based on the studies of Romanova *et al.* (2022) and Romanova (2023). Over the course of 59 years, 11 rounds of land surveys have been completed at five-year intervals, with the 12th round currently underway and expected to conclude in 2025. During the most recent (11th) round (2016–2020), approximately 10 million hectares of agricultural land, around 35% of Ukraine's total agricultural area, were surveyed. In total, 1,300,000 soil samples were collected and analyzed for 25 agrophysical, agrochemical, radiological and toxicological parameters, as described in Yatsuk and Baliuk (2019). Unfortunately, these results are not freely available; the only public information comes from brief press releases or presentations by Dr. Svitlana Romanova. Soil samples were taken according to procedure described in Yatsuk and Baliuk (2019). In brief, sampling was conducted throughout the calendar year over the five-year period for each round, ensuring that soil temperatures were above +5° C. The size of elementary plots, subdivisions of a field that were uniform in meso-relief and ground cover, ranged from 5-8 ha in the Polissia and Transcarpathia zones to 15-20 ha in the forest-steppe and steppe zones. Approximately 20-25 individual point samples (15 cm^3 each) were collected from the topsoil (0-20/30 cm depth, depending on A-horizon thickness) within each elementary plot, then combined to form a composite sample (~400-500 g). These composite samples were mixed and sifted to remove plant residues, stones and other debris. If 80% of a 50-ha area had similar soil texture and was under the same crop, composite samples were combined into an averaging sample (with reduced weight). For less homogeneous areas, samples were combined accordingly. The averaging samples were then dried for 10-14 days before being sent for laboratory analysis. According to Yatsuk and Baliuk (2019) and Romanova *et al.* (2022), humus content was determined using Tyurin’s method (DSTU 4289:2004). On average, the ratio of humus content (%) to soil organic carbon (%) in black soils (Cherozems) is suggested to be 1.88 (Chesnyak *et al.*, 1983; DSTU 4289:2004). Easily-hydrolyzed N was analyzed by the Cornfield method using 1M NaOH (DSTU 7863:2015), while labile P and K compounds were measured by the Chirykov method using 0.5 M acetic acid (DSTU 4115:2002). According to Yatsuk and Baliuk (2019), averaging samples from 50-ha areas were area-weighted and combined to calculate a field average (for fields larger than 50 ha). The field averages were then area-weighted and averaged across all fields belonging to a single agricultural company or farmer to derive an agrofarm average. Similarly, agrofarm averages within a single village administrative unit were area-weighted and averaged to generate a village average. This process was repeated to derive district, county and country averages. Note that the results are based solely on the surveyed areas, and the size and number of samples may vary between districts and counties.

Note 2.3: High N-mineralization rate in black soils (Chernozems)

In most soils globally, N depletion through mineralization of soil organic matter (SOM) can typically be offset by increasing N inputs, most often through addition of crop residues, manures and synthetic fertilizer applications. However, chernozems, which are highly fertile with SOM content ranging from 3-6% in the upper 30 cm (Kovda and Rozanov, 1988; Boincean and Dent, 2020), are known to experience high rates of N mineralization and SOM degradation as a result of soil cultivation. For example, mean annual losses of carbon (C) and N in the top 30 cm of cultivated soils in southern Ukraine have been estimated at 298-415 kg C ha^{-1} and 30-42 kg N ha^{-1} (Kovda and Rozanov, 1988; Bilanchin *et al.*, 2021). Studies from Moldova and Ukraine (Boincean and Dent, 2019; Bilanchin *et al.*, 2021) suggest that the N deficit in cultivated chernozems is difficult to fully address with synthetic N inputs alone, underscoring the need for strategies aimed at building or at least maintaining SOM (*e.g.*, by addition of crop residues and manures). In southern Ukraine, Odeska county, increasing synthetic N inputs across four crop rotation cycles (from 90 to 226 kg N ha^{-1} yr^{-1}) resulted in a gradual decrease in NUE, declining from 132% to 50%. The decrease was even more (from 76% to 44%) when accounting for soil organic N mineralization as an input (see Serra *et al.*, 2025; Medinets S., pers. comm.). Moreover, given the significant role of soil fertility in yield formation and the low NUE from synthetic N fertilizers, each crop rotation should aim to optimize the ratio between organic and synthetic N inputs. It is suggested to increase the share of organic fertilizer (animal and green manure, bio-based fertilizers from organic residues/wastes) in the total fertilizer applied by 20-50% to maintain SOM levels.

Note 2.4: Mean N, P and K balances at country scale

For all three cropping systems, we mainly showed a deficit in applied K and P, which also can be seen using FAOSTAT data (Ludemann *et al.*, 2024). Overall, under-application of N, P and K, with a minor share of organic nutrient inputs (**Supplementary Fig. 7, 17**), has led to prolonged soil nutrient stock degradation via SOM mineralization (*i.e.*, soil mining) for decades (**Supplementary Fig. 12, Note 2.2-2.3**). We thus see a major asymmetry in Ukrainian agricultural nutrient balances at the country scale, with recent surpluses for N in wheat and maize combined with substantial continued deficits for both P and K. It is estimated that on average a total of 135 kg N, 52 kg P and 106 kg K were mined from each hectare of soil for sunflower crops, 85 kg N, 138 kg P and 86 kg K per ha of wheat, and 6 kg N, 164 kg P and 140 kg K per ha of maize, over the period of 2000-2021 period (**Fig. 1**).

Note 2.5: Mean N, P and K balances at county scales

The annual average N, P and K balances over the period 2019-2021 varied dramatically between counties: up to 3.5 (maize), 9.9 (wheat) and 67.8 (sunflower) times for N per hectare of UAA; 11.0 (sunflower), 15.6 (wheat) and 22.6 (maize) times for P per hectare of UAA; and 32.8 (sunflower), 35.0 (wheat) and 43.3 (maize) times for K per hectare of UAA. Mean balances followed a general pattern of western > central > eastern > southern counties, with often non-symmetric responses in yield production per UAA (**Supplementary Fig. 14-16**).

Considering the large variations of nutrient balances between counties (**Supplementary Fig. 21-23**) with an asymmetric pattern for N, P and K (**Supplementary Fig. 14-16**), the local consequences differed as well. Counties with relatively high surpluses in N are expected to exercise high pressures on the environment.

Note 2.6: Mean N, P and K use efficiencies at country scale

Our analysis of nutrient use efficiencies at country level for the period 2000-2021 revealed that for wheat, N use efficiency (NUE) (51-88%) was within the suggested optimal ranges in 2003, 2010, 2012, 2013 and then continuously since 2017 according to the EUNEP (2015) approach (**Fig. 1**). Potassium use efficiency (KUE) was within the suggested optimum ranges in 2003 (51%) and 2018 (81%) only, but mostly remained more than 100% (up to 193%) during other years, indicating consistent soil K mining. Phosphorus use efficiency (PUE) showed little change with time; it was more than 100% (130-353%; except for 2003), indicating significant draw down of soil P stocks. The relatively lower values for NUE, PUE and KUE in 2003, 2010, 2012 and 2020 compared with adjacent years were related to relatively low crop yields resulting from drought and other climatic factors.

For sunflower decreasing values for NUE, PUE and KUE were observed from 2017 onwards. For maize, NUE values exceeded the 90% threshold in 2000, 2001, 2003 and 2011, while remaining between 51% and 87% in other years. However, values for PUE and KUE consistently remained well above 90%, indicating soil P and K mining (**Fig. 1**). This pattern reflects a broader trend in nutrient use efficiencies for most crop commodities in Ukrainian agriculture since the 1990s (Lerman *et al.*, 2008; Ludemann *et al.*, 2024), with soil P and K mining being a widespread phenomenon.

Note 2.7: Mean N, P and K use efficiencies at county scales

Wheat: Wheat yields were relatively high in the western and central regions, with yields exceeding the 80 kg N ha^{-1}, the desirable minimum yield threshold recommended by EUNEP (2016), with mostly optimal NUE experienced since 2009 and 2010, respectively, due to consistently higher N applications under more favorable climatic conditions (**Supplementary Fig. 18**). By contrast, yields remained relatively low, with variable NUE often fluctuating around 90%, in the eastern and southern regions; improvements were achieved in 2021, with yields above the threshold and optimal NUE. In the latter regions, the incidence of drought is more frequent than in the western and central regions. In all regions, PUE and KUE were generally well above 90%, indicating that soil P and K mining occurred almost throughout the whole country.

Maize: In all regions, NUE was within or around the suggested optimum ranges during 2007-2021. In western region yields exceeded the desirable minimum since 2015, while in the central region it was more variable (**Supplementary Fig. 19**). In the eastern and southern regions yield rates remained below the desirable threshold, with the exception of the southern region in 2021. PUE and KUE were generally above 90%, with some values near or within the optimum range during 2007-2013 in the western region.

Sunflower: In the western region, NUE remained within or slightly above the suggested optimum range over 2007-2021, meanwhile other regions mostly experienced NUEs above or slightly below the optimum (**Supplementary Fig. 20**). Both PUE and KUE showed an improving trend over time. PUEs have reached optimum levels consistently in the southern, eastern and western regions since 2017. Improvements in KUE were primarily seen in the western region (achieving optimum over 2009-2011 and 2018-2021) and in the central region (in 2019-2021).

Note 2.8: Detailed description of nutrient management scenarios

Scenario design and baseline calibration
We defined a set of forward-looking scenarios to explore how Ukraine's crop nutrient balances could evolve by 2030 under different management interventions (**Table 1; Supplementary Table 2, 3**). These include:

- S-0: A Business-as-usual (BaU) scenario (assuming a return to 2021 practices);
- S-w: An extended war disruption scenario (assuming prolonged fertilizer shortages at 2023 levels);
- S-1: An improved management scenario at 2021 yield levels (manure-enriched precision fertilization);
- S2: An improved management scenario, building on S-1, plus enhanced-efficiency fertilizers);
- S3: An improved management scenario involving the legume-based diversification, combined with S-2.

The baseline for all comparisons is 2021, the last pre-war year of normal production, which we use as an analogue for 'current' conventional BaU management. National nutrient balance data from 2021 for wheat, maize and sunflower, calculated using advanced national statistics (see Methods), were sourced from our dataset (**Fig. 1**) and cross-checked with literature.
For assessment of the scenarios, we compare them with targets for acceptability outlined by Medinets and Sutton (2025), which can be summarized as follows: (i) unavoidable N losses are currently estimated at about 10% of total N input, considered the minimum level that avoids the risk of soil N mining under conventional agricultural practices (EUNEP, 2016; Congreves *et al.*, 2021; Mi *et al.*, 2025); (ii) the upper acceptable threshold is suggested to be no more than twice the unavoidable N losses, aligning with the societal optimum N input framework (van Grinsven *et al.*, 2014). However, the implementation of existing innovations and the development of future ones should not treat unavoidable losses as inevitable, but rather aim to reduce them further, *e.g.*, through increased focus on precision N management and soil health.

S-0: Business-as-Usual (BaU) scenario
In 2021, Ukraine's average fertilizer application rates and yields resulted in moderate N surpluses for wheat and maize (and a slight surplus for sunflower), alongside P and K deficits for those crops (and only minor P and K surpluses for sunflower). For instance, on average, wheat received 136.7 kg N ha^{-1} yr^{-1} (of which ~122.3 kg N came from synthetic

fertilizers and ~2.2 kg N from manure) and yielded ~4.5 t ha^{-1} yr^{-1} grain, removing ~93.9 kg N ha^{-1} yr^{-1} in the harvest, leaving an estimated N surplus of ~42.8 kg ha^{-1} yr^{-1}. Maize showed a similar N surplus (~41.9 kg ha^{-1} yr^{-1}). By contrast, P and K inputs in 2021 were substantially less than crop offtake for the cereals, *e.g.*, wheat received 11.2 kg P ha^{-1} yr^{-1} and 15.2 kg K ha^{-1} yr^{-1}, but removed 17.2 kg P and 20.9 kg K ha^{-1} yr^{-1}, respectively, forming a deficit of around 6 kg ha^{-1} yr^{-1} for both nutrients. Even larger deficits were observed for maize: 9.8 kg P ha^{-1} yr^{-1} and 7.6 kg K ha^{-1} yr^{-1}. These imbalances align with previous studies (FAO, 2005; Lerman, 2008; von Lampe *et al.*, 2014; Ludemann *et al.*, 2024), which noted Ukraine's tendency toward sufficient or excessive N application, alongside under-application of P and K, which is likely a legacy of cheap domestic N fertilizers and costly imported P and K (**Table 1, Fig.3; Supplementary Table 2, 3, Fig. 24, 25**).

Surprisingly, sunflower systems were nearly balanced by 2021, with acceptable annual nutrient surpluses (12.4 kg N ha^{-1}, 3.0 kg P ha^{-1}, and 1.1 kg K ha^{-1}), while maintaining nutrient use efficiencies within desirable ranges (84% for NUE, 77% for PUE and 94% for KUE).
We use the 2021 balance as the starting point for S-0 and as the reference for improvements in S-1, S-2 and qualitative S-3. For context, a 'no improvement' BaU trajectory (S-0) implies continued soil nutrient mining (annual negative P and K balances) and ongoing N losses, a situation recognized as unsustainable (Lerman *et al.*, 2008; Boincean and Dent, 2019; Ludemann *et al.*, 2024).

S-w: Extended war disruption scenario
The war disruption scenario (S-w) considers the case if 2023's war-induced low fertilizer use were prolonged, with continued extreme nutrient deficits. Under 2023 conditions, wheat, maize and sunflower had negative annual N balances (around 27.0, 5.8 and 17.2 kg N ha^{-1}, respectively), annual P deficits (around 13.1, 14.8 and 5.1 kg P ha^{-1}) and K deficits (roughly 14.3, 16.2 and 9.2 kg K ha^{-1}, respectively). Projected over 2024–2030, such deficits would accumulate to roughly 40–189 kg N ha^{-1}, 35–103 kg P ha^{-1}, and 65–113 kg K ha^{-1} being mined from soils per hectare, eventually impairing yields (**Table 1, Fig.3; Supplementary Table 2, 3, Fig. 24, 25**). Indeed, historical observations in Ukraine show that continuous nutrient deficits have led to steady declines in soil organic matter and incipient yield reductions (Chesnyak *et al.*, 1983; Nosko *et al.*, 1988, 1992; Lerman *et al.*, 2008; Baliuk *et al.*, 2009, 2021; Baliuk and Medvedev, 2012). This worst-case scenario provided the motivation for exploring the intervention scenarios described below.

S-1: Manure-enriched precision fertilization scenario
Scenario S-1 emphasizes increased manure recycling to substitute a portion of synthetic fertilizer, implemented through site-specific, crop-tailored fertilization practices. We operationalized S-1 based on the 'Smart Fertilizer Planner' (SFP) concept (**Supplementary Note 2.9**), which targets a 10-20% N surplus, with lower surpluses under low-emission agricultural practices (Medinets and Sutton, 2025), and near-zero surpluses for P and K. Literature on precision nutrient management suggests that better matching of nitrogen supply to crop demand can typically reduce N requirements by ~10% without yield loss (Sapkota *et al.*, 2019; Brownlie *et al.*, 2024).

Accordingly, as a starting point, we reduced the 2021 total N input rates for wheat and maize by 10%, solely through a decrease in synthetic N fertilizer use. For sunflower, total N input remains at the BaU level. We then increased the share of manure-N to 30% of total fertilizer N input by substituting part of the synthetic fertilizer, resulting in a total 37% reduction in synthetic N fertilizer use in each of the studied cropping systems. We also assume improved spatial targeting of N within fields and across regions, alongside low-emission technologies, enabling modest reductions in total N use while maintaining yields (Sutton *et al.*, 2022; Brownlie *et al.*, 2024), although not quantitatively modelled in the scenario.

For wheat and maize, which faced nutrient deficits in P and K, we implemented crop-specific strategies to meet the SFP targets. The remaining P shortfall, after accounting for manure, is addressed with synthetic P fertilizer: maintained at BaU levels for wheat and increased by 40% for maize to close the deficit. For K, we assume complete discontinuation of synthetic K use for wheat and a 52% reduction for maize. For sunflower, which showed only minor P and K surpluses in 2021, increased manure recycling allows for substantial reductions in synthetic P and K use, by 48% and 56%, respectively (**Table 1, Fig. 3; Supplementary Table 2, 3, Fig. 24, 25**).

However, substituting synthetic fertilizers with organic (manure-based) sources requires accurate estimation of plant-available nutrients. Since manure-derived nutrients are not fully crop-available in the year of application (~40% of manure-N in year one, ~20% in year two; ~70% of manure-P and 80% of manure-K in year one (with no data about the availability in the consecutive years; Shapiro *et al.*, 2021; Iqbal *et al.*, 2022), correction factors are applied to determine the gross application needed to meet net surplus targets.

For example, the total N input to wheat (synthetic + organic + deposition + N fixation) in 2021 was ~136.7 kg N ha^{-1}. In S-1, we target a 10% reduction, lowering total input to ~123 kg N ha^{-1} while maintaining ~93.9 kg N uptake. Manure-N input increases from ~2.2 kg ha^{-1} yr^{-1} in 2021 to ~33.0 kg ha^{-1} yr^{-1} in S-1 (equivalent to roughly 5–6 tonnes of mixed-origin manure per hectare, based on 2021 application patterns; **Supplementary Table 5**). This requires much better use of existing manure resources, which are currently wasted (Supplementary Fig. 10). **Supplementary Note 2.1** and the data in **Fig. 11** indicate that there would be sufficient manure available to meet this scenario according to current livestock numbers (see further details below). Synthetic N use drops from ~122.3 to ~77.1 kg ha^{-1} yr^{-1}. Of the ~123.0 kg total N input ha^{-1} yr^{-1}, only ~109.8 kg is estimated to be crop-available, accounting for 40% (~12 kg N ha^{-1} yr^{-1}) first-year manure mineralization and 20% (~6 kg N ha^{-1} yr^{-1}) carryover from the previous year. This leaves a small crop-available surplus (~16 kg N ha^{-1}) to offset unavoidable losses, assuming ~93.9 kg N uptake. The remaining crop-unavailable surplus (~13 kg N ha^{-1} yr^{-1}) is likely retained in soil, contributing to soil organic matter (SOM) build-up (Boincean and Dent, 2019; Baliuk *et al.*, 2021; Kraus *et al.*, 2024). The same principle applies for P and K: wheat receives ~20.9 kg P ha^{-1} yr^{-1} (up from 11.2), with 17.8 kg crop-available P matching ~17.2 kg P removal, as well as ~26.4 kg K ha^{-1} yr^{-1} (up from 15.2), with 21.1 kg crop-available K matching ~20.9 kg K removal. Similar adjustments are applied for maize and sunflower (**Table 1, Fig.3; Supplementary Table 2, 3, Fig. 24, 25**).

Overall, S-1 is expected to deliver more sustainable nutrient balances compared with BaU, with significant N surplus reductions, improved near-neutral P and K balances and potential soil health benefits. We also expect yield maintenance and reduced reactive N losses. This is supported by the meta-analysis of Ren *et al.* (2022a), who showed that substituting 20-50% of synthetic N with manure, on average, increased wheat yields by ~5% and reduced total N losses, with 49% lower ammonia (NH_3) volatilization in wheat, 13% reduced nitrous oxide (N_2O) emissions and ~50% less runoff N loss in maize systems. Additionally, improved fertilizer timing and placement (the '5R' practices) under S-1 may enhance nutrient use efficiency and reduce losses, although not explicitly modelled in this scenario.
Manure integration under S-1 addresses Ukraine's substantial underutilization of manure nutrients: only ~10% of total manure produced (excreted from farm animals) was applied to agricultural lands in 2021 (**Supplementary Fig. 10**). By adjusting manure use to crop-specific P and K needs, S-1 reduces reliance on costly imported fertilizers and improves soil health via added organic macro- (C, N, P, K) and micro-nutrients. We qualitatively assume gains in soil organic carbon under S-1, which could, over time, enhance nutrient retention and water-holding capacity, though these effects are not explicitly considered here.

S2: Enhanced efficiency fertilizers (EEFs) scenario
Scenario S-2 extends S-1 by incorporating enhanced efficiency fertilizers (EEFs), including nitrification and/or urease inhibitor-treated fertilizers (*e.g.*, urea-based products), conventional fertilizers combined with single/dual inhibitors and/or slow-release formulations to further reduce N losses. The intended outcome is that a greater fraction of applied N is taken up by crops, allowing for either yield increases, N input reductions or both.

Meta-analyses and reviews (*e.g.*, Thapa *et al.*, 2016; Cantarella *et al.*, 2018; Cui *et al.*, 2018; Klimczyk *et al.*, 2021; Ren *et al.*, 2023; Matse *et al.*, 2024) showed that nitrification inhibitors could reduce direct N_2O emissions by 30-50%, while urease inhibitors could lower NH_3 volatilization by 30-75% (on average around 54%). Also, Guardia *et al.* (2017a, b) and Recio *et al.* (2020) demonstrated that, alongside a 35–40% reduction in N_2O emissions, nitrification inhibitors, such as (i) DMPSA, (ii) DMPSA with or without urease inhibitor NBPT, and (iii) DMPP, were able to mitigate nitric oxide (NO) emissions by 76% from calcium ammonium nitrate (CAN), by more than 60% from urea, and by 60% from pig slurry, respectively. Yield responses vary: some studies reported slight gains (+5% on average) due to improved N retention (Lam *et al.*, 2018), while others find no significant yield change but reduced N fertilizer needs for the same yield.

In this scenario, we take a conservative approach prioritizing environmental benefits. We assume that EEFs enable farmers to maintain the same yield with ~10% less N compared to S-1, due to reduced N losses. Accordingly, we reduce total N inputs by an additional 10% for wheat and maize, and by 5% for sunflower in S-2 compared with S-1, solely through reductions in synthetic N fertilizer, *e.g.*, wheat in S-1 had a total N input of ~123 kg N ha^{-1} yr^{-1} (including deposition and N fixation); in S-2, this is reduced to ~111 kg N ha^{-1} yr^{-1} on an annual basis. Manure-N inputs remain the same as in S-1 (33 kg N ha^{-1} yr^{-1}), while synthetic N is lowered from ~77 to ~65 kg N ha^{-1} yr^{-1}. Inputs of P and K remain unchanged from S-1, assuming EEFs mainly affect N cycling (Brownlie *et al.*, 2024 and references therein), and P and K management is based on nutrient removal with yield.

These adjustments bring the estimated N surpluses in S-2 closer to the acceptable minimum (9–17 kg N ha^{-1} yr^{-1}), with only marginal surpluses of crop-available N (~3–4 kg N ha^{-1} yr^{-1}). For wheat, with ~94 kg N ha^{-1} yr^{-1} uptake and ~111 kg N total input ha^{-1} yr^{-1}, the surplus is ~17 kg N ha^{-1} yr^{-1}, approximately 61% lower than the ~43 kg N ha^{-1} yr^{-1}

surplus in the BaU scenario. Considering only crop-available N in S-2, the balance becomes even tighter, leaving only minimal residual mineral N in soil and/or subject to loss. Similar reductions in N surplus are seen for maize and sunflower (**Table 1, Fig.3; Supplementary Table 2, 3, Fig. 24, 25**).

These improved N balances are accompanied by increased crop uptake and further reduced N loss pathways, as EEFs stabilize fertilizer N (Thapa *et al.*, 2016; Guardia *et al.*, 2017a, b; Cantarella *et al.*, 2018; Cui *et al.*, 2018; Recio *et al.*, 2020; Klimczyk *et al.*, 2021; Ren *et al.*, 2023; Matse *et al.*, 2024). On average, the cumulative emission mitigation potential of S-2, building on S-1, is estimated to be ~77% for NH_3, ~48% for N_2O and at least ~60% for nitric oxide (NO). We expect similar reductions in Ukraine's arable systems, given the higher baseline N surpluses (and potentially N losses) under BaU practices.

By cutting N fertilizer rates and preventing rapid N transformations, S-2 is expected to significantly reduce gaseous emissions (NH_3, N_2O and NO) and nitrate leaching compared with S-0. One caveat is that, if farmers fully adopt EEFs, they may also realize some yield gains; if yields increase, N uptake would rise and the apparent surplus could decrease further, potentially turning to zero or even a slight deficit if not offset.
Regardless, S-2 represents a system with very high nutrient use efficiencies, estimated at 85-88% for total applied N, 83-88% for total P and 79-87% for total K. These values are at the upper end of what has been documented globally and are typically only observed in best-practice or experimental settings (Claessens *et al.*, 2024; Krauze *et al.*, 2024).

S-3: Crop diversification with legumes scenario
Scenario S-3 adds to the measures included in S-2 by also exploring the impact of introducing N-fixing legume crops into rotations as a strategic measure to supply biological N and reduce dependency on synthetic fertilizers. While our core dataset and the above scenarios treat each crop independently (annual static balances), S-3 requires consideration of multi-year crop sequences for accuracy. Although we indicatively include numeric data for S-3 in Supplementary Table 2-4, we largely estimate its effects qualitatively, supplementing S-2.

We assume that by 2030, a significant portion of Ukraine's grain area (around 20%) could be sown with leguminous crops (*e.g.*, soybeans, peas, beans or forage legumes), supported by appropriate policy incentives (see Actions). This affects nutrient flows in two main ways:
(i) During legume cultivation, biological N fixation (BNF) provides most of the N for that crop, greatly reducing or eliminating the need for (synthetic) N fertilizer on those fields;
(ii) The residual N benefit to the following crop allows for lower (synthetic) fertilizer rates on that subsequent crop.
Empirical data from Europe show that grain legumes like pea or faba bean can, on average, fix between 130–153 kg N ha^{-1} in their biomass over the growing season each year (varied by species and conditions) (Zander *et al.*, 2016), with a portion remaining in the soil after harvest. A review by Plaza-Bonilla *et al.* (2017) found that legumes contributed roughly 40–50 kg N ha^{-1} to subsequent wheat crops on average. We adopted a conservative credit of ~50 kg N ha^{-1} for the crop following a legume. In practice, this means that if wheat or maize follows a legume in rotation, farmers can reduce synthetic N by about 50 kg ha^{-1} without yield loss.

In our scenario framing, we applied this concept by envisioning that 1 out of every 4 years a cereal is preceded by a legume, giving an effective annualized N credit of ~15-16 kg ha^{-1} across the rotation. For simplicity, we incorporated this into S-3 by reducing N fertilizer inputs an additional ~15% beyond S-2 levels (which roughly equates to ~15 kg less N for cereal crops, in line with the credit). Thus, if wheat and maize received ~111 kg N ha^{-1} yr^{-1} in S-2, they might receive ~94 kg N ha^{-1} yr^{-1} in S-3, in addition to benefiting from 15 kg N ha^{-1} yr^{-1} available from the preceding legume crop. Sunflower, which often follows cereals, might indirectly benefit from a preceding legume's residual N as well (we roughly estimate this benefit at a lower rate of ~10 kg N ha^{-1} yr^{-1}) (**Fig.3, Table 1**).

The precise numbers are less important than the trend: S-3 pushes synthetic N inputs down to ~61% (when combined with S-2) and to 52–56% (when combined with S-1 only) below the 2021-based BaU scenario (S-0) for the studied staple crops by 2030, effectively replacing that portion with biologically fixed N. This means Ukraine's average synthetic N use in 2030 could be more than halved (61% reduction) for wheat, maize and sunflower compared to BaU practices, if crop diversification with legumes is combined with manure-enriched precision agriculture and EEF practices (**Fig.3, Table 1**).

We keep yield assumptions in S-3 the same as in S-0, S-1 and S-2, while acknowledging the well-known rotation yield boost: cereals after legumes often yield more than cereals after cereals, due to improved soil conditions and pest/disease breaks (Zander *et al.*, 2016; Geng *et al.*, 2023).

Another benefit pertains to P: some legumes can acidify the rhizosphere and mobilize sparingly soluble P, effectively tapping into less-available soil P pools (Yu *et al.*, 2021). While we did not quantify this, it implies that legume rotations might partially compensate for lower P fertilizer inputs by increasing P availability from the soil, at least in the short term (Jemo *et al.*, 2006; Yang *et al.*, 2021; Yu *et al.*, 2021). However, some studies reported no increase in soil P availability under/ after legumes (Maltais-Landry *et al.*, 2015).

Overall, S-3, when built on S-2, essentially achieves a near-zero balance for crop-available N at the national scale for key staple crops in rotation (Supplementary Table 2). The additional N fixed biologically is largely removed in harvest or remains in soil organic matter, with minimal losses (Yang *et al.*, 2024; Sharma *et al.*, 2025). In the long term, a small fraction of fixed N may build up soil N capital, which is desirable for soil health.

It is worth noting that even in S-3, prudent management is essential: high legume proportions could risk N surpluses if residual N is not accounted for, or could deplete soil moisture in dry regions, affecting the next crop (Pang *et al.*, 2018; Liu *et al.*, 2024). Our scenario assumes best practices in legume management (optimal varieties, inoculation and proper termination to maximize N benefits).

The environmental upside of S-3 is significant: by reducing synthetic N fertilizer manufacturing and application, it lowers greenhouse gas and reactive N emissions, as well as upstream energy use. In-field N losses also drop further (Yang *et al.*, 2024). A European study estimated that expanding legume area can reduce agricultural N_2O emissions by up to 40–50%, while also cutting fertilizer-associated CO_2 emissions (Zander *et al.*, 2016).
While achieving S-3 at scale would require substantial shifts in cropping systems (and probably advanced markets for legumes), it represents a regenerative pathway aligned with EU sustainable agriculture goals.

Supporting data and calculations

The quantitative scenario values used in our analysis are summarized in **Table 1** and **Supplementary Table 2, 3**. This table presents, for each crop and scenario, the estimated total N/P/K inputs, synthetic fertilizer N/P/K inputs, manure N/P/K inputs, total crop-available N/P/K inputs, yield N/P/K outputs and resulting nutrient balances (surpluses/deficits) in absolute units, percent changes and cumulative balances over 2024–2030. We derived these by applying the percentage adjustments and allocation rules described above to the 2021 baseline data for S-0, S-1, S-2 and qualitative S-3.

For example, **Table 1** shows the annual total N input to wheat dropping from 136.7 kg N ha^{-1} in S-0 to ~123.0 kg N ha^{-1} in S-1 (10% reduction) and to ~110.7 kg N in S-2 (10% further reduction vs S-1), with a further notional drop to ~94.1 kg N ha^{-1} (excluding N input from the preceding legume's BNF) in S-3 (15% further reduction vs S-2). The annual P input rises from 11.2 kg ha^{-1} (S-0) to 20.9 kg ha^{-1} in S-1/S-2/S-3, eliminating the P deficit. Similar entries are provided for K, and for N/P/K in maize and sunflower (**Table 1; Supplementary Table 2, 3**).
We include the cumulative nutrient surpluses or deficits over 2024–2030 for the BaU (S-0) and the worst-case (S-w; based on 2023 data) projections, to illustrate the magnitude of soil nutrient stock changes if current trends persist. For example, under S-0, Ukraine's wheat would accumulate an N surplus of roughly $7\times42.8\approx300$ kg N ha^{-1} over 2024–2030, whereas P and K would be depleted by $7\times6=42$ kg P ha^{-1} and by $7\times5.6\approx39$ kg K ha^{-1}, respectively, in that period (**Table 1; Supplementary Table 2, 3**).

We stress that these are approximate estimates, as year-to-year yield fluctuations are not treated (for simplicity, we assumed 2021-level yields each year). Nonetheless, the cumulative figures highlight how quickly soil fertility could deteriorate in the absence of corrective action: losses of tens of kg per hectare of P and K each year could exhaust even high initial soil reserves within the coming decade(s) (**Supplementary Table 2, 3**).
For N, cumulative surpluses directly translate to pollution potential and waste of nutrient resources. Under S-0 (BaU), a cumulative surplus of ~300 kg N ha^{-1} for wheat, ~293 kg N ha^{-1} for maize and ~87 kg N ha^{-1} for sunflower across Ukraine's wheat (7.1 million ha), maize (6.7 million ha) and sunflower (5.5 million ha) areas as of 2021 would imply on the order of 4.6 million tonnes of reactive N potentially emitted or leached over 2024–2030, a portion of which would impact air quality (via NH_3 and NO), water quality (*e.g.*, freshwater and Black Sea eutrophication) and climate (via N_2O) (**Table 1**).

In contrast, enormous cumulative deficits translate to soil degradation potential. Under the extended war disruption scenario (S-w), assuming continued war-related disruptions, large cumulative deficits of N (189, 41 and 120 kg N ha^{-1}), P (320, 197 and 102 kg P ha^{-1}) and K (218, 114 and 110 kg K ha^{-1}) for wheat, maize and sunflower, respectively, across reduced crop areas in 2023 (wheat: 4.7 million ha; maize: 4.0 million ha; sunflower: 5.2 million ha) would imply on the order of 1.7 million tonnes of N, 2.8 million tonnes of P and 2.1 million tonnes of K potentially mined from Ukrainian soils between 2024 and 2030 (**Table 1; Supplementary Table 2, 3**).

Our sustainable scenarios, S-1 and S-2, drastically reduce the waste of N resources compared with S-0 (by 48–92% of crop-available N) or even eliminate the S-w deficit, while promoting SOM build-up due to longer retention and/or incorporation of crop-unavailable, manure-derived organic nutrients in soil. Even more environmental and economic benefits are anticipated under crop diversification with legumes (S-3).

Assumptions and limitations

These scenario calculations are intended as illustrative projections, not precise estimates. We made several simplifying assumptions:

(i) **Yield constancy**: We held crop yields constant at 2021 levels in all scenarios (except acknowledging slight rotational boosts in S-3, and the worst-case war scenario S-w, based on 2023 yield levels). In reality, yields by 2030 could change due to genetic improvements, climate trends, other socio-economic factors or further war impacts (which may be similar or different to those projected in S-w). Higher yields would demand more nutrients but also result in higher uptake, possibly maintaining similar balances if managed well. Our constant-yield approach isolates the effect of management practices on nutrient balances. This is a necessary simplification, given the lack of field measurements in Ukraine for the practices proposed in the scenarios.

(ii) **No phased adoption modelling**: We did not explicitly model year-by-year transitions or adoption rates. Instead, we effectively assume that by 2030 the practices are fully adopted (a snapshot analysis). A more detailed dynamic model might, *e.g.*, phase in manure use gradually or simulate yield dips in early years under nutrient deficits. Here, we compare end-state scenarios for clarity and simplicity.

(iii) **Simplified surplus interpretation**: Our nutrient balance framework does not detail all loss pathways. The 'surplus' in our tables represents a lump sum that could be lost as reactive N emissions, nitrate leaching and/or minor soil accumulation (unless organic- or manure-enriched fertilization is applied). When we report annual 'N surplus = 43 kg N ha^{-1}', it does not specify how much is lost (and how) versus temporarily stored in soil. For scenarios (namely S-0 and S-w), we interpret reductions in surplus as reductions in losses, which is reasonable if soil nutrient stocks are not building up. In the sustainability scenarios (S-1, S-2 and qualitative S-3), where manure-enriched fertilization is applied, we assume surpluses of crop-available N largely represent losses, while crop-unavailable fractions likely contribute to building soil nutrient stocks. In the case of actual yield increases under S-2 and S-3 (where balances are tight and may approach near zero or even slightly negative), we assume that slight deficits can be temporarily offset by mineralization of soil organic matter (until the yield gain is recorded). Once that happens, we assume decision-makers would in practice fine-tune synthetic and/or organic inputs to avoid sustained nutrient mining.

(iv) **Manure availability**: We assumed that manure could supply up to 30% of crop N needs by 2030. This is contingent on major improvements in manure management infrastructure. Currently, logistical and regulatory barriers in Ukraine make it difficult to collect and distribute manure at this scale. Our scenario presumes a concerted effort to invest in low emission practices for manure transport, storage and application, possibly via manure cooperatives or biogas digestate systems, *e.g.*, at the commune/ district level as implemented in Romania under the EU and World Bank support (INPC, 2011, 2019, 2025). If only 15% substitution is achieved by 2030, the benefits would be proportionally smaller (though still significant). In addition, manure application rates and timing should comply with existing regulations under the EU Nitrates Directive (91/676/EEC) to minimize nitrate pollution of surface and groundwater, particularly within designated Nitrate Vulnerable Zones (Serra *et al.*, 2024).

(v) **EEF feasibility**: The enhanced-efficiency fertilizer scenario (S-2) assumes that these products are effective under Ukrainian conditions (*e.g.*, soil type, temperature) and that farmers can afford them. The cost of these advanced fertilizers/inhibitors could pose a barrier; however, if fertilizer prices remain high, even a ~10% saving in N use could economically justify their adoption. We did not perform a detailed economic analysis, but note that Lam *et al.* (2018) found the cost per kg of dry matter produced was similar for inhibitor-treated vs. regular urea when accounting for yield gains in their trial. Governmental subsidies may also help offset early-stage costs.

(vi) **Legume scenario generalization**: The crop diversification with legumes scenario (S-3) represents a broad-brush analysis of rotational effects. Actual outcomes would depend on which legume species are used, the area planted and rotation frequency. For example, soybeans remove much of the N they fix in the harvested grain, often leaving less residual N than peas or beans (Yang *et al.*, 2024; Sharma *et al.*, 2025). We based our assumptions on average residual N benefits from a mix of grain legumes. If farmers rely mainly on soybeans (a common, profitable legume), fertilizer savings for subsequent crops might be smaller than if using field pea (which leaves more N in residues). A mix of grain and green-manure legumes might maximize benefits (Sharma *et al.*, 2025); however, green manures require taking land

out of cash-crop production for a season. Growing cash crops might be subsidized by government, as in neighboring Romania (INPC, 2011, 2019, 2022). Our S-3 scenario implicitly assumes the use of grain legumes, so that there is economic output as well.

(vii) **Secondary environmental effects not modelled**: We did not model secondary environmental impacts such as changes in soil moisture or pest populations due to new practices. For example, additional manure application could increase soil moisture retention (S-1, S-2 and S-3); legumes could reduce the need for synthetic pesticides (some break disease cycles and lower N fertilizer use often attracts fewer pests due to reduced foliage density; S-3). These could provide additional benefits or introduce new management challenges beyond the nutrient-centric scope of our model.
Despite these uncertainties, our multi-scenario approach provides a grounded estimate for policymakers and farmers of what level of improvement could be achievable by 2030. We anchored each adjustment in documented research findings to ensure plausibility. The results directly inform the policy recommendations in our main text, offering an evidence-based trajectory rather than qualitative speculation.

Note 2.9: Smart Fertilizer Planner

Principle and usage.
The proposed region-tailored, variety-specific **Smart Fertilizer Planner (SFP)** tool is based on a simplified nutrient balance principle, incorporating a ‘10-to-20%-surplus’ for N (Medinets and Sutton, 2025) and near ‘zero-surplus’ approach for P and K. This balance is calculated between the total input, which includes synthetic and organic fertilizer applied by farmers alongside the sum of mean atmospheric inorganic N deposition and N fixation by free-living organisms (provided as a ‘constant’; N deposition is updated upon new data become available in EMEP reports), and the total output, which includes crop yield (and by-product if straw is removed from the field). Both inputs and outputs are expressed in pure elemental units: kg N ha^{-1} yr^{-1}, kg P ha^{-1} yr^{-1}, and kg K ha^{-1} yr^{-1}.

It is assumed that a 10-20% N surplus, together with variability in atmospheric nutrient deposition, including unaccounted organic NPK deposited from the atmosphere, is roughly within the range of unavoidable nutrient losses and societal optimum input levels (van Grinsven *et al.*, 2013; Medinets and Sutton, 2025). The unavoidable N losses depend on the agricultural practices and fertilizer types/rates used, and tend to approach near zero with agro-technological advances in future (Medinets and Sutton, 2025). Losses are typically higher for N and expected to be marginal for P and K (see 'Limitations and Assumptions' below). In the case of a manure-enriched fertilization scheme (where >10% of total fertilizer-derived N is applied as manure), correction factors for crop-availability of manure-derived nutrients should be made. It is assumed that crop-available manure-N is approximately 40% in the first year and 20% in the second year after application (Shapiro *et al.*, 2021; Iqbal *et al.*, 2022). This suggests that at least around 60% of applied manure-N is supposed to be effectively utilized by crops in a rotation. The remainder is presumed to accumulate in soil, contributing to soil organic matter (SOM) build-up, and/or be partially lost (Krause *et al.*, 2024). Upon field application, N losses from manure-based systems vary widely and depend on manure type and treatment methods, and the use of low-emission application techniques (Brownlie *et al.*, 2024, and references therein). Crop availability of manure-derived P and K is assumed to be approximately 70% and 80%, respectively, during the first year of application (Shapiro *et al.*, 2021; Iqbal *et al.*, 2022).

The tool is suggested to be available as mobile app and a downloadable spreadsheet. It would provide farmers with easy-to-use yet precise estimation for NPK fertilizer input requirements tailored to their region and crop varieties, based on data from the top-performing crops over the past 3-5 seasons.

The SFP would help calculate annual field-scale nutrient balances and estimates nutrient use efficiency after harvest using yield data. A key element of the SFP is the collection of crop yields from varieties grown in a specific region, followed by analysis of their NPK content to develop a region-tailored, variety-specific NPK removal repository. This repository should be updated whenever new varieties are introduced in the region. Another important aspect is the analysis of NPK content in organic fertilizers applied (if relevant), to at least establish an initial inventory for the region or country. Ideally, this information should be updated regularly, or, as an advanced option, it could be provided on a regular basis by organic fertilizer suppliers. This approach can be implemented at field-to-regional levels, supporting farmers and policymakers in various counties and georegions for rain-fed cropping systems (*i.e.*, including internationally beyond Ukraine). It can also be used for irrigated crops; in this case, the NPK content applied through irrigation water (*i.e.*, NPK concentration in irrigated water multiplied by the amount of water applied) should be added to the input category for more precise calculations. Moreover, the SFP would be adaptable to entire crop rotation cycles, making fertilizer planning more accurate and comprehensive. In this case, it would account for nutrient surpluses or deficits from preceding crops to adjust applications for subsequent ones.

The SFP is designed to support adoption of precision farming principles for crop nutrition based on crop requirements and pedoclimatic conditions at field-to-farm scale. Key elements include the so called '5R approach' to nutrient inputs (right source, rate, application time, application place and application method) and require the following actions to foster successful implementation, including (i) improving access to latest technology, information and required inputs; (ii) access to reliable soil analytical services, and the set-up of a soil fertility monitoring program for all cropland, (iii) evaluation of the existing crop-specific and soil fertility-based fertilization recommendations. Also, the nationwide field campaign is needed to inventory nutrient removal rates for the key crop varieties across georegions to increase the accuracy of the SFP (**Supplementary Table 3**). The establishment of regional advisory services would greatly facilitate adoption of precision fertilizer principles and implementation a wider range of Good Agricultural Practices (GAP) at the field-to-farm level. These would raise farmers' awareness of nutrient management issues by offering education and knowledge transfer, with tailored, region-specific strategies supported by simplified cost-benefit analyses and providing the required information for Smart Fertilizer Planner. Accompanying grants and loans to farmers for investment in nutrient management would catalyse change that can ultimately become self-sustaining (**Supplementary Note 2.10**).

Limitations and assumptions.
The proposed tool would generally assume that vegetation biomass benefits from crop residues returned to the field in previous years. In a simplest approach it may be estimated that up to 5%-surpass for N together with variation of atmospheric deposition of NPK, including unaccounted rates for organic NPK deposited (**Supplementary Note 3.2**), are roughly offset by unavoidable N emissions and potential NPK leaching or runoff (so long as straight urea is not used). However, the performance of the SFP may be substantially improved if evidence-based estimates for organic nutrient deposition and region-specific fertilizer-induced emission factors are available. Emissions of N are considered the primary loss pathway in black soil agroecosystems, while leaching of nitrate, phosphorus and potassium compounds is supposed to be less critical, though migration to lower soil horizons may occur (Kovda and Rozanov, 1988; Krupenikov *et al.*, 2011; Medinets *et al.*, 2016, 2021; Boincean and Dent, 2019).

Prerequisites and requirements.
To estimate NPK removal by crop yield for specific crop varieties, a database of NPK content for currently used varieties is essential. This database should be created and regularly updated as new varieties are introduced. Such information must be accessible to farmers, either through regional advisory services, online repository or mobile app.

The minimum requirements for successful use of the SFP tool would include:

(i) Replacing straight urea to prevent significant nitrogen losses via NH_3 volatilization.
(ii) Returning crop residues to the field as a source for the next year's vegetation biomass or, alternatively, accurately calculating removed amounts and replenishing them with (organic) fertilizers.
(iii) Ensuring availability of NPK content data for key crop varieties.
(iv) Accessing appropriate soil testing to monitor exchangeable P and K concentrations.

Further development.
The SFP is intended as a simple flexible solution that should be developed over time. With further research and advancements in technology, more precise, region-specific guidance could be developed, taking into account local pedoclimatic and socioeconomic conditions.

Other tools available.
Also, there are some other free and commercial tools available to farmers for various purposes, they are, sometimes, specific to the georegion, but often not specific to crop varieties in a given region and, unfortunately, do not cover Eastern European countries outside the EU. However, with region-tailored, variety-specific evidence-based data, they could be complimented to SFP or adapted as a separate tool for use in Eastern European countries.
Among them are the following:
(i) Farm Sustainability Tool for Nutrients (FaST) – a digital tool developed under the EU CAP to help farmers create nutrient management plans. It integrates existing (mainly satellite) data with manual inputs to provide customized recommendations on crop fertilization, aiming to deliver both economic and environmental benefits. It is intended for region-specific implementation across EU countries with governmental support.
(ii) NUTRI-CHECK NET Platform – an EU-funded initiative offering a three-step approach—Plan, Check & Adjust, and Review—for nutrient management. It essentially serves as an inventory of various tools and services to support farmers, agronomists, researchers, and the broader agricultural industry in sharing knowledge and exploring available N, P, and K nutrient management approaches for wheat, maize, and potato across Europe.
(iii) Planning Land Applications of Nutrients for Efficiency and the Environment (PLANET) – a nutrient management decision support tool for use by farmers and advisers in England, Wales and Scotland. It supports field-level nutrient planning and helps assess and demonstrate compliance with the Nitrate Vulnerable Zone (NVZ) rules.

(iv) CAFRE Crop Nutrient Calculator – a tool developed by the College of Agriculture, Food and Rural Enterprise (CAFRE) of Northern Ireland, based on soil nutrient analysis. It helps farmers create precise fertilization plans that meet crop nutrient requirements without excess. The tool emphasizes the importance of soil pH and nutrient stocks, and promotes low-emission techniques for slurry spreading.

Note 2.10: Farming during wartime

This section briefly describes the key challenges faced by Ukrainian farmers during the ongoing conflict, based on insights from farmers, media reports (Ecoaction.org.ua, 2023; FAO, 2023; tni.org, 2023; World Bank, 2023a,b; Latifundist.com, 2024; Superagronom.com, 2024), the documentary series *Farming during the War* (2023), and outcomes from the conference *The National Challenge: Soil Degradation or Restoration of Fertility?* (December 8, 2023). Agricultural operations have been severely hindered by attacks of the Russian army on agricultural infrastructure, including shelling, mining, burning of fields near active combat zones, a five-month blockade of Black Sea ports (resumed blockade in July 2023), and the destruction of the Kakhovka dam (Spears *et al.*, 2024).

Agricultural damages.
As of February 2023, Russia's invasion has caused $8.7 billion in direct losses and $31.5 billion in total damages to Ukraine’s agricultural sector (World Bank, 2023a). These include destruction of equipment, storage facilities, livestock and perennial crops, alongside looted resources. Unharvested crops, reduced production, increased costs and declining export prices for key commodities, such as wheat, barley, corn and sunflower seeds, further exacerbate the situation. The Kakhovka disaster alone inflicted $25 million in direct damages, leaving 600 thousand hectares without irrigation. About 30% of Ukraine’s farmland is currently not cultivated because this land is either under occupation or too dangerous to cultivate due to landmines, shell craters and contaminated soil.

An analysis (conducted in April 2023) of agricultural enterprises cultivating up to 250 hectares showed a 9% decrease in cereal crop area compared to the previous year, with frontline enterprises reporting a 20% reduction (FAO, 2023b). Additionally, 12% of enterprises indicated potential land contamination from unexploded ordnances, rising to 32% in frontline oblasts.

Preliminary findings from a December 2023 report (FAO, 2023a) on commercial crop producers revealed a 2.3% decrease in cultivated land in Ukraine, amounting to a loss of 266,400 ha, with 70% of the reduction in frontline oblasts. About 10% of enterprises reported potential contamination by unexploded ordnances, rising to 21% in frontline areas. Western oblasts saw a 25% increase in exports, while frontline areas experienced a 6% rise due to logistical issues. Storage capacity rose 10%, with nearly half receiving FAO support. Input disruptions affected 15% of enterprises, peaking at 19% in frontline oblasts. Market access and input availability (seeds, fertilizers, pesticides) were key concerns for most respondents.

Challenges.
Since the invasion, two major indirect issues hit the agricultural sector: (i) limited access to affordable financing and factor inputs, and (ii) low profitability due to export restrictions, particularly affecting small farmers. Logistical difficulties have paralyzed agribusinesses, with machinery, seeds and pesticides scattered across the country, while professional workers were relocated. About 65% of crop and 45% of livestock farmers (FAO, 2023) have faced shortages of fuel, seeds, feed, fertilizers and electric power, along with difficulties in selling their produce.

However, the rise in grain prices in 2024 and the partial recovery of fertilizer production have led to increased fertilizer purchases, with 30% of sales involving grain exchanges. Fertilizer prices per tonne in June 2024 were USD 465 for ammonium nitrate, USD 380 for calcium ammonium nitrate, USD 410–420 for UAN, and USD 540 for urea (Superagronom.com, 2024). These market prices are equivalent to around USD 1.32 per kg N.

A key challenge remains the exemption of male agricultural workers (ages 18–60) from military conscription. Agro-companies must meet at least three out of seven specific criteria (outlined below under the heading **’Law of Ukraine on Amendments Regarding Military Service, Mobilization and Military Records’)** to retain their workforce for a 12-month period.

Positive shifts amid the war.
Despite the dire situation, some farmers are adapting their practices. The aforementioned conference *National Challenge: Soil Degradation or Restoration of Fertility?* highlighted a shift toward more resilient farming methods (Latifundist.com, 2024). Cover crops are being used to enrich soil N, prevent winter erosion and increase biodiversity

by some agro-companies, though results vary due to regional droughts. Strip-till and no-till methods, biopreparations (*e.g.*, for pest control) and organic fertilizers are reported as being increasingly considered for use by some farmers (Latifundist.com, 2024). Some farms are establishing livestock operations and integrated crop – livestock systems. Nitrification inhibitors are reported as being tested by some agro-companies (so far on small plots), and there is a call for compost certification to support environmentally responsible farming (Latifundist.com, 2024).

Support programs for small farmers.
The Ministry of Agrarian Policy and Food of Ukraine, with financial support from the European Union's Production Grant Scheme, launched special support programs for small-scale farmers during the war. These include a payment of USD 84 per ha for farms up to 120 ha and a subsidy of USD 143 per head for farms with up to 100 cattle (KMU, 2022; tni.org, 2023). There are also grants for horticulture projects, supporting planting on up to 25 ha and constructing greenhouses up to 2 ha (tni.org, 2023). As of late 2023, more than 150 small farms have taken advantage of these programs, aimed at diversifying agricultural production by expanding horticulture and viticulture (World Bank, 2023b). The World Bank, under its Accelerating Private Investments in Agriculture initiative, allocated USD 132 million to bolster these efforts (World Bank, 2023b). The ARISE project will further increase financial support for around 90,000 small farms over the next two years, focusing on cultivating higher-value crops (World Bank, 2023b).

In its 2023 Humanitarian Response Programme for Ukraine, the Food and Agriculture Organization (FAO, 2023a) sought USD 205 million to support rural households and small-scale farmers in frontline oblasts by providing agricultural inputs. By 30 November 2023, USD 45.3 million was raised, leaving a USD 159.7 million gap. Since February 2022, FAO has aided over 185,000 rural families with seeds, animal feed and cash, and 10,000 farmers with grain storage, seeds, and generators (FAO, 2023a4a). For 2024, FAO indicates that it requires USD 151 million to assist 315,800 vulnerable families in securing crops, livestock health and restoring production (FAO, 2023a).

Furthermore, in its emergency grant aid for global food security, FAO (2024a) with Japan as a resource partner, contributed to USD 5 million to protect the food security and livelihoods in newly accessible war-affected areas in Ukraine, while laying the foundation for resilient agrifood systems. This grant supported 1,000 households in cultivating 12,000 ha, producing 40,000 tons of maize and 2,036 households in cultivating 50,900 ha, yielding 101,800 tons of sunflowers. This aid is reported by FAO (2024a) as also helping protect the food security, livelihoods and incomes of conflict-affected households amid rising production costs. FAO (2024a) concludes that this aid has helped sustain grain production, essential for domestic and international markets, particularly for countries reliant on Ukrainian grain.

New Agricultural Policy.
The presented draft of the Ukraine’s New Agricultural Policy (NAP, 2022), part of the national recovery plan, is divided into three phases. The first phase (2022) sought to maintain agricultural potential through food security measures, such as tax exemptions, simplified regulations and financial support. The second phase (2023-2025) aims to restore agricultural productivity and attract investment, particularly in infrastructure. It includes incentives for private investment, value added tax (VAT) reductions on food and measures to formalize agricultural production. For the third phase (2026-2032), the policy focuses on boosting agricultural efficiency, diversifying export risks and promoting sustainable land use through grain and oilseed processing and the development of the Danube River transport system.

However, the policy has faced criticism from farmer organizations and civil society for its emphasis on reviving the pre-war export-oriented model. Critics argue that the policy prioritizes production and export growth without addressing the needs of family farms or promoting sustainable, localized food systems, which proved crucial during the war (NASU, 2022; tni.org, 2023). The absence of programs supporting these small, more resilient family farming systems, along with the neglect of the Farm to Fork and Biodiversity principles of the EU’s Green Deal, raises concerns about the long-term sustainability of Ukraine’s agricultural recovery. The government’s challenge lies in balancing the restoration of export-oriented agriculture with the promotion of family farming, ensuring ecological, economic and social sustainability.

Within this new policy, we suggest that the establishment of regional advisory services to promote Good Agricultural Practices at the farm level is essential. These services would enhance farmers' understanding of nutrient management through education, tailored strategies and simplified cost-benefit analyses. Grants and loans for nutrient management investment would further drive change, fostering long-term sustainability. These advisors must also have access to the results of the national soil inventory and analysis campaigns, as well as to independent research data.

According to the **Law of Ukraine on Amendments Regarding Military Service, Mobilization and Military Records** (Ukraine, 2024), key criteria for a business to be classified as critical* for the national economy and security include (any 3 should be met to be eligible):

1. The total amount of taxes and payments made to state and local budgets (excluding customs duties) must exceed €1.5 million annually.
2. Annual foreign currency revenues (excluding loans) must exceed €32 million.
3. The business must be of strategic importance for the economy and state security, as per the government's list of strategic entities.
4. The business must play a vital role in the national economy or meet the needs of local communities. The criteria for defining such businesses are determined by executive authorities, relevant state bodies or regional and military administrations, depending on the sector or region involved.
5. No outstanding debts on social security contributions.
6. The average salary of insured employees must meet or exceed the regional average for the last quarter of 2021.
7. The business must be registered in 'Diia City'.

**For agricultural enterprises, critical status is granted by the Ministry of Agrarian Policy.*

After receiving critical status from the Ministry of Agrarian Policy, each agro-company (including self-employed farmers), regardless of its size, must meet at least three out of the seven specified criteria listed above to exempt their male agricultural workers (ages 18–60) from military conscription.

Note 3: Supplementary Methods

Note 3.1: Accounting for the data availability from the temporarily occupied areas of Ukraine

National statistical data in Ukraine are typically collected by administrative unit, following the hierarchy of *hromada* (community or municipality), *raion* (district of county) and *oblast* (county), on a monthly and quarterly (3-month) basis. The data are then collated, processed and submitted to the central office in Kyiv for final verification, analysis and distribution/publication (SSSU, 2025). In Ukraine, statistical data from the agricultural sector are typically publicly available at the national scale and, more recently (since the late 2000s), at the county scale (SSSU, 2025).

Note 3.2: Accounting for the total atmospheric deposition of N, P and K, including organic constituents

It is well shown that organic N may significantly contribute to the total N deposition, often by more than 30% (*e.g.*, Miyazaki *et al.*, 2010; Cape *et al.*, 2011; Cornell, 2011; Medinets *et al.*, 2012, 2020; Medinets, 2014; Li *et al.*, 2023). The highest organic fraction of the total has been reported by Medinets *et al.* (2020) on average at around 70% for monitoring sites (cropland, garden and natural wetland) located in Odesa region (Ukraine) over the period of 2011-2019. The authors assumed that the high ratios of organic fractions in N deposition observed in this study area might be attributed to several factors: (i) wind-induced and agri-management-induced soil dust formation from Black soils with high organic matter content (Medinets *et al.*, 2016), (ii) the influence of organic aerosols of marine origin due to proximity to the sea (Altieri *et al.*, 2016) and (iii) potentially region-specific biogeochemical conditions that favor the emission or atmospheric formation of organic compounds. Intensive agricultural practices (*e.g.*, fertilizer application, tillage) that generate additional soil dust containing urea, amines, macromolecules, and humic-like substances (McKenzie *et al.*, 2016) might explain the higher absolute content of total N, including organics, in samples collected from and near managed sites. However, it remains debatable how to disentangle primary deposition from re-deposition, as well as to determine whether the sources are locally originated or transferred from nearby areas, and which of these should be included. Moreover, it cannot be ruled out that microbial conversion from dissolved inorganic to particulate and organic N occurred within the total deposition samples during the fortnightly-monthly sampling period, despite the addition of biocide thymol to inhibit microbial growth. Nonetheless, further accurate investigations into organic N (and other nutrient depositions) are necessary in the near future to determine their contributions across different regions, enhance atmospheric models and incorporate these findings into balance calculations.

Based on available field data for southern Ukraine, mean annual total (organic and inorganic) atmospheric N, P and K deposition was 25.5±2.2 kg N ha^{-1} (with an estimated contribution of around 30% from dissolved inorganic N and the remainder presumably from organic N fractions), 2.5±0.6 kg P ha^{-1} (with a 56% contribution from dissolved inorganic P) and 3.1±1.2 kg K ha^{-1} (inorganic only) [values for Lower Dniester catchment, Odeska county, 2019–2021; Medinets *et al.*, 2020, 2024; Medinets V., pers. comm.]. For example, these values accounted for 21%, 24% and 21% of the mean total N, P and K fertilizer (119±6 kg N ha^{-1}, 10.5±0.5 kg P ha^{-1} and 14.8±0.3 kg K ha^{-1}; **Fig. 1**) applied to wheat over the same period, respectively. It is noteworthy that the mean rates presented above for dissolved inorganic P (1.4 kg P

ha^{-1}) are equal to the previously global mean for PO_4-P deposition reported by Tipping *et al.* (2014). Overall, these estimates, based on upscaling of measured deposition, demonstrate how atmospheric deposition of both P and K can be significant for areas receiving little or no input from fertilizers. Hence atmospheric deposition of both P and K needs to be considered alongside N (including the organic contributions) when calculating complete nutrient budgets.

Note 3.3: Accounting for crop residue management

Crop residues were mostly removed from the fields after harvest during the Soviet era, when collective farms (so-called kolkhozes) often consisted of both crop and livestock husbandry, and straw was necessary for livestock feeding and bedding (Buyanovskiy A., pers. comm.). From 1995 (in some regions from 1998), the livestock sector declined substantially (**Supplementary Fig. 6**), and crop residues were left or returned to the field, depending on the machinery used. Around the 2000s, most agro-enterprises (middle-scale >20 ha and <500 ha, and large-scale >500 ha) began leaving crop residues in the field, then either disking or mulching them. This was motivated by two reasons: (i) increasing the soil organic matter content and soil fertility, and (ii) a more pragmatic one - most of the new harvesting equipment came from Western countries and had straw chopper / spreaders and chaff spreaders. As a result, since 2010, a decrease of organic carbon loss in the soil has been observed (Bilanchyn *et al.*, 2021; Buyanovskiy *et al.*, 2021, 2022).

However, some enterprises continued to remove crop residues from the field after crop harvest because they were: (i) engaged in coupled animal-crop husbandry, or (ii) produced pellets to be used as an alternative energy source (Buyanovskiy A., pers. comm). In our nutrient budget calculations, we assumed that most crop residues were left in the field, and that the NPK in crop residues remained in the field system. However, it is worth noting that the humification rate of these crop residues as well as N loss during this process, varies substantially depending on their management (depth and types of ploughing, disking, mulching) and pedoclimatic parameters (temperature, precipitation, soil moisture content, soil type etc.).

Note 3.4: Accounting for crop rotation practice

In most cropping systems, a crop rotation scheme has been implemented, primarily to prevent soil-borne diseases (like root rot) and weed infestations. There is no standard crop rotation and each farmer/ agro-enterprise is guided by their own decision making. Crop rotations could include wheat - wheat - fallow, and wheat - sunflower - another crop, but there could be a total of 4 to 6 crop cultivations in a rotation cycle. However, there is also much evidence of monoculture cultivation of maize and wheat (*e.g.*, Pinchuk *et al.*, 2021; Mamonova *et al.*, 2023).

Ideally, a nutrient budget or balance should be calculated for a crop rotation cycle rather than on an annual basis for a certain crop, as an experienced agronomist is likely to estimate nutrient requirements considering what is left (crop residues, soil stock) from preceding crops. Data on such practices are not available, therefore for a statistical evaluation of the data we retain the comparisons at an annual level, which allows the overall trends to be seen most clearly. In this study, we focus on the general trends for the three main crops, while acknowledging that incorporating crop rotations may impact results at the site level.

Note 3.5: Limitations of the partial nutrient budget approach recommended by EUNEP

The EU Nitrogen Expert Panel (EUNEP, 2016) outlined the framework with the key metrics, such as N balance (surplus, deficit or equilibrium), N use efficiency (target range: 50-90%) and N yield (minimal desirable threshold: 80 kg N ha^{-1}) to optimize nutrient management across the agro-food sector to both increase yields and minimize nitrogen pollution. However, these above0mentioned EUNEP reference values are generalized, that is not crop-specific nor adjusted for regional conditions (Medinets and Sutton, 2025; Mi *et al.*, 2025). As real values may vary substantially depends on crop variety productivity, pedo-climatic conditions and management practices, the accurate region-tailored, crop-specific adjustment is needed based on field data/statistics. Mi *et al.* (2025) suggested region-dependent benchmarks for target N yield for wheat in China and the range of N surplus framing by unavoidable N losses (12-23 kg N ha-1) and farmer's economic optima (80-85 kg N ha-1). Whereas Medinets and Sutton (2025) recommended to set the upper limit of N surplus should not exceed twice the unavoidable N losses, estimated at about 10% of input, highlighting that future innovation should not see unavoidable losses as inevitable, but seek practices that reduce them further. Thus, due to absence of regional field study and/ or field-scale statistics and that in this study we analysed county mean data (as an input) without knowing standard error, we could not assess actual deviation in each county in the corresponding year for the studied crops to determine the threshold for desirable yields, we relied on the generalized EUNEP targets to

indicatively identify the productivity level for each county per year. Also, following the recommendations of Medinets and Sutton (2025), we set acceptable N surplus range between 10 and 20%, which has been incorporated into the Smart Fertilizer Planner tool.

The EU Nitrogen Expert Panel (EUNEP, 2016) proposed a framework with key metrics, such as N balance (surplus, deficit, or equilibrium), NUE (target range: 50–90%) and N yield (minimum desirable threshold: 80 kg N ha^{-1}) to optimize nutrient management across the agro-food sector, aiming to both increase yields and reduce N pollution. However, these reference values are generalized; they are neither crop-specific nor adjusted for regional conditions (Medinets and Sutton, 2025; Mi *et al.*, 2025). In practice, real values vary significantly depending on crop variety, productivity, pedo-climatic conditions and management practices. Therefore, accurate, region-specific, crop-adjusted benchmarks are needed, ideally based on field data/ statistics.

For example, Mi *et al.* (2025) proposed region-specific benchmarks for target N yields for wheat in China, alongside a range for N surplus bounded by unavoidable N losses (12–23 kg N ha^{-1}) and economic optima for farmers (80–85 kg N ha^{-1}). Medinets and Sutton (2025) further recommended that the upper limit for N surplus should not exceed twice the unavoidable losses, currently estimated at approximately 10% of inputs. They emphasized that future innovations should not treat these losses as inevitable but should instead pursue practices that minimize them.

Due to the absence of detailed regional field studies and field-scale statistics in Ukraine, and because our study used county-level mean data (without standard error estimates), we were unable to establish best practices based crop yields within each county for the studied crops in each year, or to assess actual yield deviations. As a result, we relied on the generalized EUNEP targets to indicatively assess productivity levels by county and year. Following Medinets and Sutton (2025), we also set an acceptable N surplus range of 10–20%, which has been incorporated into the *Smart Fertilizer Planner* tool.

Other uncertainties and limitations related to input sources, such as atmospheric N, P, K deposition rates, manure and other organic fertilizer characterization, crop residue management, as well as crop rotation practices are discussed in Methods and Supplementary Notes 3.2-3.4.

Supplementary Tables

Supplementary Table 1. Acronyms, full names of administrative units (*i.e.* counties or oblasts) of Ukraine and their reference to the corresponding geographical region of Ukraine. Supplementary Fig. 17 shows the locations of the counties and the geographical regions.

Code of county	Administrative unit (autonomic republic, county, special cities)	Geographical region of Ukraine
C-CK	Cherkaska	Central
C-CN	Chernihivska	Central
C-DN	Dnipropetrovska	Central
C-KI	Kirovohradska	Central
C-KY	Kyivska	Central
C-PO	Poltavska	Central
C-SU	Sumska	Central
C-VI	Vinnytska	Central
C-ZH	Zhytomyrska	Central
E-DO	Donetska	Eastern
E-KR	Kharkivska	Eastern
E-LU	Luhanska	Eastern
S-KH	Khersonska	Southern
S-MY	Mykolaivska	Southern
S-OD	Odeska	Southern
S-ZP	Zaporizka	Southern
W-CH	Chernivetska	Western
W-IF	Ivano-Frankivska	Western
W-KM	Khmelnytska	Western
W-LV	Lvivska	Western
W-RI	Rivnenska	Western
W-TE	Ternopilska	Western
W-VO	Volynska	Western
W-ZA	Zakarpatska	Western
S-ARC	Autonomic republic of Crimea (Krym)	Southern

Supplementary Table 2. Summary of phosphorus (P) inputs, P outputs, P balances and P use efficiencies (PUE) for wheat, maize and sunflower under five nutrient management projections by 2030 alongside cumulative P balances over 2024–2030 for business-as-usual (BaU, S-0) and extended war disruption (S-w) scenarios. S-1 stands for the manure-enriched precision fertilization scenario; S-2 stands for the enhanced-efficiency fertilizers scenario; S-3 stands for the legume-based diversification scenario. 'Total P input' includes synthetic P fertilizer and organic (manure) P fertilizer (see Methods). 'Org.' represents manure-P; 'Synth.' represents synthetic P fertilizer; 'Crop available' stands for P available for crop uptake within year of application (and previous-year legacy from manure-P; see Supplementary Note 2.8 for details). 'Mt P' represents million tonnes of P. Scenario descriptions in Supplementary Note 2.8.

**Crop**	**Scenario**	**P input, kg P ha^{-1} yr^{-1}**					**P output, kg P ha^{-1} yr^{-1}**	**P balance, kg P ha^{-1} yr^{-1}**		**P surplus/deficit, %**		**PUE, %**		**Cumulative balance by 2030, Mt P**
		**Total**	**Total org.**	Crop-available				**Total**	Crop-available	**Total**	Crop-available	**Total**	Crop-available	
				Org.	**Synth.**	Total								
**Wheat**	**S-0**	**11.2**	**0.7**	0.48	**10.5**	11.01	17.23	**-6.0**	-6.2	**-53.6**	-56.5	**154**	157	**-0.04**
	**S-1**	**20.9**	**10.3**	7.23	**10.5**	17.76	17.23	**3.6**	0.5	**17.4**	3.0	**82.6**	97.0	-
	**S-2**	**20.9**	**10.3**	7.23	**10.5**	17.76	17.23	**3.6**	0.5	**17.4**	3.0	**82.6**	97.0	-
	**S-3***	**20.9**	**10.3**	7.23	**10.5**	17.76	17.23	**3.6**	0.5	**17.4**	3.0	**82.6**	97.0	-
	**S-w**	**4.1**	**0.5**	0.32	**3.64**	3.96	17.23	**-13.1**	-13.3	**-320**	-335	**420**	435	**-1.5**
**Maize**	**S-0**	**12.5**	**1.6**	1.15	**10.9**	12.05	22.28	**-9.8**	-10.2	**-78.2**	-84.9	**178**	185	**-0.07**
	**S-1**	**25.3**	**10.0**	7.02	**15.3**	22.28	22.28	**3.0**	0	**11.9**	0	**88.1**	100	-
	**S-2**	**25.3**	**10.0**	7.02	**15.3**	22.28	22.28	**3.0**	0	**11.9**	0	**88.1**	100	-
	**S-3***	**25.3**	**10.0**	7.02	**15.3**	22.28	22.28	**3.0**	0	**11.9**	0	**88.1**	100	-
	**S-w**	**7.5**	**0.9**	0.63	**6.5**	7.13	22.28	**-14.8**	-15.2	**-197**	-213	**297**	313	**-0.8**
**Sunflower**	**S-0**	**13.1**	**0.8**	0.59	**12.3**	12.89	10.10	**3.0**	2.8	**22.9**	21.6	**77.1**	78.4	**0.02**
	**S-1**	**11.6**	**5.1**	3.57	**6.5**	10.10	10.10	**1.5**	0	**13.2**	0	**86.8**	100	-
	**S-2**	**11.6**	**5.1**	3.57	**6.5**	10.10	10.10	**1.5**	0	**13.2**	0	**86.8**	100	-
	**S-3***	**11.6**	**5.1**	3.57	**6.5**	10.10	10.10	**1.5**	0	**13.2**	0	**86.8**	100	-
	**S-w**	**5.0**	**0.4**	0.31	**4.6**	4.91	10.10	**-5.1**	-5.2	**-102**	-106	**202**	206	**-0.5**

*S-3 is a qualitative scenario with indicative values, building on S-2. It assumes multilateral benefits for subsequent crops and soil health within the crop rotation, although these benefits are not quantified.

Supplementary Table 3. Summary of potassium (K) inputs, K outputs, K balances and K use efficiencies (KUE) for wheat, maize and sunflower under five nutrient management projections by 2030 alongside cumulative K balances over 2024–2030 for business-as-usual (BaU, S-0) and extended war disruption (S-w) scenarios. S-1 stands for the manure-enriched precision fertilization scenario; S-2 stands for the enhanced-efficiency fertilizers scenario; S-3 stands for the legume-based diversification scenario. 'Total K input' includes synthetic K fertilizer and organic (manure) K fertilizer (see Methods). 'Org.' represents manure-K; 'Synth.' represents synthetic K fertilizer; 'Crop available' stands for K available for crop uptake within year of application (and previous-year legacy from manure-K; see Supplementary Note 2.8 for details). 'Mt K' represents million tonnes of K. Scenario descriptions in Supplementary Note 2.8.

**Crop**	**Scenario**	**K input, kg K ha^{-1} yr^{-1}**					**K output, kg K ha^{-1} yr^{-1}**	**K balance, kg K ha^{-1} yr^{-1}**		**K surplus/deficit, %**		**KUE, %**		**Cumulative balance by 2030, Mt K**
		**Total**	**Total org.**	Crop-available				**Total**	Crop-available	**Total**	Crop-available	**Total**	Crop-available	
				Org.	**Synth.**	Total								
**Wheat**	**S-0**	15.2	1.8	1.4	13.5	14.9	20.9	-5.6	-6.0	-36.9	-40.1	137	140	**-0.04**
	**S-1**	26.4	26.4	21.1	0.0	21.1	20.9	5.5	0.2	20.9	1.1	79.1	98.9	-
	**S-2**	26.4	26.4	21.1	0.0	21.1	20.9	5.5	0.2	20.9	1.1	79.1	98.9	-
	**S-3***	26.4	26.4	21.1	0.0	21.1	20.9	5.5	0.2	20.9	1.1	79.1	98.9	-
	**S-w**	6.6	1.3	1.0	5.3	6.3	20.9	-14.3	-14.6	-219	-231	319	331	**-1.0**
**Maize**	**S-0**	22.4	2.4	1.9	18.2	20.1	30.0	-7.6	-9.8	-33.8	-48.9	134	149	**-0.05**
	**S-1**	35.1	25.6	20.5	9.5	30.0	30.0	5.1	0.0	14.6	0	85.4	100	-
	**S-2**	35.1	25.6	20.5	9.5	30.0	30.0	5.1	0.0	14.6	0	85.4	100	-
	**S-3***	35.1	25.6	20.5	9.5	30.0	30.0	5.1	0.0	14.6	0	85.4	100	-
	**S-w**	14.2	2.5	2.0	11.7	13.7	30.4	-16.2	-16.7	-114	-122	214	222	**-0.5**
**Sunflower**	**S-0**	18.7	2.2	1.7	16.5	18.2	17.6	1.1	0.6	5.8	3.2	94.2	96.8	**0.01**
	**S-1**	20.2	13.0	10.4	7.2	17.6	17.6	2.6	0.0	12.9	0.0	87.1	100	-
	**S-2**	20.2	13.0	10.4	7.2	17.6	17.6	2.6	0.0	12.9	0.0	87.1	100	-
	**S-3***	20.2	13.0	10.4	7.2	17.6	17.6	2.6	0.0	12.9	0.0	87.1	100	-
	**S-w**	8.4	1.2	1.0	7.2	8.2	17.6	-9.2	-9.5	-110	-116	210	216	**-0.6**

*S-3 is a qualitative scenario with indicative values, building on S-2. It assumes multilateral benefits for subsequent crops and soil health within the crop rotation, although these benefits are not quantified.

Supplementary Table 4. Comparison of average N, P and K content in different types of cattle and poultry manure derived from various literature sources. The coefficients used in this study are marked with * and highlighted in green; these were used to calculate the annual weighted coefficients for 'undefined' manure, based on the annual shares of poultry and cattle manure applied to the field (see **Methods; Supplementary Table 5**).

**Type**	**Source**	**Manure type**	**Average nutrient content, %**					**Reference**
			**N**	**P_2O_5**	**P**	**K_2O**	**K**	
**Cattle**	Ukrainian monograph	Cattle manure liquid (fresh)	**0.40**	0.20	**0.09**	0.40	**0.33**	Pisarenko and Pisarenko, 2022
		Cattle manure liquid (semi-rotted / rotted)	**0.90**	0.30	**0.13**	0.70	**0.58**	
		Cattle manure on straw bedding (fresh)*	**0.52**	0.31	**0.14**	0.60	**0.50**	
		Cattle manure on straw bedding (semi-rotted)	**0.60**	0.38	**0.17**	n/a	**n/a**	
		Cattle manure on straw bedding (rotted)	**0.66**	0.43	**0.19**	n/a	**n/a**	
		Cattle manure on straw bedding (compost)	**0.73**	0.48	**0.21**	n/a	**n/a**	
	Belorussian Journal	Cattle manure without bedding	**0.25**	0.15	**0.07**	0.35	**0.29**	Seraya *et al.*, 2024
		Cattle manure on straw bedding	**0.50**	0.30	**0.13**	0.60	**0.50**	
	Ukrainian agronomic portal	Manure on average	**0.50-0.53**	0.25-0.30	**0.11-0.13**	0.5-0.6	**0.42-0.5**	Superagronom, 2017
	Ontario Ministry of Agriculture and Food	Avg beef manure (liquid)	**0.37**		**0.08**		**0.23**	Brown, 2013
		Avg beef manure (solid)	**0.92**		**0.33**		**0.66**	
		Avg dairy manure (liquid)	**0.39**		**0.09**		**0.25**	
		Avg dairy manure (solid)	**0.72**		**0.20**		**0.61**	
	USDA report	Beef cow manure (liquid)	**0.24**	0.19	**0.08**	0.29	**0.24**	Lorimor *et al.*, 2004; Teng *et al.*, 2023
		Beef cow manure (solid)	**0.32**	0.18	**0.08**	0.32	**0.26**	
		Dairy cow manure (liquid)	**0.37**	0.18	**0.08**	0.23	**0.19**	
		Dairy cow manure (solid)	**0.45**	0.14	**0.06**	0.27	**0.23**	
**Poultry**	Ukrainian monograph	Poultry manure (chicken)*	**1.63**	1.60	**0.70**	0.85	**0.71**	Pisarenko and Pisarenko, 2022
		Poultry manure (duck)	**1.00**	1.40	**0.61**	0.54	**0.45**	
		Poultry manure (goose)	**0.55**	0.62	**0.27**	0.95	**0.79**	
	Ontario Ministry of Agriculture and Food	Avg poultry manure (liquid)	**0.81**		**0.28**		**0.30**	Brown, 2013
		Avg poultry manure (solid)	**2.71**		**1.32**		**1.45**	
	USDA report	Broiler manure (liquid)	**0.76**	0.48	**0.21**	0.35	**0.29**	Lorimor *et al.*, 2004; Teng *et al.*, 2023
		Broiler manure (solid)	**2.09**	2.41	**1.05**	1.63	**1.36**	

Supplementary Table 5. Estimated weighted content of N, P and K in 'undefined' manure reported by SSSU (2024). The estimates were calculated annually, based on the proportions of poultry manure on one hand, and cattle, other manure and organics on the other, within the organic fertilizer applied to fields. Data marked with * were reported in the national statistics (SSSU, 2025); other values were estimated (see **Methods** for details).

Year	Share in applied organic fertilizer		Content in cattle manure			Content in poultry manure			Weighted content in 'undefined' manure		
	Cattle, other manure and organics, %	Poultry*, %	N, %	P, %	K, %	N, %	P, %	K, %	N, %	P, %	K, %
1980	98.33	1.80	0.52	0.14	0.50	1.63	0.70	0.71	**0.54**	**0.15**	**0.50**
1986	98.24	1.90	0.52	0.14	0.50	1.63	0.70	0.71	**0.54**	**0.15**	**0.50**
1987	98.21	1.93	0.52	0.14	0.50	1.63	0.70	0.71	**0.54**	**0.15**	**0.50**
1990	98.18	1.96	0.52	0.14	0.50	1.63	0.70	0.71	**0.54**	**0.15**	**0.50**
2000	97.90	2.26	0.52	0.14	0.50	1.63	0.70	0.71	**0.55**	**0.15**	**0.51**
2001	97.63	2.53	0.52	0.14	0.50	1.63	0.70	0.71	**0.55**	**0.15**	**0.51**
2002	97.41	2.77	0.52	0.14	0.50	1.63	0.70	0.71	**0.55**	**0.15**	**0.51**
2003	97.16	3.03	0.52	0.14	0.50	1.63	0.70	0.71	**0.55**	**0.15**	**0.51**
2004	96.74	3.47	0.52	0.14	0.50	1.63	0.70	0.71	**0.56**	**0.15**	**0.51**
2005	96.08	4.14	0.52	0.14	0.50	1.63	0.70	0.71	**0.57**	**0.16**	**0.51**
2006	95.68	4.55	0.52	0.14	0.50	1.63	0.70	0.71	**0.57**	**0.16**	**0.51**
2007	95.45	4.79	0.52	0.14	0.50	1.63	0.70	0.71	**0.57**	**0.16**	**0.51**
2008	94.79	5.45	0.52	0.14	0.50	1.63	0.70	0.71	**0.58**	**0.17**	**0.51**
2009	94.10	6.14	0.52	0.14	0.50	1.63	0.70	0.71	**0.59**	**0.17**	**0.51**
2010	93.55	6.68	0.52	0.14	0.50	1.63	0.70	0.71	**0.60**	**0.17**	**0.51**
2011	92.80	7.41	0.52	0.14	0.50	1.63	0.70	0.71	**0.60**	**0.18**	**0.52**
2012	92.71	7.50	0.52	0.14	0.50	1.63	0.70	0.71	**0.60**	**0.18**	**0.52**
2013	92.56	7.65	0.52	0.14	0.50	1.63	0.70	0.71	**0.61**	**0.18**	**0.52**
2014	91.91	8.27	0.52	0.14	0.50	1.63	0.70	0.71	**0.61**	**0.18**	**0.52**
2015	91.39	8.76	0.52	0.14	0.50	1.63	0.70	0.71	**0.62**	**0.18**	**0.52**
2016	91.47	8.69	0.52	0.14	0.50	1.63	0.70	0.71	**0.62**	**0.18**	**0.52**
2017	91.33	8.82	0.52	0.14	0.50	1.63	0.70	0.71	**0.62**	**0.19**	**0.52**
2018	87.80*	12.20*	0.52	0.14	0.50	1.63	0.70	0.71	**0.66**	**0.20**	**0.53**
2019	90.52*	9.48*	0.52	0.14	0.50	1.63	0.70	0.71	**0.63**	**0.19**	**0.52**
2020	88.66*	11.34*	0.52	0.14	0.50	1.63	0.70	0.71	**0.65**	**0.20**	**0.52**
2021	87.48*	12.52*	0.52	0.14	0.50	1.63	0.70	0.71	**0.66**	**0.21**	**0.53**
2022	90.07*	9.93*	0.52	0.14	0.50	1.63	0.70	0.71	**0.63**	**0.19**	**0.52**
2023	90.96*	9.04*	0.52	0.14	0.50	1.63	0.70	0.71	**0.62**	**0.19**	**0.52**

Supplementary Table 6. Comparison of crop removal coefficients (or nutrient content) for wheat, sunflower and maize crop derived from various databases and field studies. In this paper we used the recent global average crop removal coefficients derived from the meta-analysis conducted by Ludemann *et al.* (2023, 2024).

Yield	Dataset name	Crop removal coefficients; N, P and K contents in harvested yield (dry matter)			Reference
		g N kg^{-1}	g P kg^{-1}	g K kg^{-1}	
Wheat	Global average	20.7	3.8	4.6	N: Ludemann *et al.*, 2023, 2024<br>P: Ludemann *et al.*, 2023, 2024<br>K: Ludemann *et al.*, 2023, 2024
	Mixed dataset: UNECE GD on national nitrogen budget, Grain Research & Development Corporation (GRDC), EC Knowledge4policy - Nutritional value of whole grains	18.1	3.1	3.6	N: ECE/EB.AIR/119, 2013<br>P: GRDC, 2019<br>K: Knowledge4policy, 2021
	The International Plant Nutrition Institute (IPNI) Canada	19.0	3.5	4.0	N: IPNI, 2014<br>P: IPNI, 2014<br>K: IPNI, 2014
	USDA Nutritional data	24.1	3.5	3.8	N: USDA, 2020<br>P: USDA, 2020<br>K: USDA, 2020
	Long-term Ukrainian field data (Odesa county)	20.8±0.4			N: Buyanovskiy A (pers. comm.)
Sunflower	Global average	25.6	4.1	7.1	N: Ludemann *et al.*, 2023, 2024<br>P: Ludemann *et al.*, 2023, 2024<br>K: Ludemann *et al.*, 2023, 2024
	Mixed dataset: UNECE GD on national nitrogen budget & the study of Li *et al.* (2018)	39.7	8.1	4.8	N: ECE/EB.AIR/119, 2013<br>P: Li *et al.*, 2018<br>K: Li *et al.*, 2018
	The International Plant Nutrition Institute (IPNI) Canada	27.0	4.2	7.5	N: IPNI, 2014<br>P: IPNI, 2014<br>K: IPNI, 2014
	USDA Nutritional data	35.6	7.3	6.6	N: USDA, 2020<br>P: USDA, 2020<br>K: USDA, 2020
	Long-term Ukrainian field data (Odesa State Agricultural Experimental Station, Odesa county)	27.1±0.9			N: Buyanovskiy A. (pers. comm.)
Maize	Global average	12.3	2.9	3.9	N: Ludemann *et al.*, 2023, 2024<br>P: Ludemann *et al.*, 2023, 2024<br>K: Ludemann *et al.*, 2023, 2024
	Mixed dataset: UNECE GD on national nitrogen budget & NutritionalValue.org	20.0	2.1	2.9	N: ECE/EB.AIR/119, 2013<br>P: NutritionalValue, 2024<br>K: NutritionalValue, 2024
	The International Plant Nutrition Institute (IPNI) Canada	12.0	2.8	3.7-5.3	N: IPNI, 2014<br>P: IPNI, 2014<br>K: IPNI, 2014
	USDA Nutritional data	n/a	0.9	2.7	N: USDA, 2020<br>P: USDA, 2020<br>K: USDA, 2020
	Long-term Ukrainian field data (Odesa county)	17.0±1.0			N: Buyanovskiy A. (pers. comm.)

Supplementary Figures

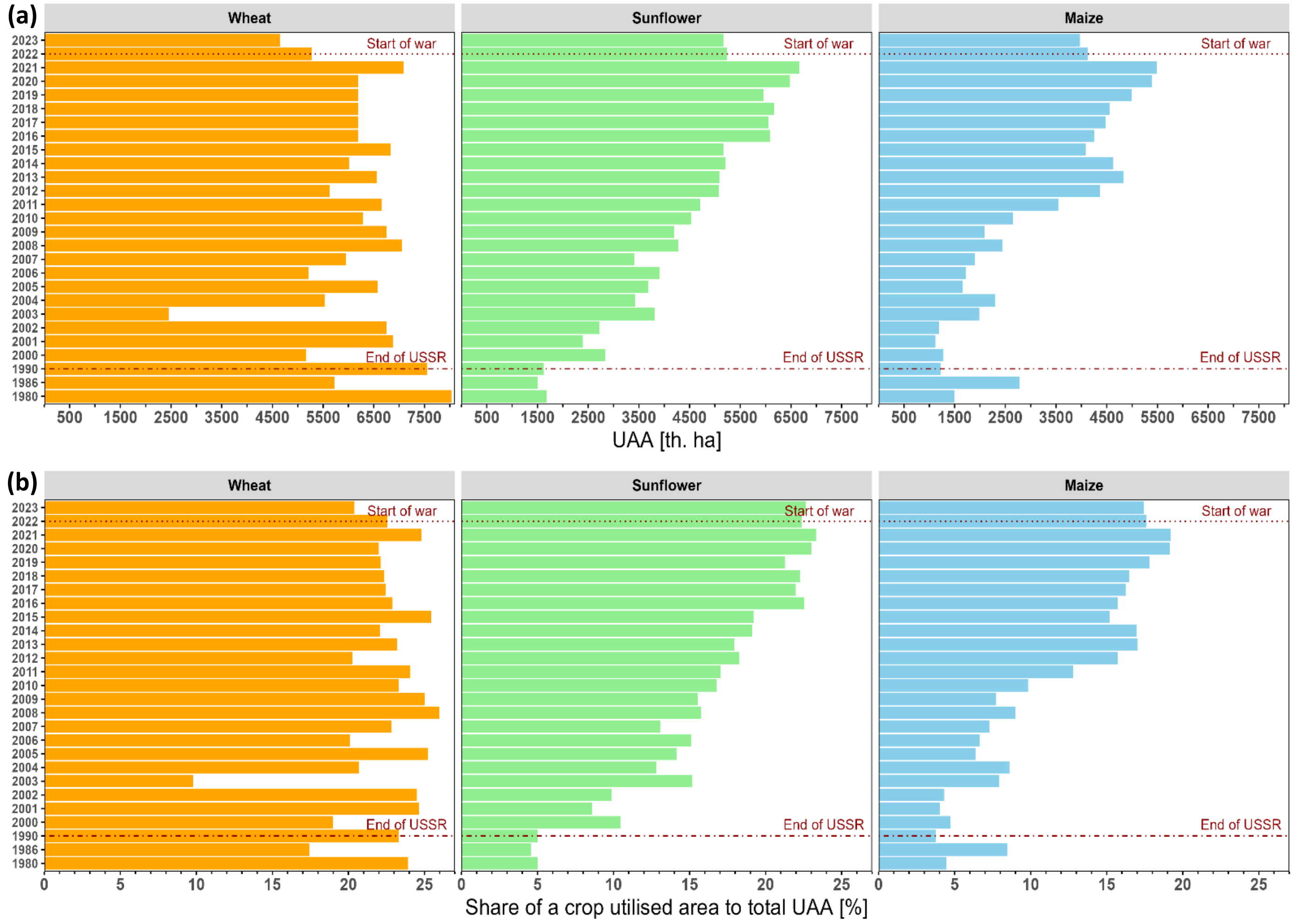

Supplementary Figure 1. Utilized agricultural areas (UAA) allocated to wheat, sunflower and maize cultivation (a) and their respective shares of Ukraine's total UAA (b) for the years 1980, 1986, 1990 and 2000-2023 (data source: SSSU, 2025). The unit th. ha represents thousand hectares. Dotted lines in 2022 show the start of the large-scale war; dash-dotted lines in 1990 show the end of the USSR. The total UAA refers to the total area of land used for agricultural activities, including arable land, permanent crops and permanent grassland or pasture.

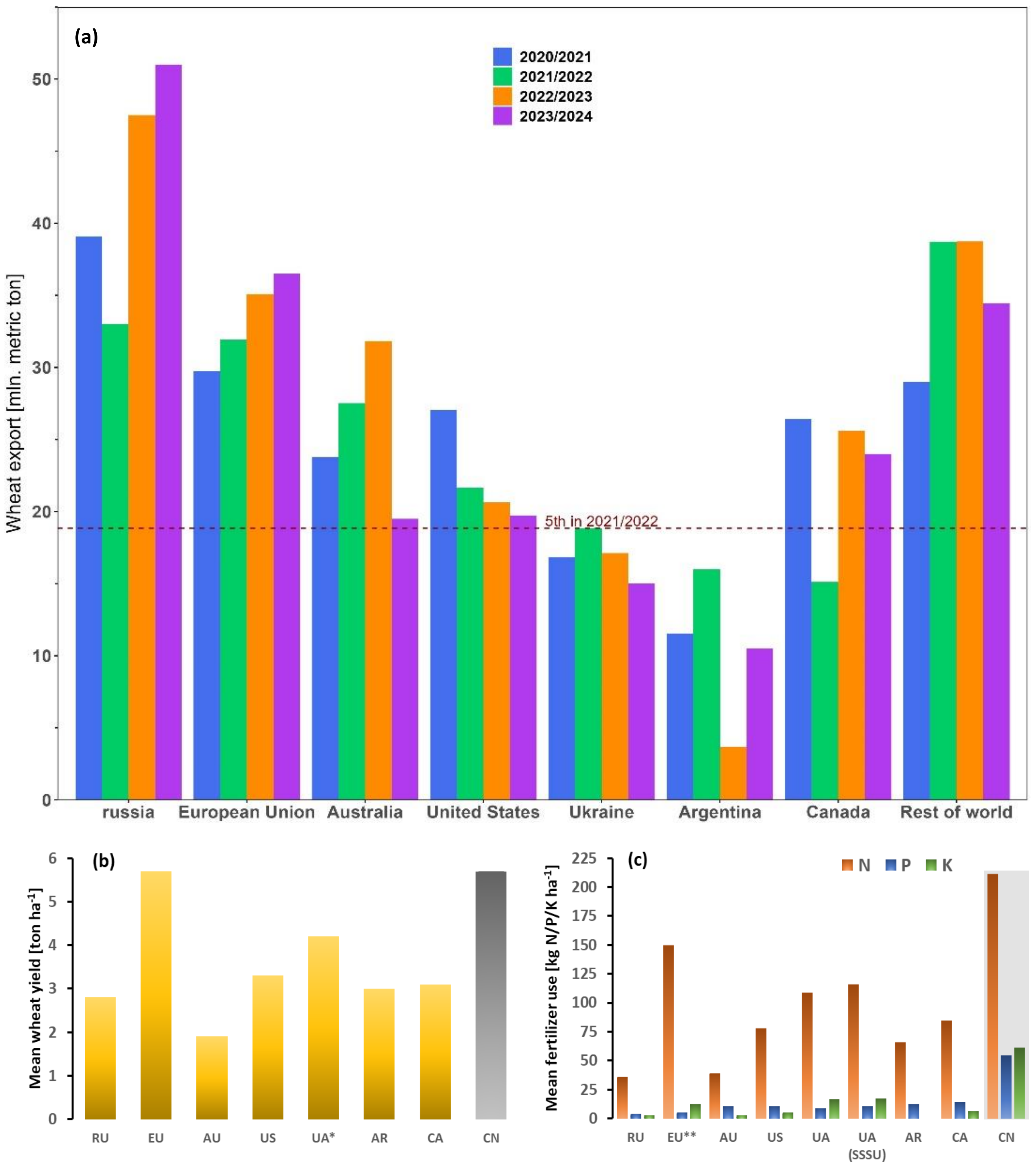

Supplementary Figure 2. Wheat exports (a) from the main exporting countries during 2020 to 2024 (data source: USDA, 2024), average wheat yields (b) in the main exporting countries and China for 2019-2021 (data source: FAO, 2024) and average synthetic fertilizer application for wheat crop (c) in the main exporting countries and China in 2018 (data source: Ludemann *et al.*, 2022). China (grey highlighted) is not one of the key exporting countries for wheat and shown for comparison only. The dashed line shows the Ukraine's rank in 2021/2022. UA*: the mean yield data for Ukraine derived from FAO (2024) and the State Statistics Service of Ukraine (SSSU, 2025) were equal; EU**: as EU-weighted data for fertilizer application to wheat crop was not available, therefore we used the mean fertilizer use in wheat crop from Germany as a representative; UA (SSSU): the mean synthetic fertilizer derived from the national statistics (SSSU, 2025) and used in this study, which slightly differed from those reported by the International Fertilizer Association (Ludemann *et al.*, 2022); RU: Russia, EU: the European Union; Au: Australia; US: the United States; UA: Ukraine; AR: Argentina; CA: Canada; CN: China.

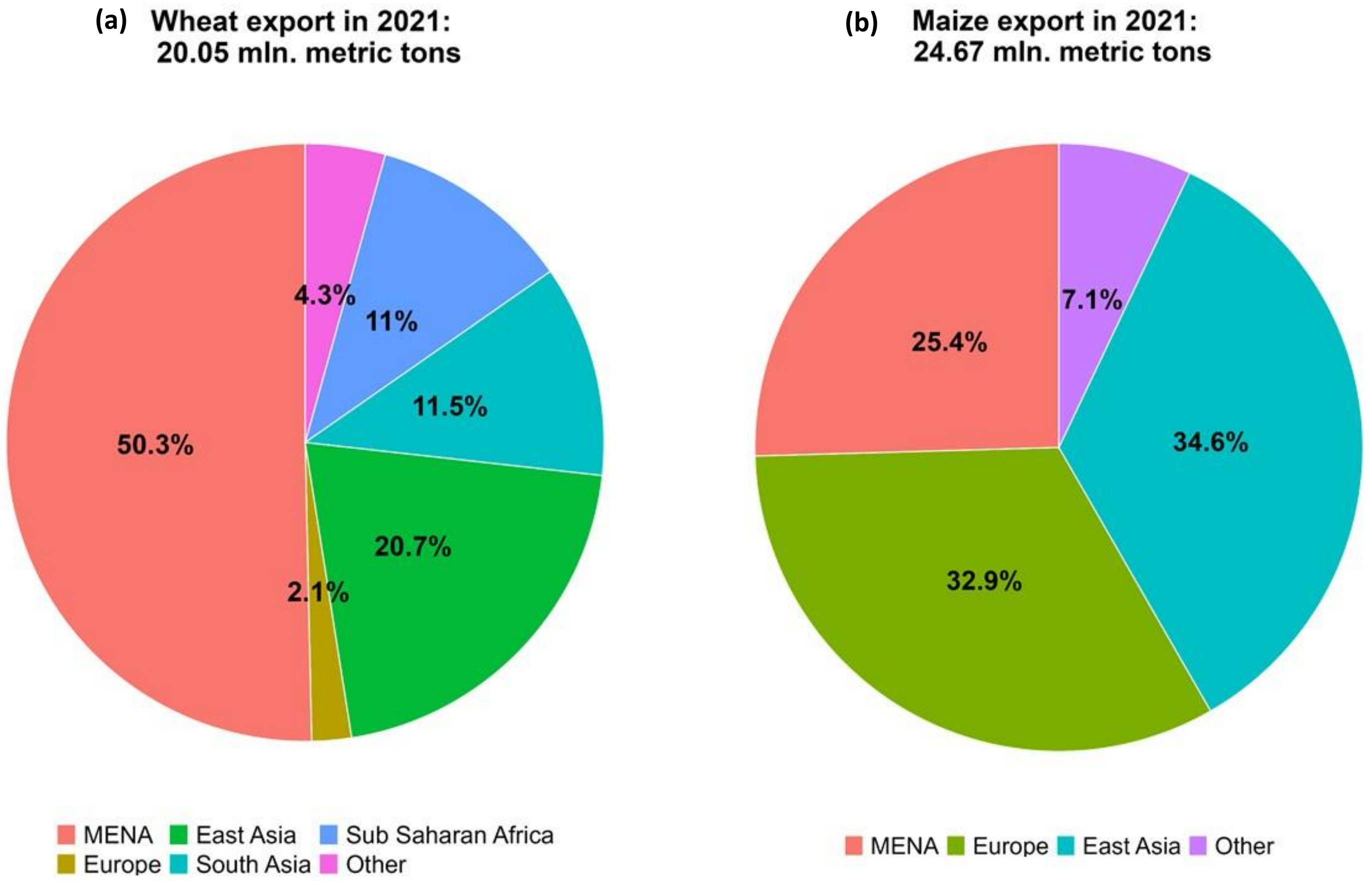

Supplementary Figure 3. Wheat (a) and maize (b) exports from Ukraine to global regions in 2021 (adapted from Glauber, 2024). MENA refers to countries in the Middle East and North Africa; Türkiye is included in MENA in this analysis.

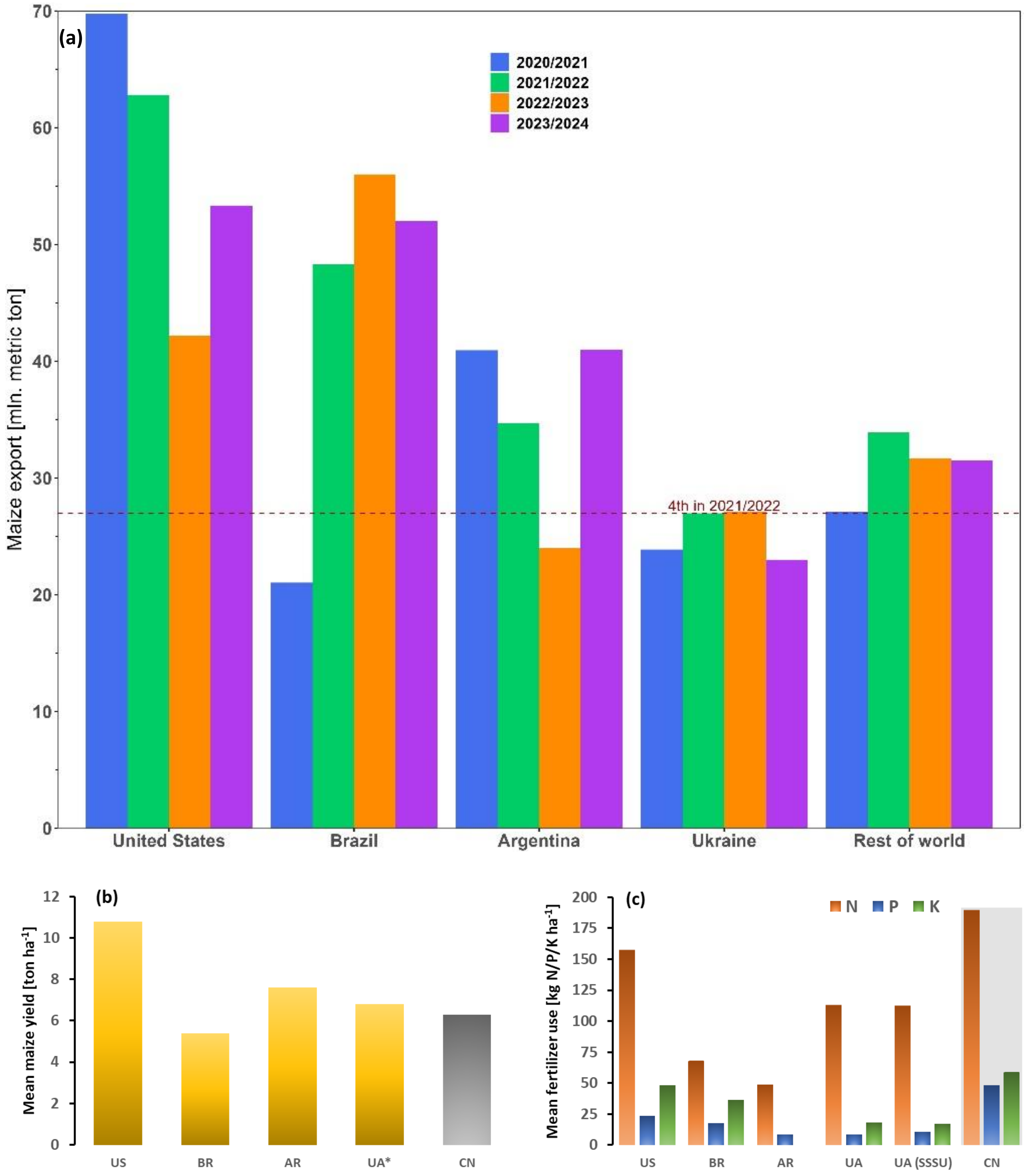

Supplementary Figure 4. Maize exports (a) from the main exporting countries during 2020 to 2024 (data source: USDA, 2024), average maize yields (b) in the main exporting countries for 2019-2021 (data source: FAO, 2024) and average synthetic fertilizer application for maize crop (c) in the main exporting countries in 2018 (data source: Ludemann *et al.*, 2022). China (grey-highlighted) is not one of the key exporting countries for maize and shown for comparison only. The dashed line shows the Ukraine's rank in 2021/2022. UA*: the mean yield data for Ukraine derived from FAO (2024) and Ukrainian national statistics (SSSU, 2025) are equal; UA (SSSU): the mean synthetic fertilizer derived from the national statistics (SSSU, 2025) and used in this study, which slightly differed from those reported by the International Fertilizer Association (Ludemann *et al.*, 2022); US: the United States; BR: Brazil; AR: Argentina; UA: Ukraine; CN: China.

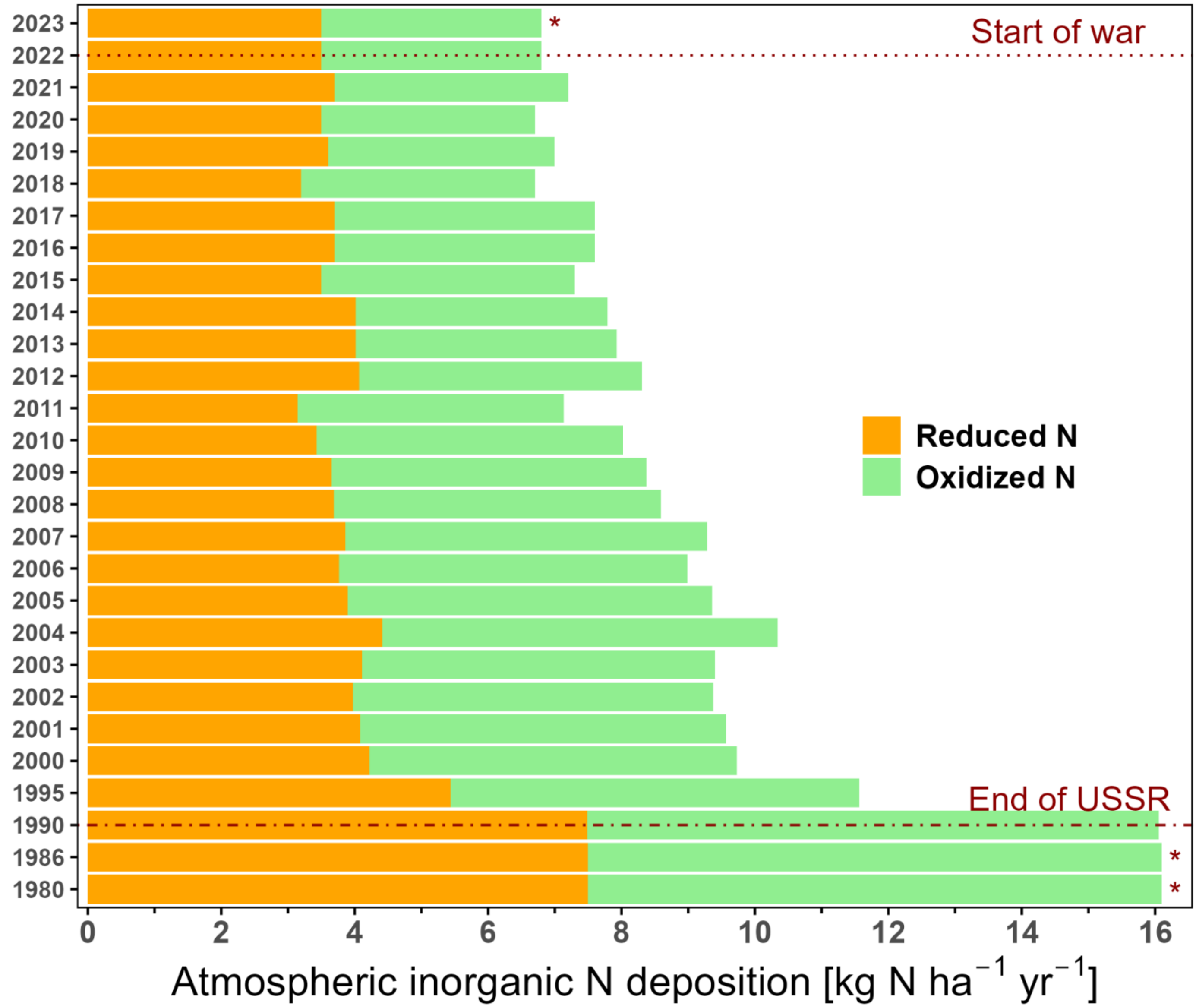

Supplementary Figure 5. Mean annual deposition (kg N ha^{-1} yr^{-1}) of reduced inorganic nitrogen, oxidized inorganic nitrogen and the total inorganic nitrogen in Ukraine for 1980, 1986, 1990, 1995 and 2000-2023. The original dataset covered the period 1990–2022 was modelled by the EMEP-MSC-W model (adapted from Klein *et al.*, 2024); *annual deposition values for 1980 & 1986, as well as 2023 (which were needed for the N balance calculation but absent from the dataset), were roughly assumed to be similar to those of 1990 and 2022, respectively. Dotted lines in 2022 show the start of the large-scale war; dash-dotted lines in 1990 show the end of the USSR.

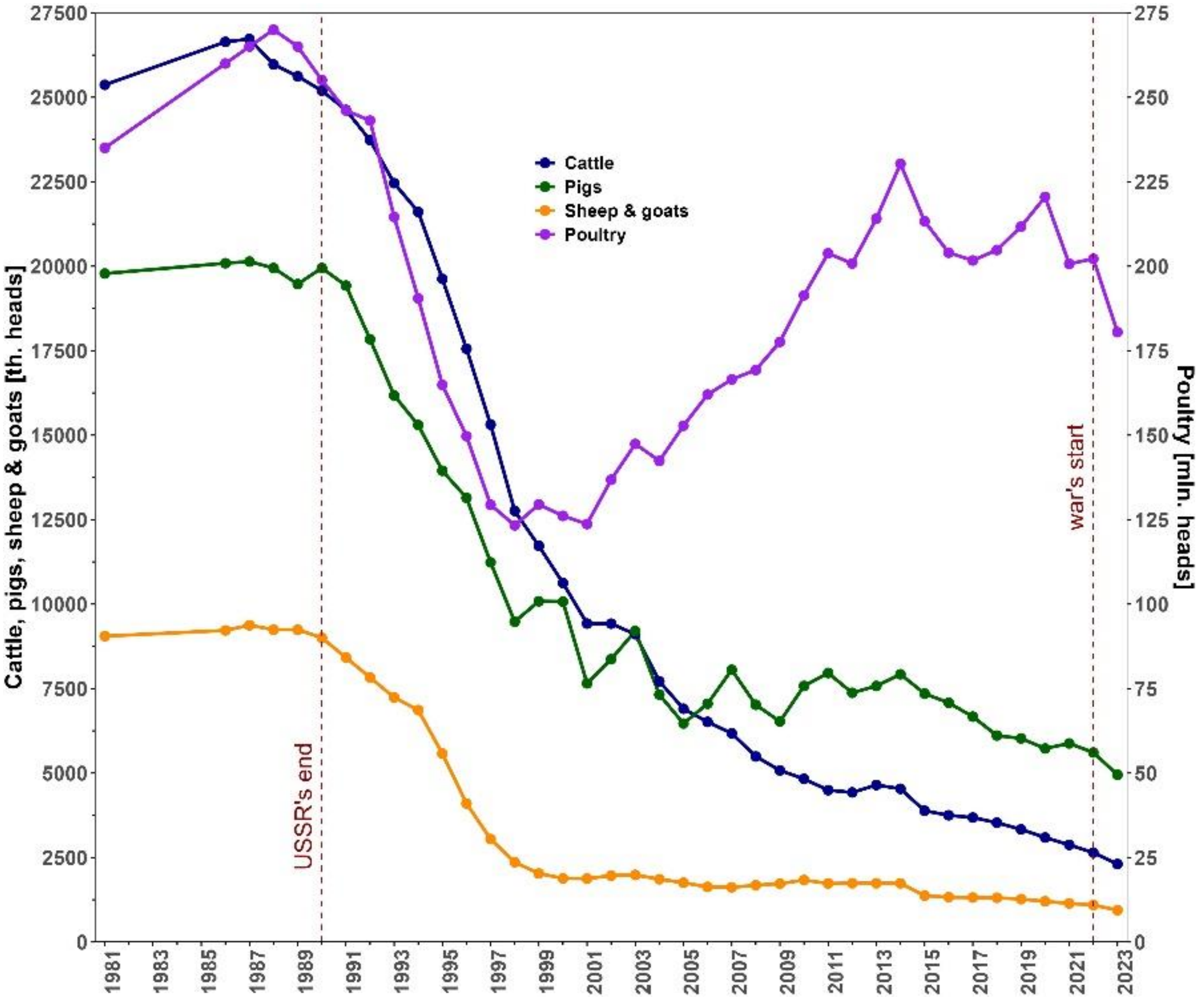

Supplementary Figure 6. Dynamics of agricultural animal numbers (cattle, pigs, sheep and goats, and poultry) in Ukraine from 1981 to 2023 (data source: SSSU, 2025). Note: left and right axes have different units for improved readability.

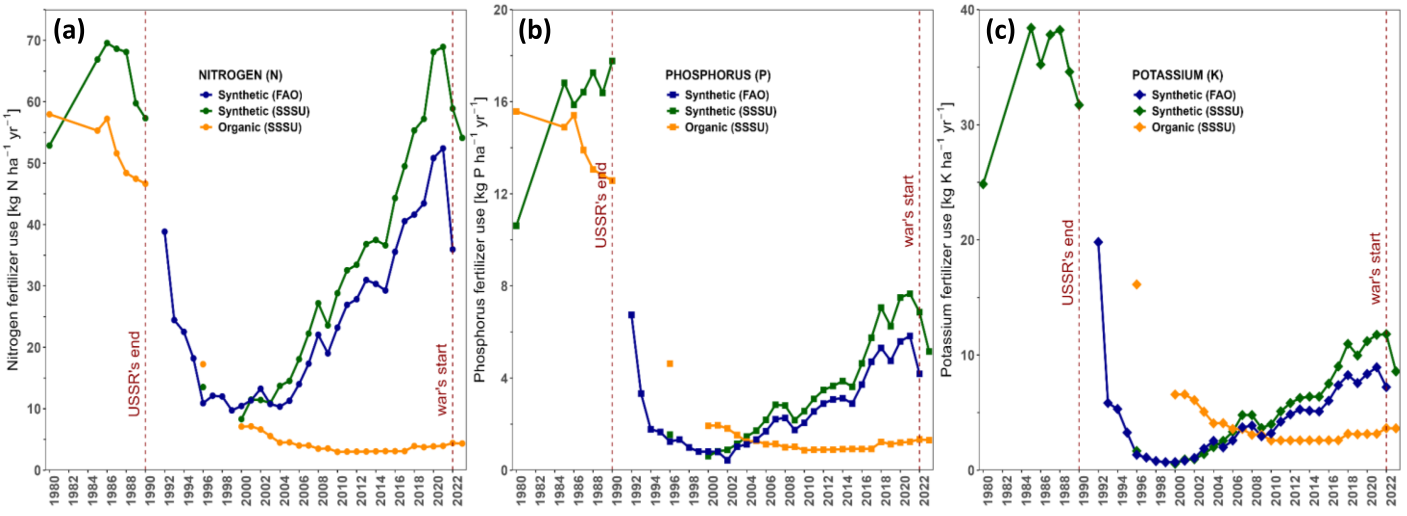

Supplementary Figure 7. Average annual N (a), P (b) and K (c) fertilizer application (kg N/P/K ha^{-1} yr^{-1}) in agriculture in Ukraine from 1980 to 2023. Dashed lines represent the final year of USSR era and the beginning of the large-scale war in Ukraine, respectively. SSSU means data derived from the State Statistics Service of Ukraine (SSSU, 2025); FAO means data taken from FAO statistics service (FAO, 2024).

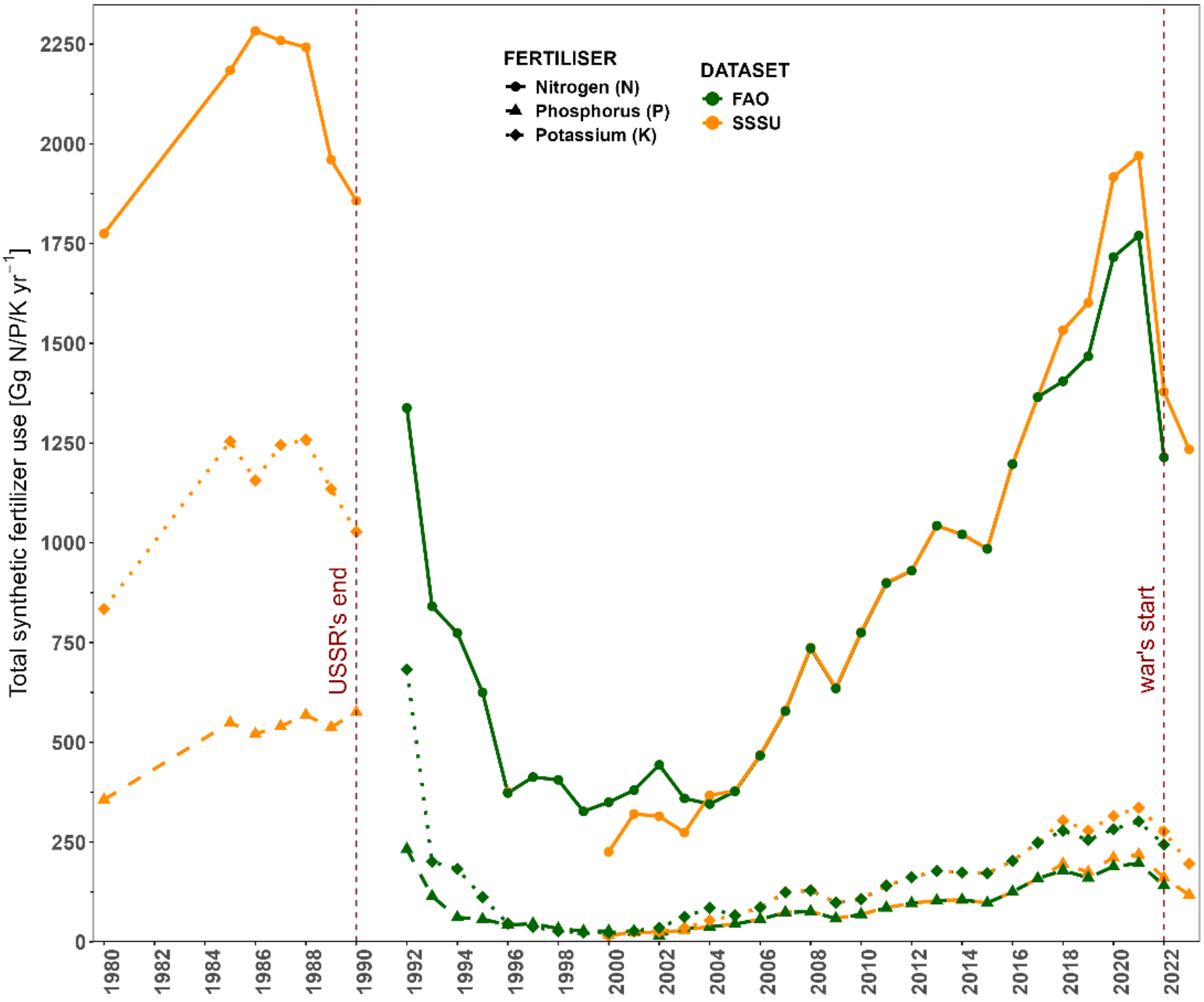

Supplementary Figure 8. Total annual synthetic N, P and K fertilizer application (Gg N/P/K yr^{-1}) in agriculture in Ukraine from 1980 to 2023. Dashed lines represent the final year of USSR era and the beginning of the large-scale war in Ukraine. SSSU means data derived from the State Statistics Service of Ukraine (SSSU, 2025); FAO means data taken from FAO statistics service (FAO, 2024). One Gg equals one thousand metric tonnes.

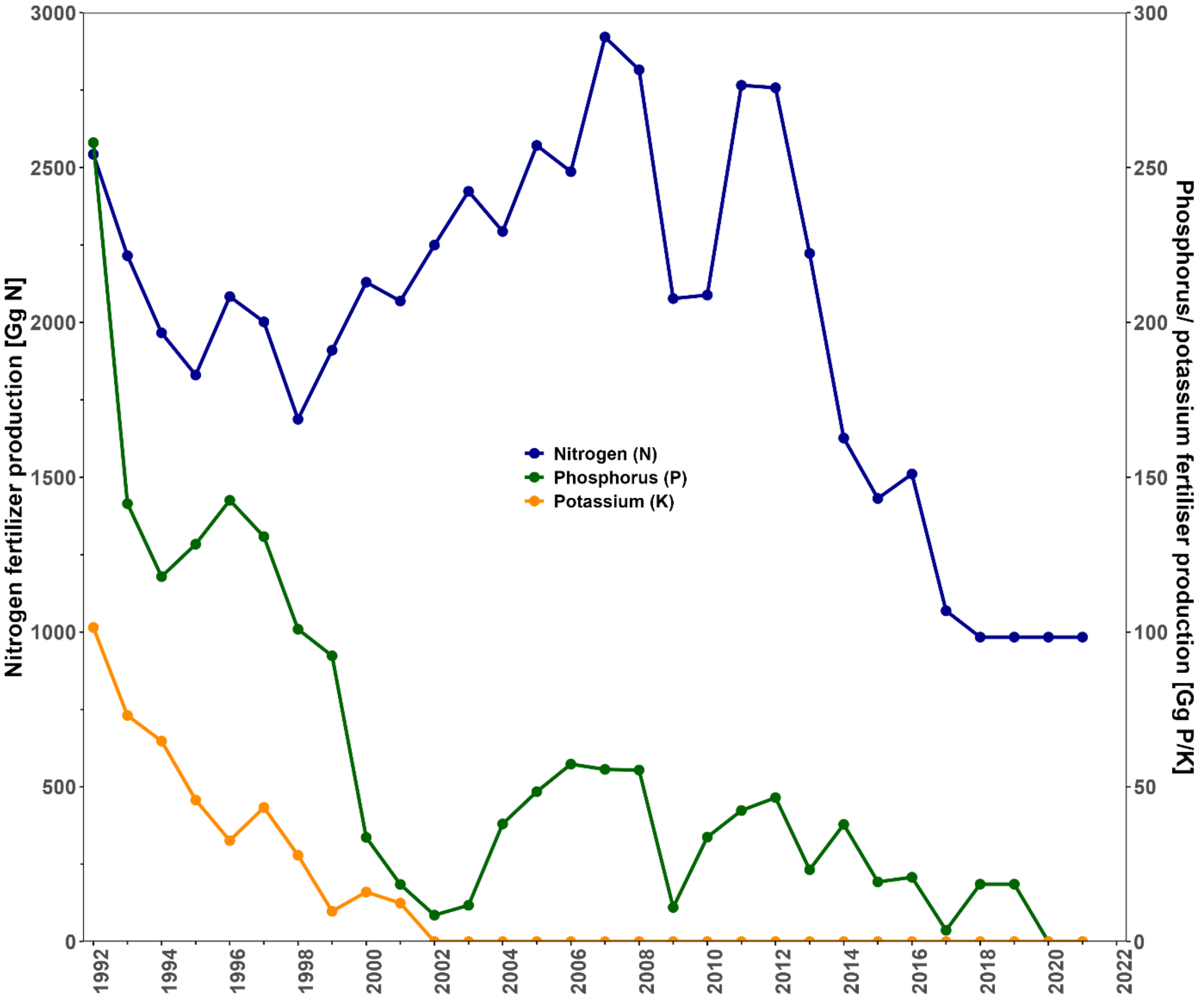

Supplementary Figure 9. Total domestic production of synthetic N, P and K fertilizers in Ukraine from 1992 to 2021 (data source: FAO, 2024). One Gg equals one thousand metric tons.

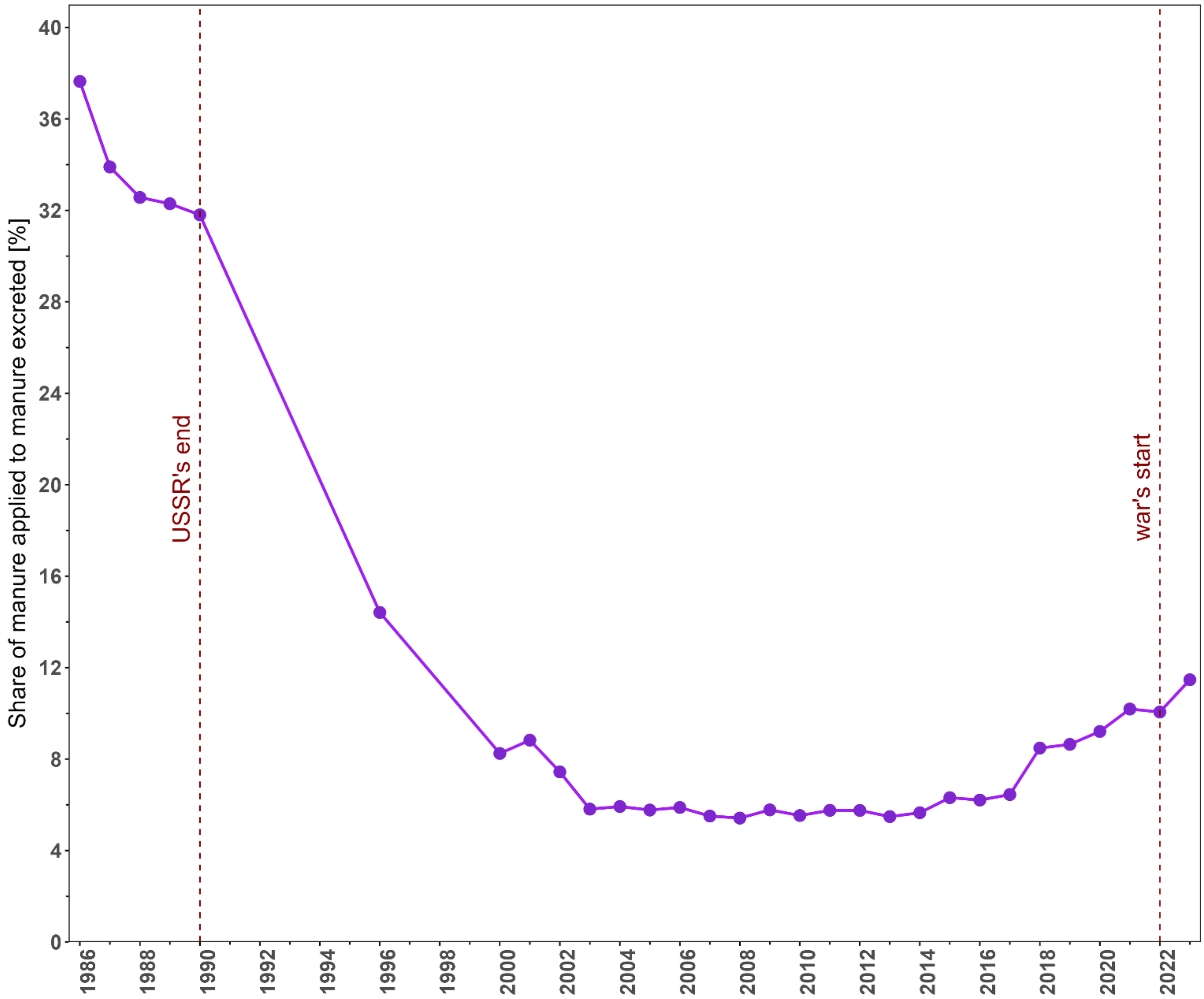

Supplementary Figure 10. The estimated mean annual manure applied to agricultural land as a share of total manure excreted from the animal husbandry sector in Ukraine from 1986 to 2023. The amount of manure applied to fields was obtained from national statistics (SSSU, 2025), while the total manure excreted from the animal husbandry sector was estimated based on animal numbers reported in national statistics (SSSU, 2025; **Supplementary Fig. 6**). Dashed lines represent the final year of USSR era and the beginning of the large-scale war in Ukraine.

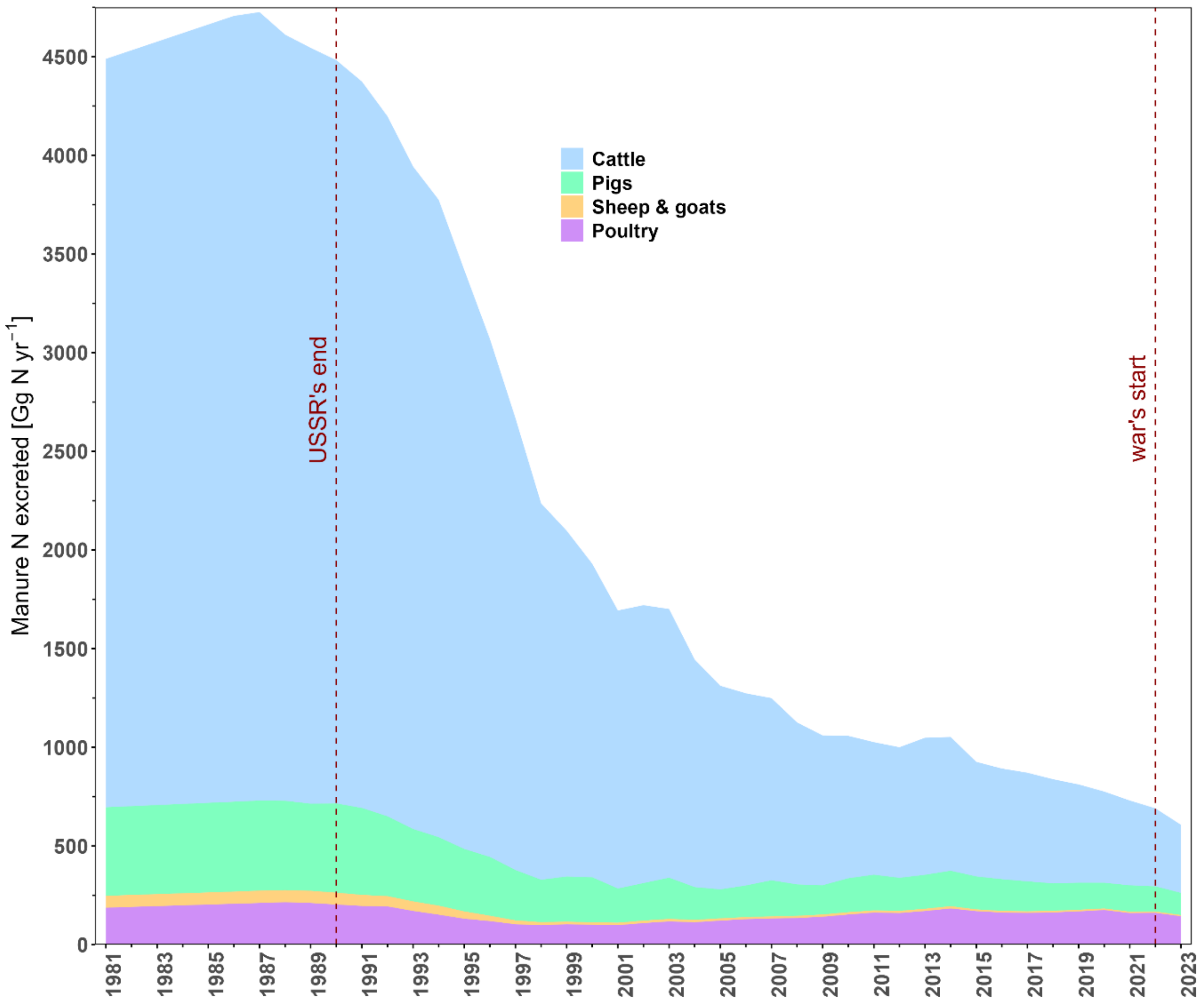

Supplementary Figure 11. Estimated production of manure (as N) from the animal husbandry sector in Ukraine from 1981 to 2023. Animal numbers were sourced from SSSU (2025) (**Supplementary Figure 6**), and average N manure excretion coefficients for each animal category, estimated for Eastern Europe, were taken from IPCC (2006). One Gg equals one thousand metric tonnes.

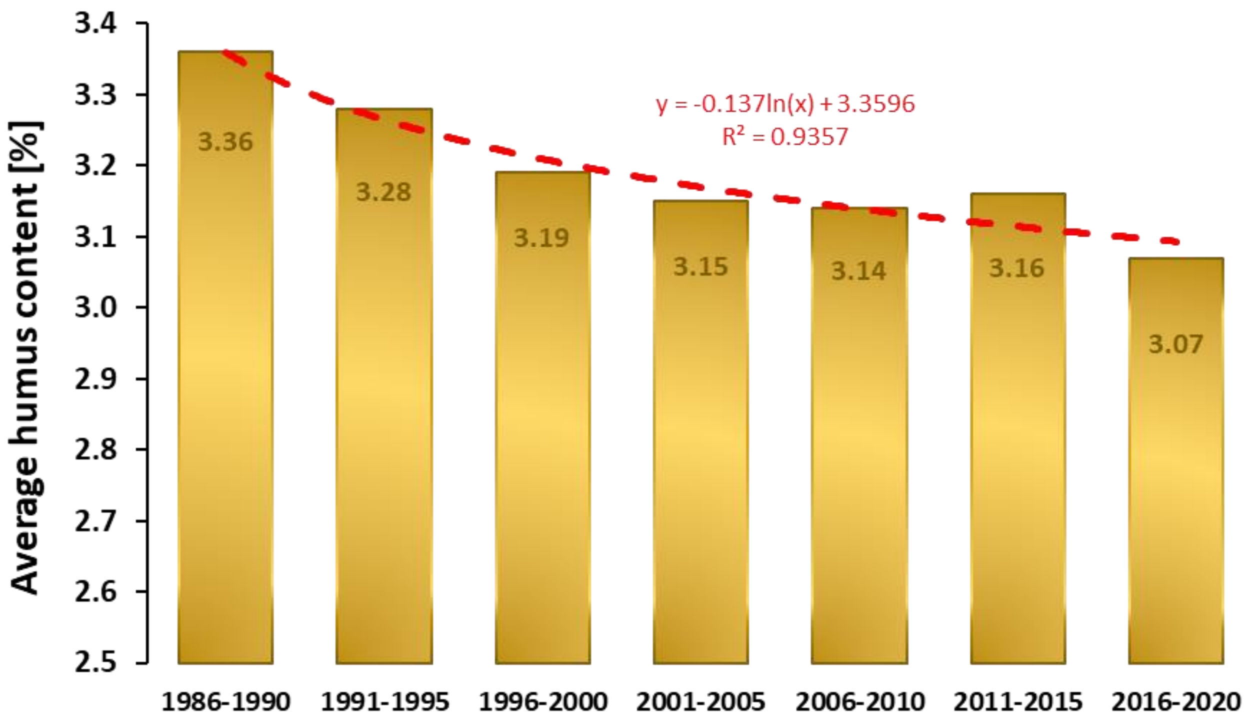

Supplementary Figure 12. Five-year average dynamics of humus content (%) in surveyed agricultural soils across Ukraine, based on seven agrochemical survey rounds conducted by the Institute of Soil Protection of Ukraine from 1986 to 2020 (adapted from Romanova, 2023). These data represent soil samples collected from the topsoil (0–20/30 cm depth, depending on A-horizon thickness). The average ratio of humus content to soil organic carbon in black soils is 1.88. For technical details, see **Supplementary Note 2.2**.

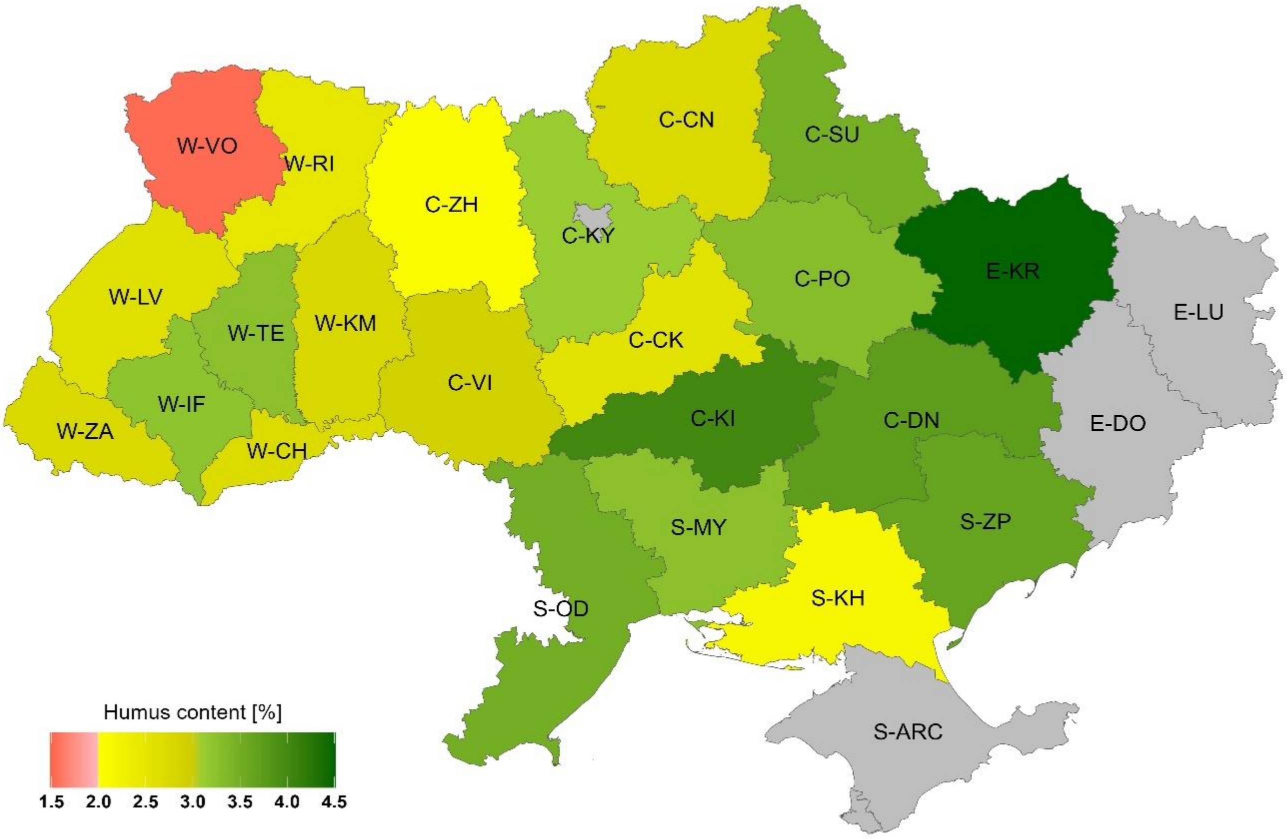

Supplementary Figure 13. Five-year average humus content (%) in surveyed agricultural soils of Ukrainian counties, based on the 2016-2020 agrochemical survey round conducted by the Institute of Soil Protection of Ukraine (adapted from Romanova, 2023). Grey-filled counties indicate no data; see **Supplementary Table 1 and Fig. 17** for county details. These data represent soil samples collected from the topsoil (0–20/30 cm depth, depending on A-horizon thickness). The average ratio of humus content to soil organic carbon in black soils is 1.88. For technical details, see **Supplementary Note 2.2**.

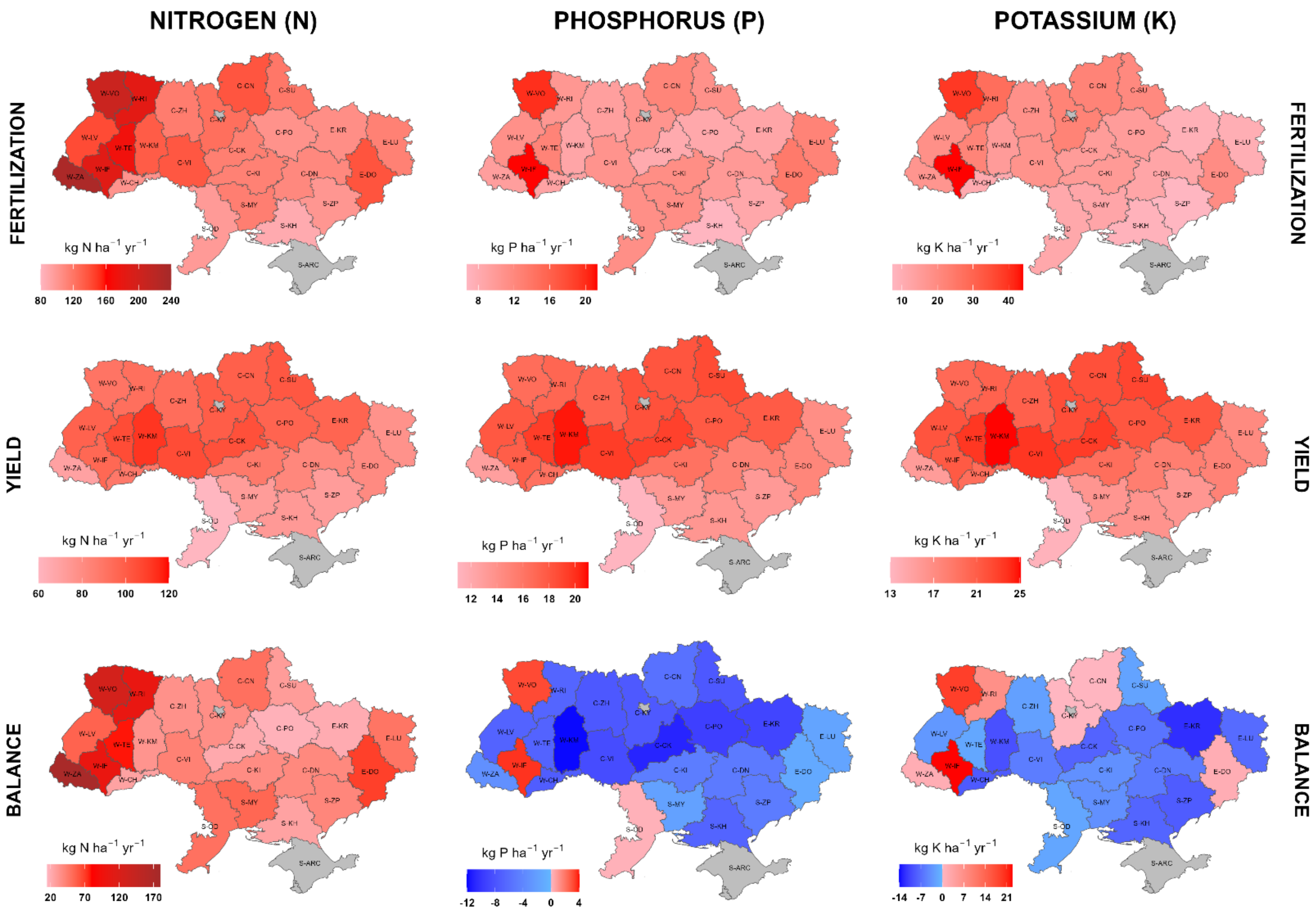

Supplementary Figure 14. Average N, P and K fertilizer inputs, yields and balances for wheat per county in Ukraine during the pre-war years 2019-2021. Annual inorganic N deposition (Supplementary Fig. 5) and annual N fixation by free-living organisms (assumed to be 5 kg N ha^{-1} yr^{-1}; Herridge *et al.*, 2008), both not shown in this figure, were used as N inputs for N balance calculations (see Methods for details). Grey-filled counties indicate no data; see **Supplementary Table 1 and Fig. 17** for county details.

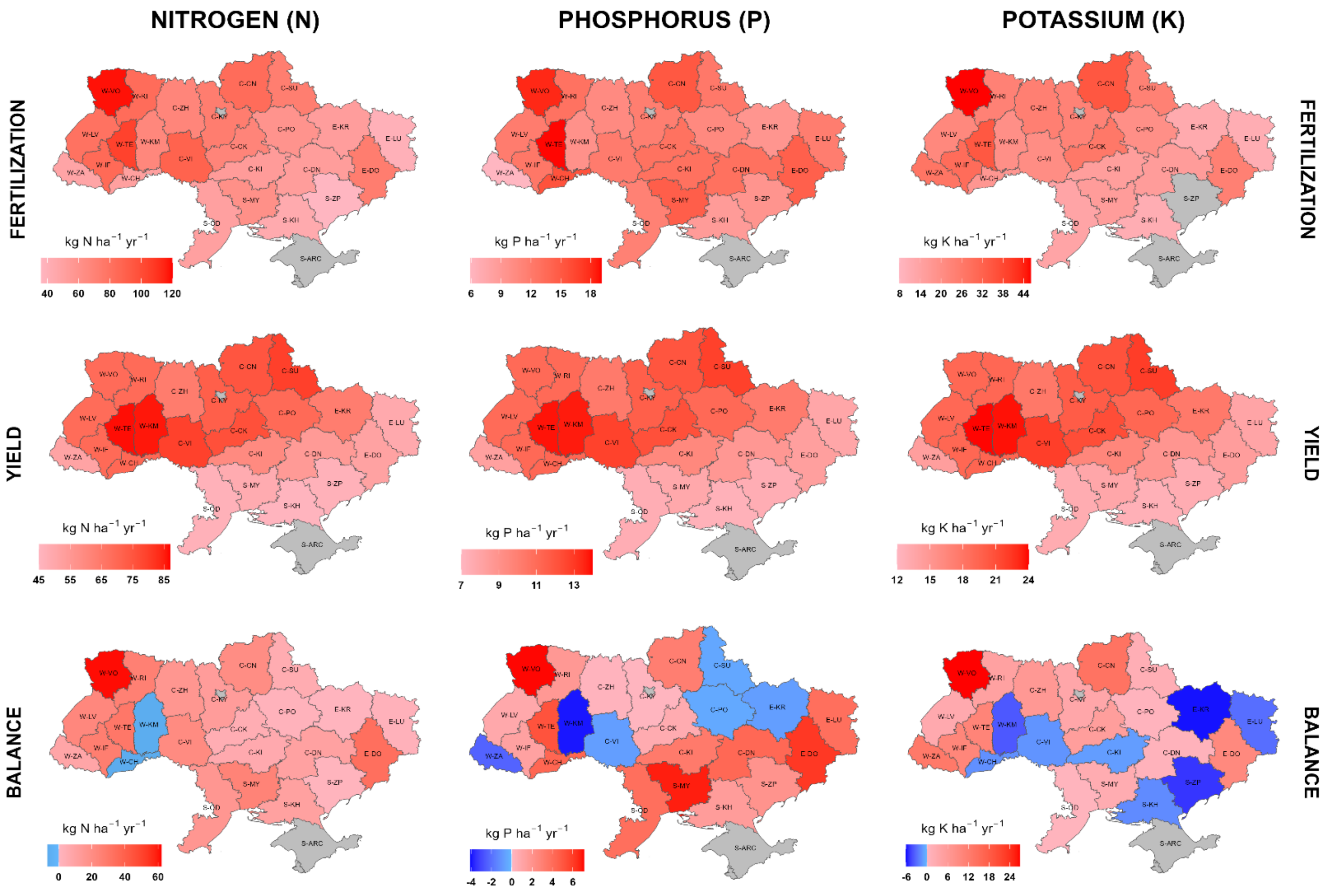

Supplementary Figure 15. Average N, P and K fertilizer inputs, yields and balances for sunflower per county in Ukraine during pre-war years 2019-2021. Annual inorganic N deposition (Supplementary Fig. 5) and annual N fixation by free-living organisms (assumed to be 5 kg N ha^{-1} yr^{-1}; Herridge *et al.*, 2008), both not shown in this figure, were used as N inputs for N balance calculations (see Methods for details). Grey-filled counties indicate no data; see **Supplementary Table 1 and Fig. 17** for county details.

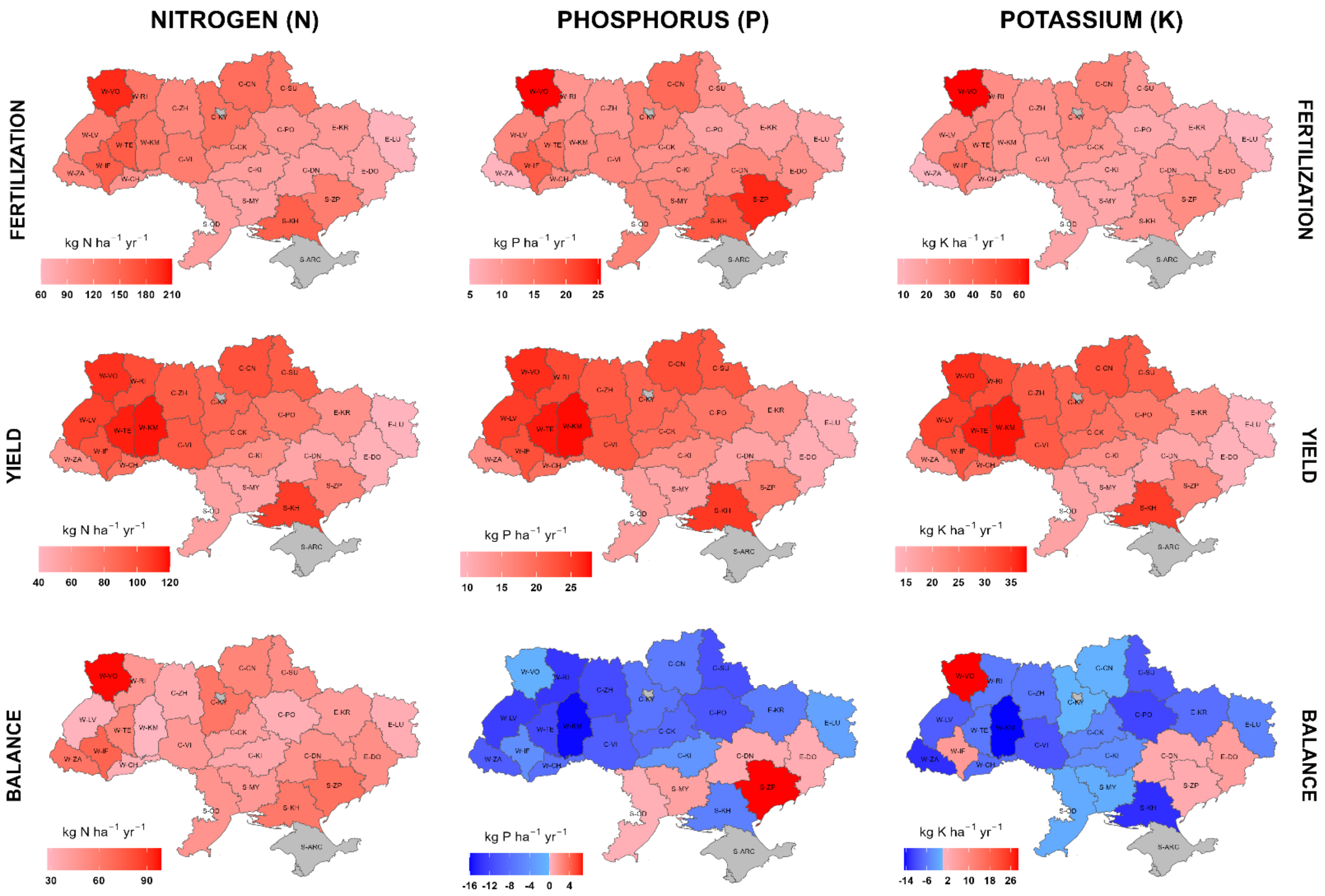

Supplementary Figure 16. Average N, P and K fertilizer inputs, yields and balances for maize per county in Ukraine during the pre-war years 2019-2021. Annual inorganic N deposition (Supplementary Fig. 5) and annual N fixation by free-living organisms (assumed to be 5 kg N ha^{-1} yr^{-1}; Herridge *et al.*, 2008), both not shown in this figure, were used as N inputs for N balance calculations (see Methods for details). Grey-filled counties indicate no data; see **Supplementary Table 1 and Fig. 17** for county details.

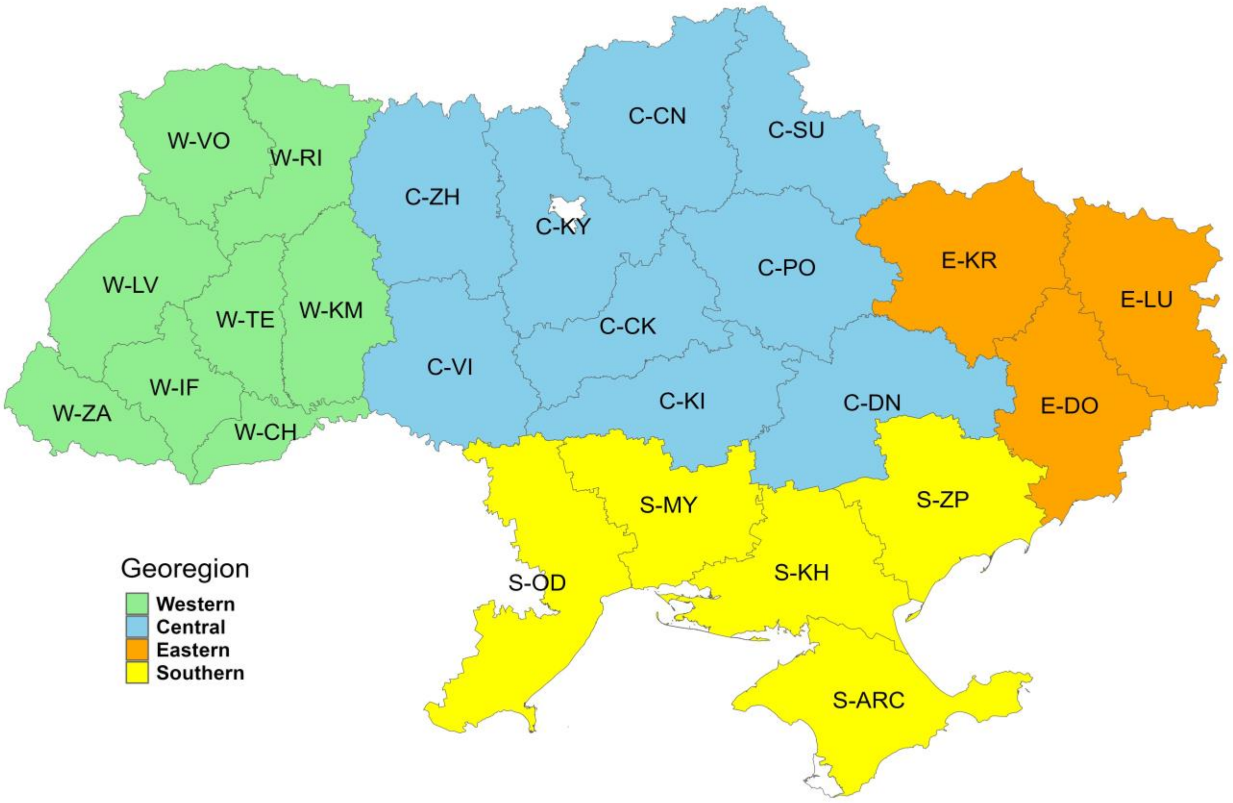

Supplementary Figure 17. Map of Ukraine showing the four main georgaphical regions and the 25 counties. The county codes shown are explained in **Supplementary Table 1**.

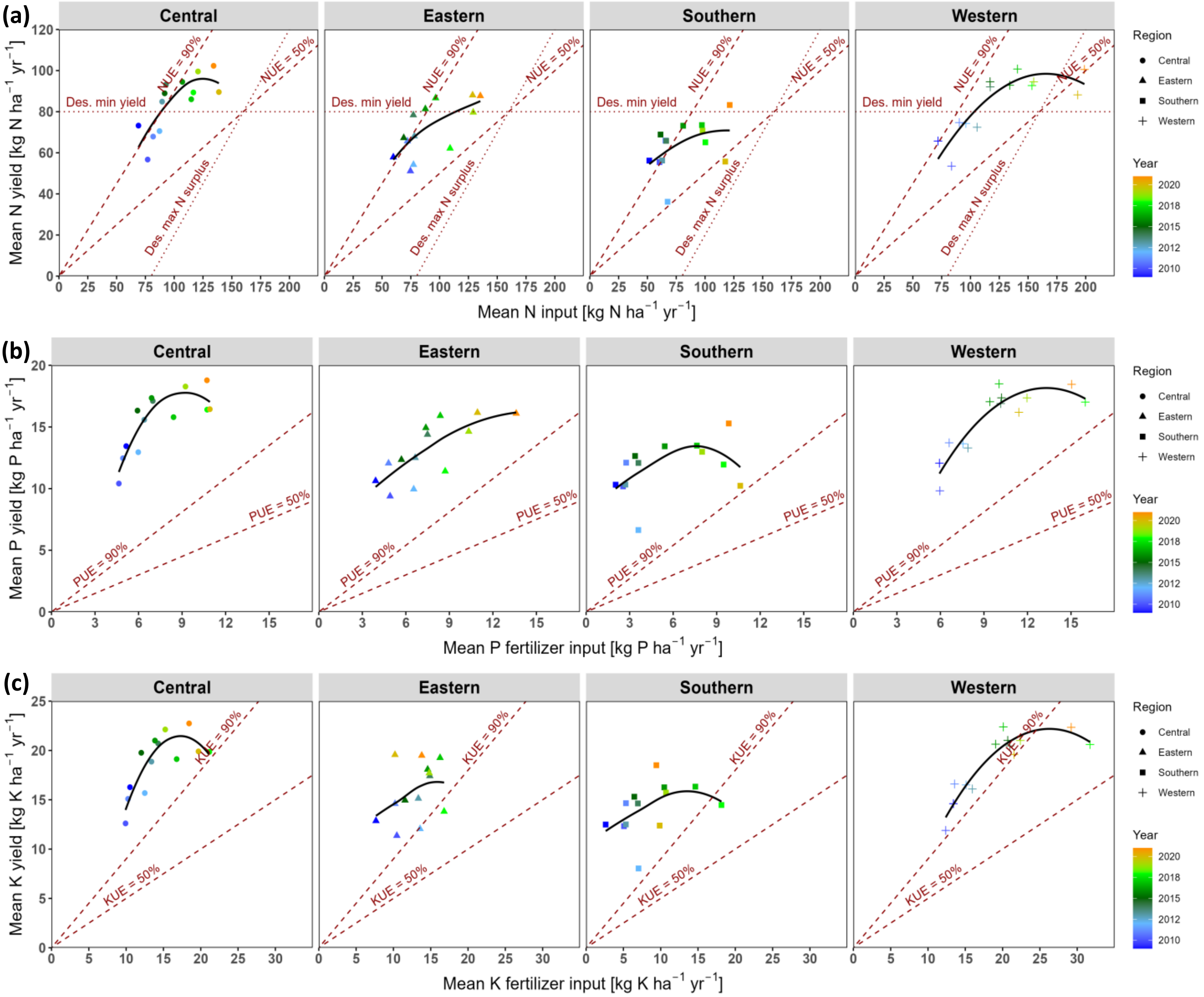

Supplementary Figure 18. Annual mean use efficiencies for N (a), P (b) and K (c) inputs in wheat cropping systems per geographical region of Ukraine from 2009 to 2021. See **Supplementary Table 1 and Figure 17** for the list and locations of counties included in each region. N input includes N fertilizer application, atmospheric inorganic N deposition and N fixation by free-living organisms; P and K inputs are equivalent to their respective fertilizer applications. The black lines were produced using local regression models (LOESS), a non-parametric approach that fits multiple regressions in local neighborhoods. The dark red lines represent nominal lower and upper target values for N, P and K use efficiency (NUE, PUE, KUE respectively) according to EUNEP (2016). Values less than 50% indicate low fertilizer use efficiency with low yields and high risk of pollution. Values larger than 90% indicate a risk of unsustainable depletion of soil nutrient stocks (‘soil mining’ of nutrients). Values of desired minimum N yield and desired maximum N surplus are also shown, as specified by EUNEP (2016).

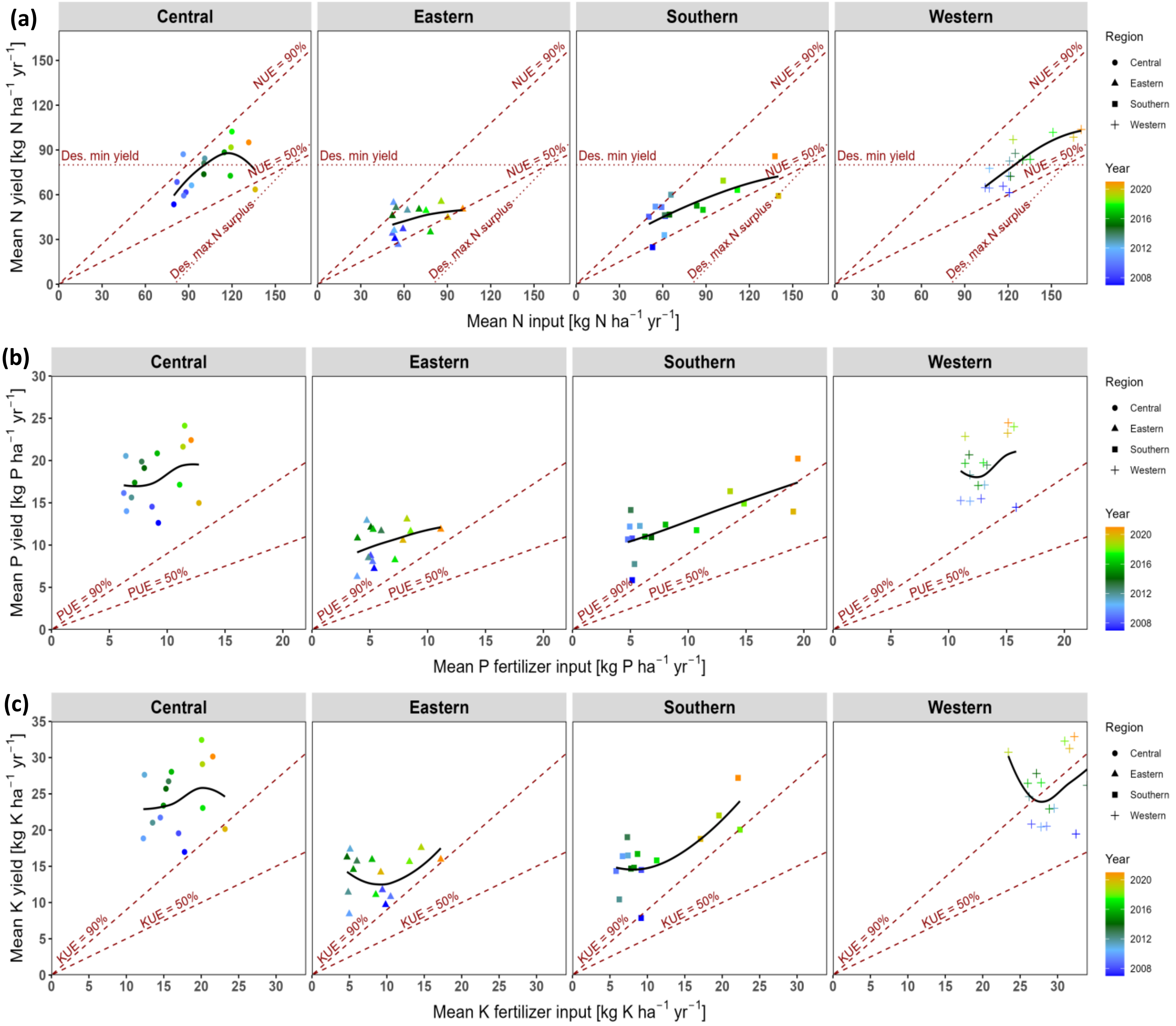

Supplementary Figure 19. Annual mean use efficiencies for N (a), P (b) and K (c) fertilizers in maize cropping systems per geographical region of Ukraine from 2007 to 2021. See **Supplementary Table 1 and Figure 17** for the list and locations of counties included in each region. N input includes N fertilizer application, atmospheric inorganic N deposition and N fixation by free-living organisms; P and K inputs are equivalent to their respective fertilizer applications. The black lines were produced using local regression models (LOESS), a non-parametric approach that fits multiple regressions in local neighborhoods. The dark red lines represent nominal lower and upper target values for N, P and K use efficiency (NUE, PUE, KUE respectively) according to EUNEP (2016). Values less than 50% indicate low fertilizer use efficiency with low yields and high risk of pollution. Values larger than 90% indicate a risk of unsustainable depletion of soil nutrient stocks (‘soil mining’ of nutrients). Values of desired minimum N yield and desired maximum N surplus are also shown, as specified by EUNEP (2016).

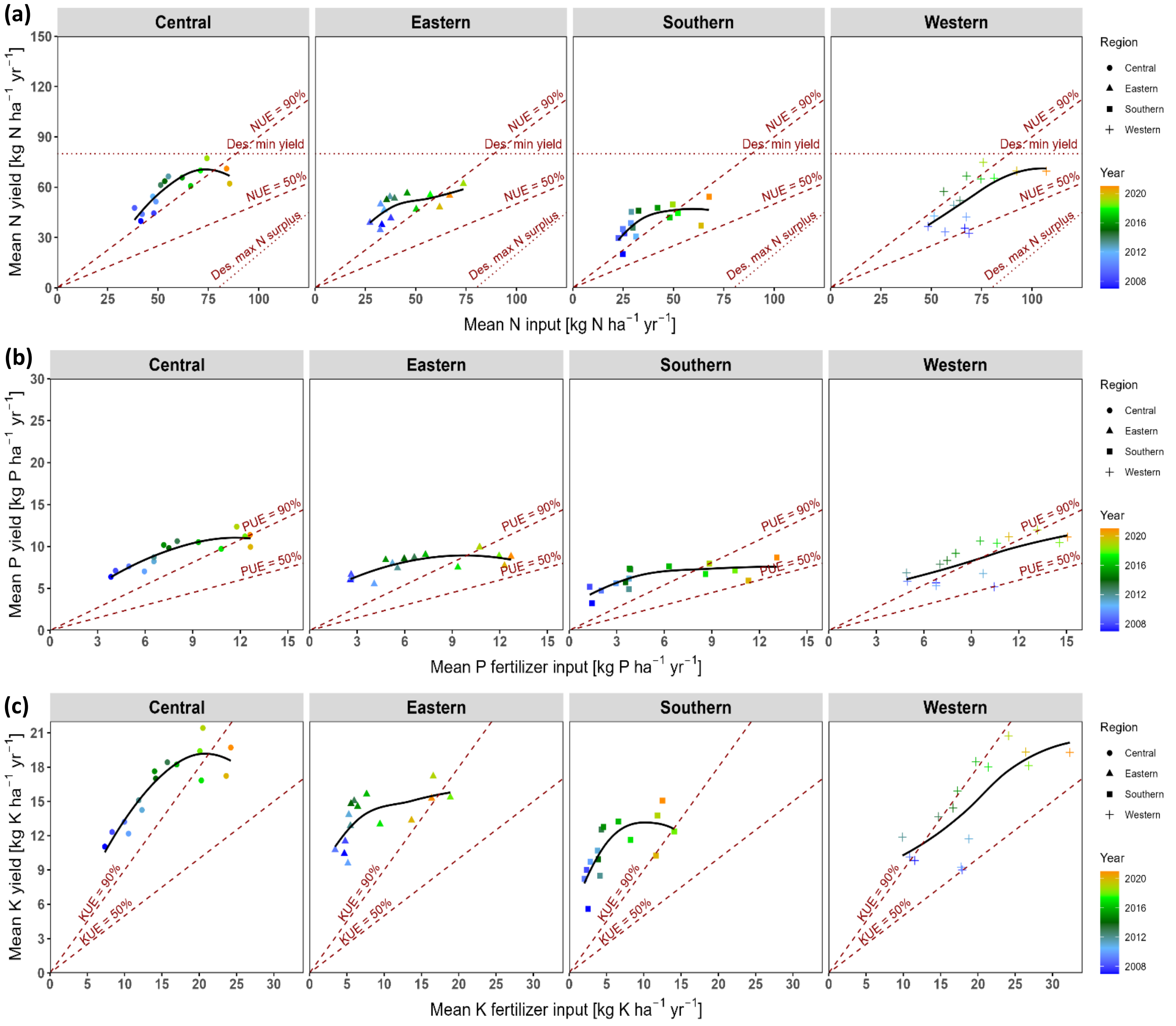

Supplementary Figure 20. Annual mean use efficiencies for N (a), P (b) and K (c) fertilizers in sunflower cropping systems per geographical region of Ukraine from 2007 to 2021. See **Supplementary Table 1 and Figure 17** for the list and locations of counties included in each region. N input includes N fertilizer application, atmospheric inorganic N deposition and N fixation by free-living organisms; P and K inputs are equivalent to their respective fertilizer applications. The black lines were produced using local regression models (LOESS), a non-parametric approach that fits multiple regressions in local neighborhoods. The dark red lines represent nominal lower and upper target values for N, P and K use efficiency (NUE, PUE, KUE respectively) according to EUNEP (2016). Values less than 50% indicate low fertilizer use efficiency with low yields and high risk of pollution. Values larger than 90% indicate a risk of unsustainable depletion of soil nutrient stocks (‘soil mining’ of nutrients). Values of desired minimum N yield and desired maximum N surplus are also shown, as specified by EUNEP (2016).

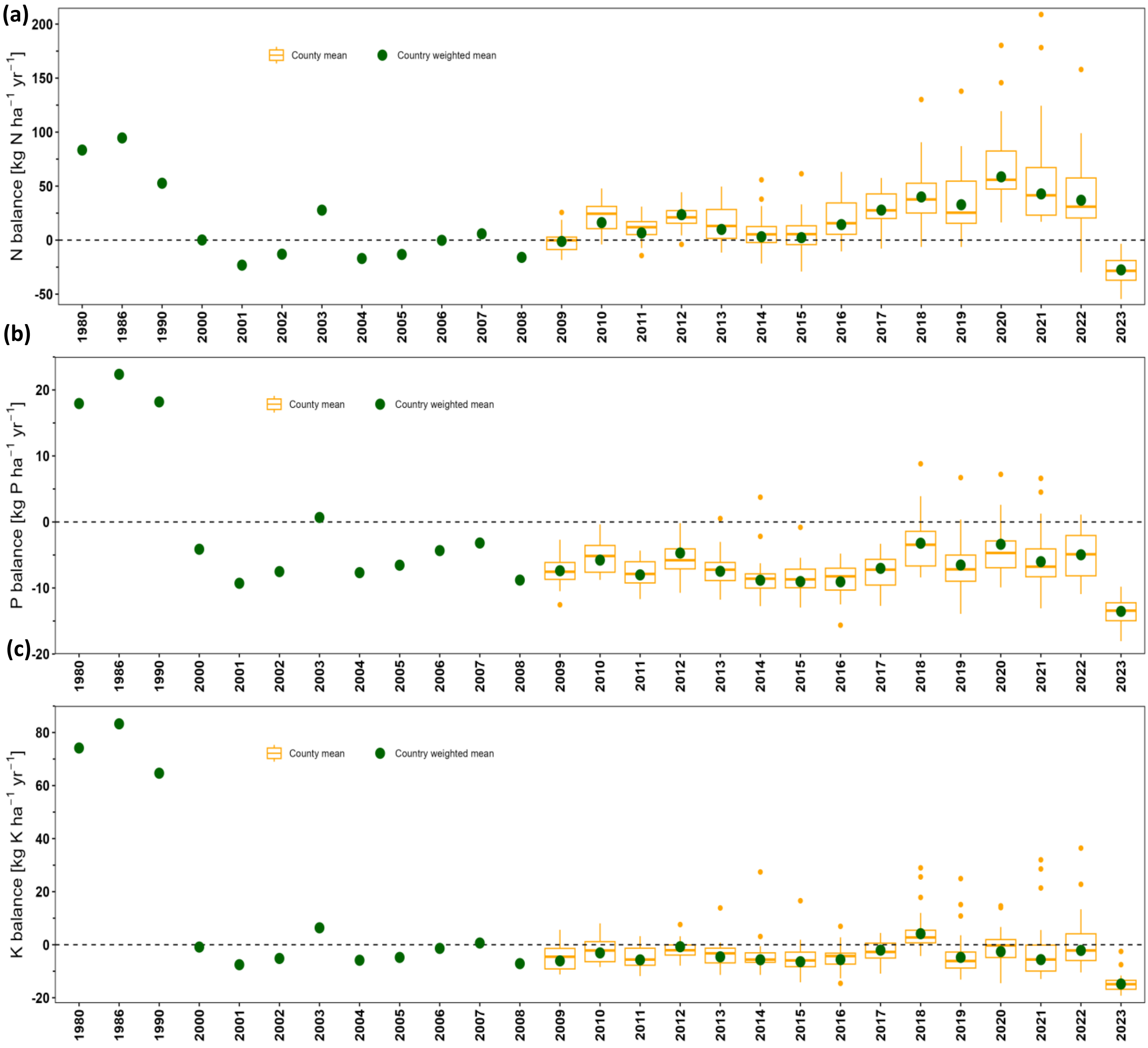

Supplementary Figure 21. Temporal and spatial variation in annual N (a), P (b) and K (c) balances for wheat at county and country scales in Ukraine over 1990 and 2000-2023. County data are available from 2009, for which the boxes show the medians (central horizontal lines) and interquartile range (upper and lower horizontal lines), the vertical yellow lines show minimum and maximum values within the 95% confidence interval and the yellow points represent outliers beyond 95%.

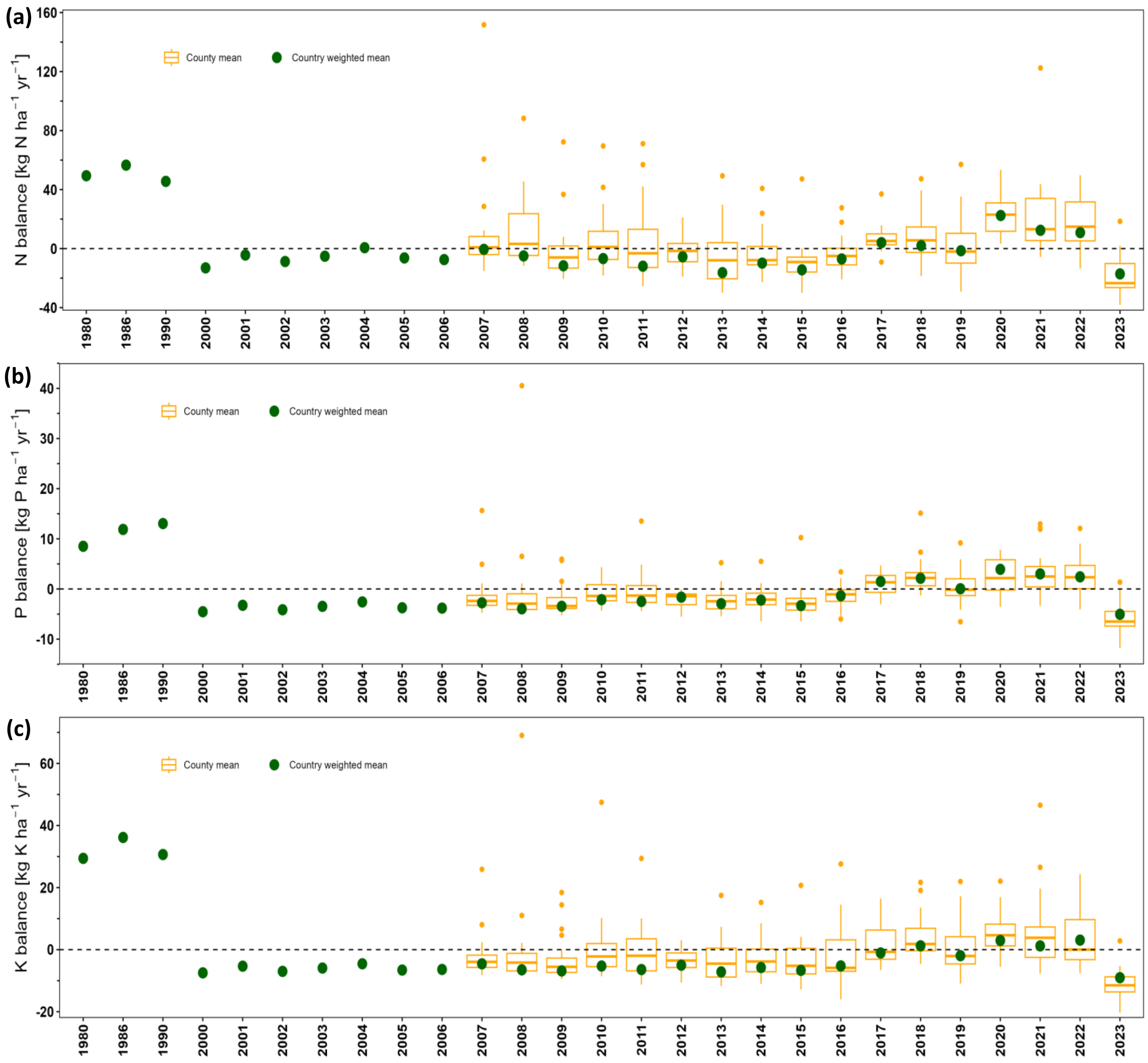

Supplementary Figure 22. Temporal and spatial variation in annual N (a), P (b) and K (c) balances for sunflower at county and country scales in Ukraine over 1990 and 2000-2023. County data are available from 2007, for which the boxes show the medians (central horizontal lines) and interquartile range (upper and lower horizontal lines), the vertical yellow lines show minimum and maximum values within the 95% confidence interval and the yellow points represent outliers beyond 95%.

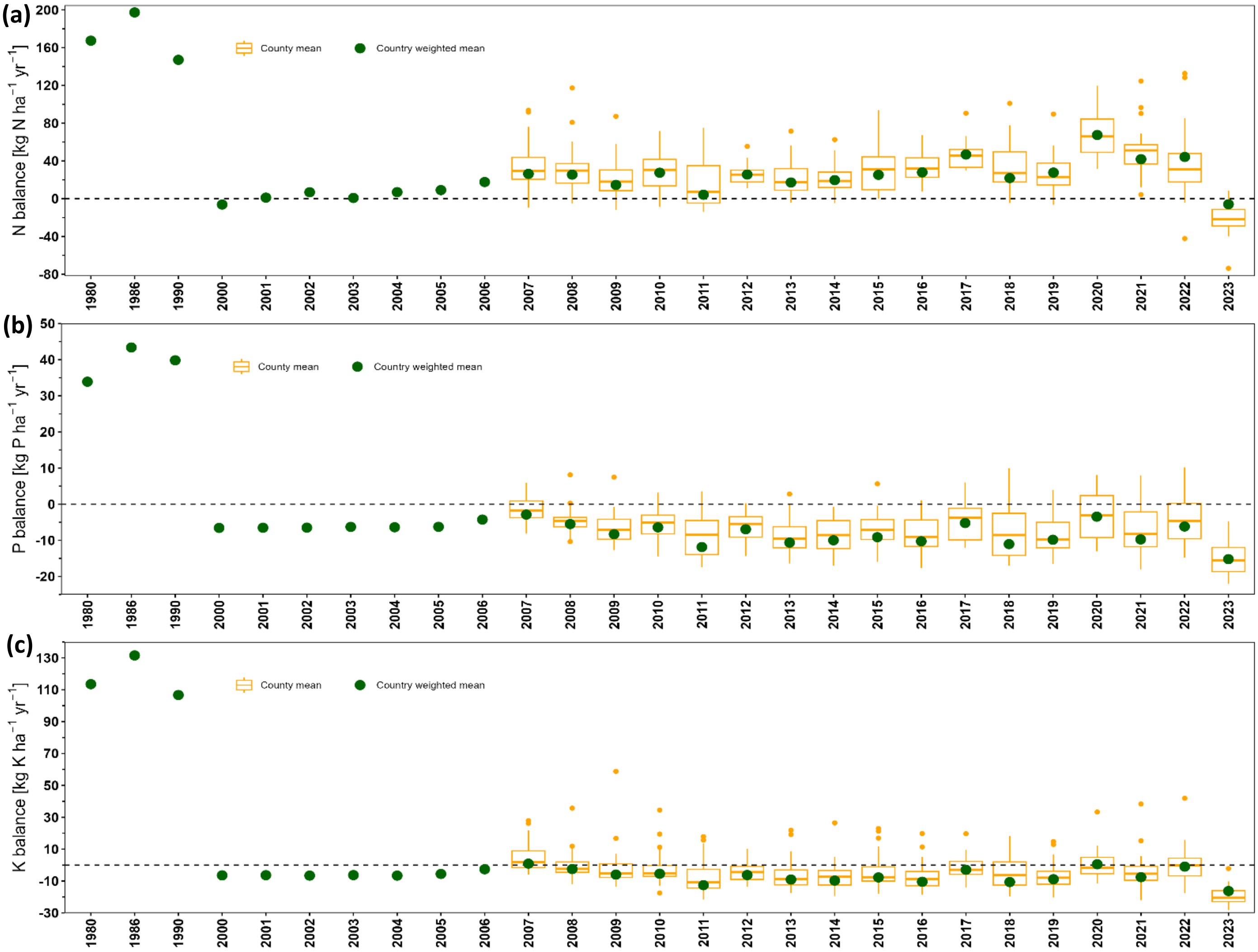

Supplementary Figure 23. Temporal and spatial variation in annual N (a), P (b) and K (c) balances for maize at county and country scales in Ukraine over 1990 and 2000-2023. County data are available from 2007, for which the boxes show the medians (central horizontal lines) and interquartile range (upper and lower horizontal lines), the vertical yellow lines show minimum and maximum values within the 95% confidence interval and the yellow points represent outliers beyond 95%.

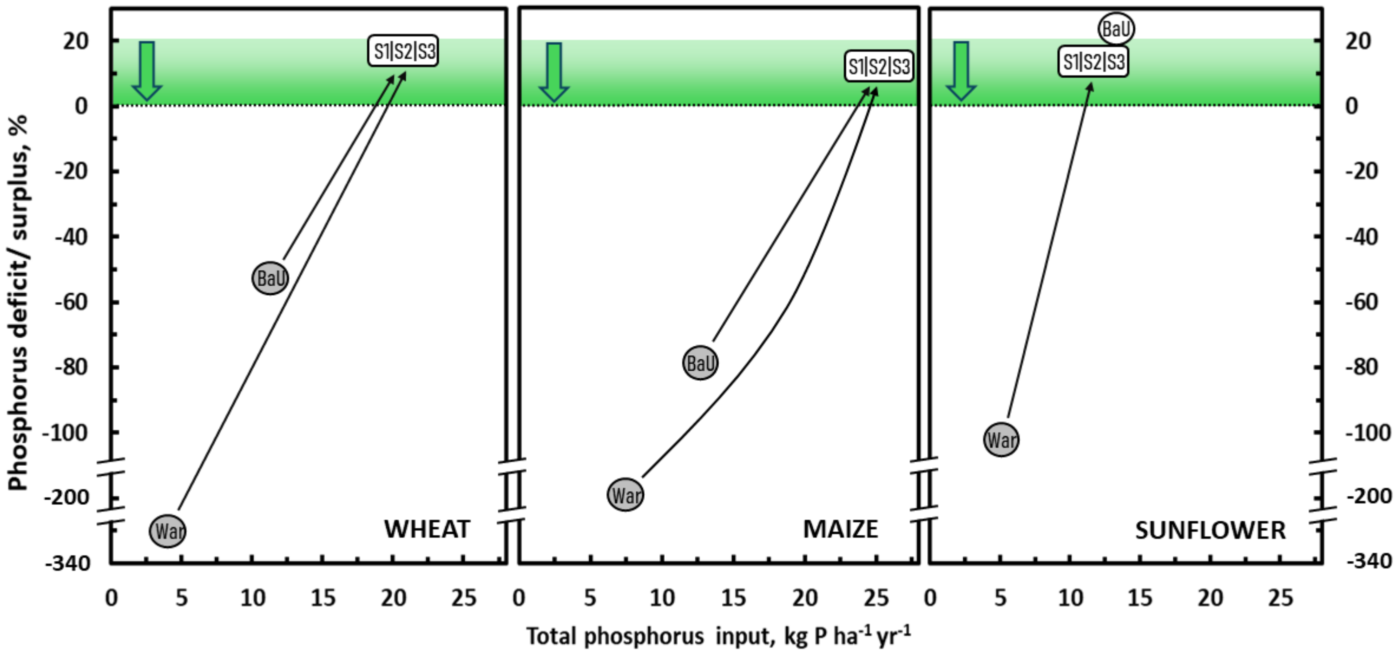

Supplementary Figure 24. Average phosphorus (P) surplus or deficit as a percentage of total P input for wheat, maize and sunflower in Ukraine under five contrasting scenarios for 2030. The business-as-usual scenario (BaU; S-0) reflects the continuation of 2021 agricultural practices prior to the Russian invasion. The extended war disruption scenario (War; S-w) assumes prolonged fertilizer shortages at 2023 levels. The manure-enriched precision fertilizer scenario (S1; S-1) involves the substitution of synthetic N with manure-N, increasing its share by 30%, combined with precision fertilizer application. The enhanced efficiency fertilizer scenario (S2; S-2) builds upon S1 by incorporating nitrification inhibitors and slow-release fertilizers. The legume-based diversification scenario (S3; S-3) introduces optimized crop rotations with legumes, in combination with S2 or S1 (see Nutrient Management Scenarios). Scenario colours indicate a surplus (white) or a deficit (grey). The green-shaded belt represents the acceptable P surplus range, between 0% and 20% of total P input (as a portion of manure-P remains crop-unavailable during the first year of application; see **Supplementary Table 2, Note 2.8**). Arrows indicate the direction of surplus reduction. The dotted horizontal line marks zero P balance (equilibrium) with surpluses above and deficits below this threshold.

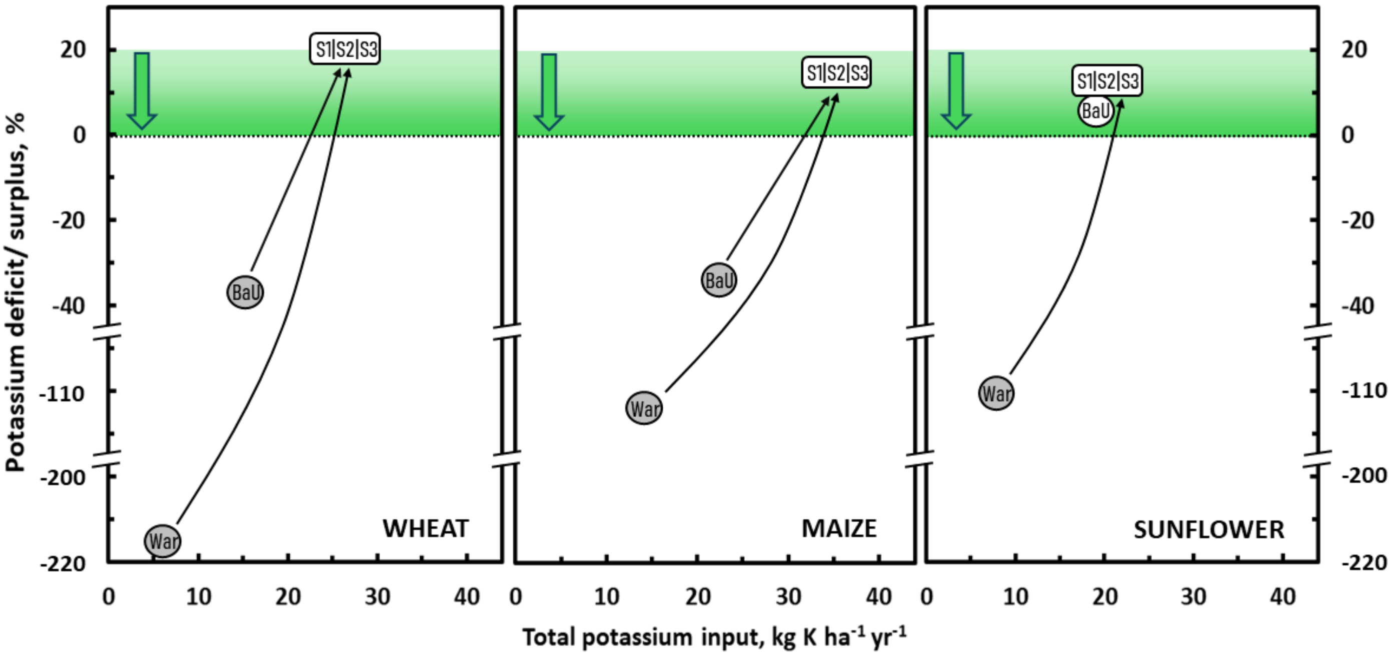

Supplementary Figure 25. Average potassium (K) surplus or deficit as a percentage of total K input for wheat, maize and sunflower in Ukraine under five contrasting scenarios for 2030. The business-as-usual scenario (BaU; S-0) reflects the continuation of 2021 agricultural practices prior to the Russian invasion. The extended war disruption scenario (War; S-w) assumes prolonged fertilizer shortages at 2023 levels. The manure-enriched precision fertilizer scenario (S1; S-1) involves the substitution of synthetic N with manure-N, increasing its share by 30%, combined with precision fertilizer application. The enhanced efficiency fertilizer scenario (S2; S-2) builds upon S1 by incorporating nitrification inhibitors and slow-release fertilizers. The legume-based diversification scenario (S3; S-3) introduces optimized crop rotations with legumes, in combination with S2 or S1 (see Nutrient Management Scenarios). Scenario colours indicate a surplus (white) or a deficit (grey). The green-shaded belt represents the acceptable K surplus range, between 0% and 20% of total K input (as a portion of manure-K remains crop-unavailable during the first year of application; see **Supplementary Table 3, Note 2.8**). Arrows indicate the direction of surplus reduction. The dotted horizontal line marks zero P balance (equilibrium) with surpluses above and deficits below this threshold.

Supplementary References

Agro-Pritok (2024). Fertilizer sales. Agro-Pritok. https://agropritok.com.ua/

Altieri, K.E., Fawcett, S.E., Peters, A.J., Sigman, D.M., Hastings, M.G. (2016). Marine biogenic source of atmospheric organic nitrogen in the subtropical North Atlantic. *Proceedings of the National Academy of Sciences*, **113**, 925-930.

Baliuk, S.A. and Medvedev V.V. (2012) Strategy for Balanced Use, Reproduction, and Management of Soil Resources of Ukraine, *Agrarian Science: Kyiv*, 2012. 240 pp.

Baliuk, S.A., Kuchir, A.V., Maksymenko, N.V. (2021) Soil Resources of Ukraine: Current State, Problems, and Sustainable Management Strategy. *Ukrainian Geographical Journal*, **2**, 3-11 (In Ukrainian). https://doi.org/10.15407/ugz2021.02.003

Baliuk, S. A. *et al.* (2012). Ecological condition of the soils of Ukraine. *Ukrainian Geographical Journal,* **2**, 38-42 (in Ukrainian). https://ukrgeojournal.org.ua/uk/node/331

Baliuk, S.A., Medvedev, V.V. et al (2010) National Report on Soil Fertility Status in Ukraine. Ministry of Agrarian Policy, Center for State Soil Fertility, NAAS of Ukraine, National Scientific Center of the Institute of Agrochemistry named after O.N. Sokolovsky, NUBiP, Kyiv, 2010. 113 pp.

Baliuk, S.A., Romashchenko, M.I., Stashuk, V.A. (2009) Scientific Foundations of Irrigated Land Protection and Rational Use in Ukraine. *Agrarian Science: Kyiv*, 2009. 624 pp.

Bilanchyn Y., Tsurkan O., Tortyk M., Medinets V., Buyanovskiy A., Soltys I. and Medinets S. (2021) Post-irrigation state of Black Soils in South-Western Ukraine. In: Dent D., Boincean B. (eds). *Regenerative Agriculture.* Springer, Cham, pp. 303-309. https://doi.org/10.1007/978-3-030-72224-1_27

Boincean, B. and Dent, D. (2020). Soil fertility – the only possible foundation for more sustainable agriculture. *BIO Web of Conference,* **17**, 00119.

Boincean, B.P. and Dent, D.L. (2019). *Farming the Black Earth. Sustainable and Climate-Smart Management of Chernozem Soils.* Cham: Springer Nature.

Brown C. (2013). Available Nutrients and Value for Manure From Varous Livestock Types. Factsheet (August 2013). Ontario Ministry of Agriculture and Food and the Ministry of Rural Affairs. https://fieldcropnews.com/wp-content/uploads/2015/03/Nutrient-Value-of-Manure.pdf

Brownlie, W. J., Sutton, M. A., Reay, D. S., Heal, K. V., Hermann, L., Kabbe, C. & Spears, B. M. (2021). Global actions for a sustainable phosphorus future. *Nature Food*, **2**, 71-74.

Brownlie, W.J. *et al. Nitrogen Mitigation. INMS Guidance Document on Measures for Sustainable Nitrogen Management.* INMS Guidance Document Series. International Nitrogen Management System (UK Centre for Ecology & Hydrology, Edinburgh, 2024) https://doi.org/10.5281/zenodo.13831114

Buyanovskiy A.O., Ozhovan O.O., Tortyk M.Y. (2022) The humus state of ordinary chernozems of the Tarutyn Steppe under different conditions of their use. *Taurian Scientific Bulletin. Series: Agricultural sciences*, **128**, 328-333 (in Ukrainian) https://doi.org/10.32851/2226-0099.2022.128.45

Buyanovskiy, A. O., Tortyk M.Y. (2021) Structural and aggregate composition of ordinary chernozems of the Tarutyn Steppe under different conditions of their use. *Taurian Scientific Bulletin. Series: Agricultural sciences*, **122**, 3-10 (in Ukrainian). https://doi.org/10.32851/2226-0099.2021.122.1

Cantarella, H., Otto, R., Soares, J. R., & de Brito Silva, A. G. (2018). Agronomic efficiency of NBPT as a urease inhibitor: A review. *Journal of advanced research*, **13,** 19-27.

Cape, J.N., Cornell, S.E., Jickells, T.D., Nemitz, E. (2011). Organic nitrogen in the atmosphere - Where does it come from? A review of sources and methods. *Atmospheric Research*, **102**, 30-48.

Chesnyak, G. Ya., Gavrilyuk F.Ya, Krupenikov I.A. *et al.* (1983). Humus state of black earth. In: Russian black earth - 100 years after Dokuchaeva. *Nauka, Moskva*, pp. 186–198.

Claessens, J. *et al.* (2024). Agricultural practices and water quality in the Netherlands: status (2020–2023) and trends (1992–2023): the 2024 Nitrate report with the results of the monitoring of the effects of the EU Nitrates Directive Action Programmes. RIVM-report 2024-0209 https://doi.org/10.21945/RIVM-2024-0209

Congreves, K. A., Otchere, O., Ferland, D., Farzadfar, S., Williams, S., & Arcand, M. M. (2021). Nitrogen use efficiency definitions of today and tomorrow. *Frontiers in Plant Science*, **12,** 637108.

Cornell, S.E. (2011). Atmospheric nitrogen deposition: Revisiting the question of the importance of the organic component. *Environmental Pollution*, **159**, 2214-2222.

Cui, Z. *et al.* (2018). Pursuing sustainable productivity with millions of smallholder farmers. *Nature*, **555**, 363-366. https://doi.org/10.1038/nature25785

Demetra-Agro (2024). Fertilizer sales. Trade house ‘Demetra-Agro’ https://demetra-agro.com/katalog-tovariv/

Dobrivo.com (2024). Fertilizer sales. Dobrivo.com https://dobrivo.com/

DSTU 4114-2002 (2002). Soils. Determination of dynamic compounds of phosphorus and potassium by the modified method of Machihyn (in Ukrainian).

DSTU 4289:2004 (2004). Soil quality. Methods for determination of organic substance (in Ukrainian).
DSTU 4727:2007 (2007). Soil quality. Determination of labile phosphorus compounds by Karpinsky-Zamyatna method in the version of NNTs IGA after A.N. Sokolovsky (in Ukrainian).
DSTU 7863:2015 (2015). Soil quality. Determination of easily hydrolyzable nitrogen by the Kornfield method (in Ukrainian).
ECE/EB.AIR/119 (2013) UNECE Guidance document on national nitrogen budgets. https://unece.org/DAM/env/documents/2013/air/eb/ECE_EB.AIR_119_ENG.pdf
Ecoaction.org.ua (2023). Land pollution as a result of Russia's aggression against Ukraine / A. Splodytel, O. Holubtsov, S. Chumachenko, L. Sorokina – Kyiv: NGO "Ecoaction", 154 pp. https://ecoaction.org.ua/wp-content/uploads/2023/03/zabrudnennia-zemel-vid-rosii-full3.pdf
EUNEP (2016). Nitrogen Use Efficiency (NUE) - Guidance Document for Assessing NUE at Farm Level. EU Nitrogen Expert Panel. Wageningen University, Alterra, Netherlands. https://www.eunep.com/wp-content/uploads/2023/12/NUE-Guidance-Document.pdf
FAO (2005). Fertilizer use by crop in Ukraine. UN FAO, Rome, 55 p. https://www.fao.org/4/a0225e/a0225e00.pdf
FAO (2018). FAO kicks off project aimed at tackling land degradation in Ukraine. Press Release (May 10, 2018). FAO Regional Office for Europe and Central Asia. https://www.fao.org/europe/news/detail/-FAO-kicks-off-project-aimed-at-tackling-land-degradation-in-Ukraine/
FAO (2019). Ukraine, FAO unite to save healthy soil. Press Release (May 24, 2019). FAO Regional Office for Europe and Central Asia. https://www.fao.org/europe/news/detail/Ukraine-FAO-unite-to-save-healthy-soil/
FAO (2023a). Ukraine: Humanitarian response update, 5 December 2023. Kyiv. https://openknowledge.fao.org/server/api/core/bitstreams/65f80020-6140-4a4e-9a89-0c75305c72bf/content
FAO (2023b). *Ukraine: Impact of the war on agricultural enterprises – Findings of a nationwide survey of agricultural enterprises with land up to 250 hectares, January–February 2023.* Rome. https://doi.org/10.4060/cc5755en
FAO (2024a). Emergency grant aid for the global food security. Ukraine. Project Highlights. https://openknowledge.fao.org/server/api/core/bitstreams/f06074ad-caaf-4783-ac71-ea776430d2eb/content
FAO (2024b). Fertilizer by Nutrient. FAOSTAT database. Food and Agriculture Organization of the United Nations. https://www.fao.org/faostat/en/#data/RFN
Farming during the war (2023). *Documentary series* (November 30, 2023). https://youtu.be/4TRiXqi_UOE?si=4SLtMUp1gguu1PW9
Geng, S., Tan, J., Li, L., Miao, Y., & Wang, Y. (2023). Legumes can increase the yield of subsequent wheat with or without grain harvesting compared to Gramineae crops: A meta-analysis. *European Journal of Agronomy*, *142*, 126643. https://doi.org/10.1016/j.eja.2022.126643
GRCDC (2019). Phosphorus and phosphorus stratification. Grain Research & Development Corporation of Australian Government https://grdc.com.au/resources-and-publications/grdc-update-papers/tab-content/grdc-update-papers/2019/02/phosphorus-and-phosphorus-stratification
Guardia, G., Cangani, M. T., Andreu, G., Sanz-Cobena, A., García-Marco, S., Álvarez, J. M., ... & Vallejo, A. (2017). Effect of inhibitors and fertigation strategies on GHG emissions, NO fluxes and yield in irrigated maize. *Field Crops Research*, **204,** 135-145.
Guardia, G., Cangani, M. T., Sanz-Cobena, A., Junior, J. L., & Vallejo, A. (2017). Management of pig manure to mitigate NO and yield-scaled N2O emissions in an irrigated Mediterranean crop. *Agriculture, Ecosystems & Environment*, ***238,*** 55-66.
Herridge, D.F. *et al.* (2008). Global inputs of biological nitrogen fixation in agricultural systems. *Plant Soil* **311**, 1-18.
Jemo, M., Abaidoo, R. C., Nolte, C., Tchienkoua, M., Sanginga, N., & Horst, W. J. (2006). Phosphorus benefits from grain-legume crops to subsequent maize grown on acid soils of southern Cameroon. *Plant and Soil*, **284,** 385-397.
Iqbal, J., Johnson, L., Schmidt, A.M., Kurtzhals, A. (2022). New UNL Manure Nitrogen Crediting Recommendations for Crop Fertility. Communication. University of Nebraska-Lincoln (UNL). https://water.unl.edu/article/animal-manure-management/new-unl-manure-nitrogen-crediting-recommendations-crop-fertility/
INPC (2011). Integrated Nutrient Pollution Control Project (Romania) under the WB-GEF Investment Fund for Nutrient Reduction in the Danube River and Black Sea. Final Report. https://iwlearn.net/resolveuid/fdef9cef8d622e900590926f0ec80bf0 http://documents1.worldbank.org/curated/en/967941468143710927/pdf/710170PJPR0P090064B00PUBLIC000SEC0.pdf
INPC (2019). Integrated Nutrient Pollution Control Project (Romania). Promoting Replication of Good Practices for Nutrient Reduction and Joint Collaboration in Central and Eastern Europe. https://iwlearn.net/resolveuid/cab7e969-df25-4327-b7f8-244bfff584be
INPC (2022). Integrated Nutrient Pollution Control. Ministry of Environment, Waters and Forests of Romania. http://www.inpcp.ro/en

IPCC (2006). *Guidelines for National Greenhouse Gas Inventories*. 2006. GHG Inventory. https://www.ipcc-nggip.iges.or.jp/public/2006gl/russian/pdf/4_Volume4/V4_10_Ch10_Livestock.pdf
IPNI (2014). IPNI Estimates of Nutrient Uptake and Removal. The International Plant Nutrition Institute. http://www.ipni.net/article/ipni-3296
ISO 11263:1994 (1994). Soil quality — Determination of phosphorus — Spectrometric determination of phosphorus soluble in sodium hydrogen carbonate solution.
Kernasyuk Yu. (2024). Global and domestic trends in the fertilizer market (September 12th, 2024). Press article on Agro-business.com.ua (in Ukrainian). https://agro-business.com.ua/agro/ekonomichnyi-hektar/item/30572-hlobalni-i-vnutrishni-trendy-na-rynku-dobryv.html
Khrystenko, A. O., Istomina, Yu. O. (2013). Soil and climatic conditions of Ukraine and the effectiveness of potash fertilizers. *Herald of Agrarian Science*, **76**, 10-13 (in Ukrainian).
Khrystenko, A. O., Lazebna, M. E. (2008). Assessment of the phosphate state of soils based on the International Standard. *Herald of Agrarian Science*, **19**, 16-19 (in Ukrainian).
Khrystenko, A. O. (2023). A calculation method for determining the level of supply of plants with macronutrients. *AgroChemistry and Soil Science*, **94**, 15-21 (in Ukrainian).
Klein, H., Gauss, M., Tsyro, S., Nyiri, A. (2024) Transboundary air pollution by sulphur, nitrogen, ozone and particulate matter in 2022. Ukraine. MSC-W Data Note 1/2024 (August 2024). https://emep.int/publ/reports/2024/Country_Reports/report_UA.pdf
Klimczyk, M., Siczek, A., & Schimmelpfennig, L. (2021). Improving the efficiency of urea-based fertilization leading to reduction in ammonia emission. *Science of the Total Environment*, *771*, 145483
KMU (2022). Small agricultural producers can receive assistance through the State Agrarian Register (September 5, 2022) (in Ukrainian). https://www.kmu.gov.ua/news/mali-ahrovyrobnyky-mozhut-otrymaty-dopomohu-cherez-dar
Knowledge4policy (2021) Nutritional value of whole grains. Dataset. Knowledge for policy. Supporting policy with scientific evidence. https://knowledge4policy.ec.europa.eu/health-promotion-knowledge-gateway/whole-grain-nutritional-value-whole-2_en
Kovda, V.A. and Rozanov, B.G. 1988. Soil types, their georraphy and use, vol. 2. In: *Pedology*. Moscow: Vysshaya Shkola (in Russian).
Kramaryov, S. M., Kramaryov, O. S., Khristenko, A. O., Tokmakova, L. M., Zhuchenko, S. I., Syrovatko, V. A., Tsyova Yu.A., Syrovatko, K. V. (2015). Comparative assessment of the content of mobile phosphorus in different genetic horizons of ordinary chernozem. *Bulletin of the Poltava State Agrarian Academy*, **1-2**, 29-31. (In Ukrainian) http://nbuv.gov.ua/UJRN/VPDAA_2015_1-2_7
Krause, H. M., Mäder, P., Fliessbach, A., Jarosch, K. A., Oberson, A., & Mayer, J. (2024). Organic cropping systems balance environmental impacts and agricultural production. *Scientific Reports*, **14**, 1-15.
Krupenikov, I. A., Boincean, B. P., Dent, D. (2011). *The black earth: ecological principles for sustainable agriculture on chernozem soils*. Dordrecht: Springer Science & Business Media.
Lam, S. K., Suter, H., Bai, M., Walker, C., Davies, R., Mosier, A. R., & Chen, D. (2018a). Using urease and nitrification inhibitors to decrease ammonia and nitrous oxide emissions and improve productivity in a subtropical pasture. *Science of the Total Environment*, **644**, 1531-1535. 10.1016/j.scitotenv.2018.07.092
Lam, S. K., Suter, H., Mosier, A. R., & Chen, D. (2018b). Using nitrification inhibitors to mitigate agricultural N_2O emission: a double-edged sword? *Global Change Biology*, **23**, 485–489. https://doi.org/10.1111/gcb.13338
Latifundist.com (2024). Soil degradation. How are agricultural companies overcoming the problem and what are the options for restoring soil health in the territories affected by military actions? Press article (January 8th, 2024) https://latifundist.com/reportazhy/171-degradatsiya-gruntiv-yak-dolayut-problemu-v-agrokompaniyah-ta-yaki-ye-varianti-vidnovlennya-zdorovya-gruntiv-na-teritoriyah-shcho-postrazhdali-vnaslidok-voyennih-dij
Lerman, Z. (2008). Agricultural recovery in the former Soviet Union: An overview of 15 years of land reform and farm restructuring. *Post-communist economies*, **20**, 391-412.
Li, S.T., Duan, Y., Guo, T.W., Zhang, P.L., He, P., Kaushik, M. (2018). Sunflower response to potassium fertilization and nutrient requirement estimation. *Journal of integrative agriculture*, **17**, 2802-2812.
Li, Y. *et al.* (2023). Dissecting the contributions of organic nitrogen aerosols to global atmospheric nitrogen deposition and implications for ecosystems. *National Science Review*, **10**, nwad244.
Liu, M. *et al.* (2024). Effects of leguminous green manure–crop rotation on soil enzyme activity and stoichiometry. *Journal of Plant Ecology*, *17*(6), rtae065.
Lorimor, J., W. Powers and A. Sutton. 2004. Manure Characteristics. Midwest Plan Service-18, Section 1, MWPS-18 (1993), Section 1, Tables 7,10. https://www.canr.msu.edu/uploads/files/manurecharacteristicsmwps-18_1.pdf
Ludemann, C. I., Gruere, A., Heffer, P., & Dobermann, A. (2022). Global data on fertilizer use by crop and by country. *Scientific Data*, **9**, 1-8.
Ludemann, C. I., Hijbeek, R., van Loon, M., Murrell, S. T., Dobermann, A., van Ittersum, M. K. (2023). Global data on crop nutrient concentration and harvest indices. Dryad [data set]. https://doi.org/10.5061/dryad.n2z34tn0x

Ludemann, C. I. *et al.* (2024). A global FAOSTAT reference database of cropland nutrient budgets and nutrient use efficiency (1961–2020): nitrogen, phosphorus and potassium. *Earth System Science Data*, **16**, 525-541.
Ma, H., Jia, X., Yang, J., Liu, J., Shangguan, Z., & Yan, W. (2023). Inhibitors mitigate N2O emissions more effectively than biochar: A global perspective. *Science of The Total Environment*, **859**, 160416.
Maltais-Landry, G., Scow, K., & Brennan, E. (2014). Soil phosphorus mobilization in the rhizosphere of cover crops has little effect on phosphorus cycling in California agricultural soils. *Soil Biology and Biochemistry*, **78,** 255-262.
Mamonova, N., Wengle, S., Dankevych, V. (2023). Queen of the fields in wartime: what can Ukrainian corn tell us about the resilience of the global food system? *The Journal of Peasant Studies*, **50**, 2513-2538. https://doi.org/10.1080/03066150.2023.2255568
Makosh (2024). Fertilizer sales. Makosh Group https://makosh-group.com.ua/produktsja/
Matse, D. T., Krol, D. J., Richards, K. G., Danaher, M., Cummins, E., Wang, X., & Forrestal, P. J. (2024). Field efficacy of urease inhibitors for mitigation of ammonia emissions in agricultural field settings: a systematic review. *Frontiers in Environmental Science*, *12*, 1462098.
McKenzie, R.M., Özel, M.Z., Cape, J.N., Drewer, J. *et al.* (2016). The import and export of organic nitrogen species at a Scottish ombrotrophic peatland. *Biogeosciences*, **13**, 2353-2365.
Medinets, S. (2014). The Black Sea nitrogen budget revision in accordance with recent atmospheric deposition study. *Turkish Journal of Fisheries and Aquatic Sciences*, **14**, 981-992.
Medinets, S. and Medinets, V. (2012). Investigations of atmospheric wet and dry nutrient deposition to marine surface in western part of the Black Sea. *Turkish journal of fisheries and aquatic sciences*, **12**, 497-505.
Medinets, S. & Sutton, M.A. (2025). Nitrogen benchmarking for cropping systems. *Nature Food*, **6**, 645-646. https://doi.org/10.1038/s43016-025-01201-3
Medinets, S., Gasche, R., Skiba, U., Medinets, V., Butterbach-Bahl, K. (2016). The impact of management and climate on soil nitric oxide fluxes from arable land in the Southern Ukraine. *Atmospheric Environment*, **137**, 113-126.
Medinets, S., Kovalova, N., Medinets, V. *et al.* (2020). Assessment of riverine loads of nitrogen and phosphorus to the Dniester Estuary and the Black Sea over 2010-2019. In *Monitoring of Geological Processes and Ecological Condition of the Environment.* European Association of Geoscientists & Engineers. https://doi.org/10.3997/2214-4609.202056029
Medinets, S., Kovalova, N., Mileva, A. *et al.* (2021). Soil fluxes of reactive Nitrogen and GHG from arable land in south-western Ukraine: EU projects intervention. Chapter 20. In: *Regenerative Agriculture*, (ed. D. Dent and B. Boincean). Cham: Springer Nature, pp. 225-235.
Medinets, S., Medinets, V., Mileva, A., Khitrych, V., Gazyetov, Derevencha, V., Ye., Rozhenko, M., Bilivska, V., Petskovych, N., Vengrynovych, O., Turchyn, S., Fedoriak, M. (2024). Atmospheric deposition of nitrogen and sulphur in the Dniester catchment: the impact of war at a glance. *International Journal of Environmental Studies*, **81**, *250-259.*
Mi, X., He, G., Qiu, W., Zhang, Q., Yin, Y., Liu, J., Shi, M., Wang, Z., Cui, Z. (2025). Data-driven nitrogen management benchmarks support China's wheat self-sufficiency by 2030. *Nature Food.* https://doi.org/10.1038/s43016-025-01197-w.
Mishchenko, N. and Gumeniuk, K. (2006). *Agro-Ecological Assessment for the Transition of the Agricultural Sector in Ukraine. Part I: Socio-Economic Aspects.* IIASA Interim Report. IIASA, Laxenburg, Austria: IR-06-052. https://pure.iiasa.ac.at/id/eprint/8046/
Miyazaki, Y., Kawamura, K., Sawano, M. (2010). Size distributions of organic nitrogen and carbon in remote marine aerosols: Evidence of marine biological origin based on their isotopic ratios. *Geophysical Research Letters*, **37**, L06803.
NAP (2022). *Draft Plan for the Recovery of Ukraine. Materials of the Working Group "New Agrarian Policy" (July 2022)*. National Council for the Recovery of Ukraine from the Consequences of the War. https://www.kmu.gov.ua/storage/app/sites/1/recoveryrada/ua/new-agrarian-policy.pdf
NASU (2022). *Farmers in the reconstruction of Ukraine: a dialogue between scientists, civil society and the authorities (December 3, 2022).* Online article on the results and discussions from the *People's Forum Peasant and Farm Households During the War and in the Period of Post-War Reconstruction of Ukraine: Political Dialogue* (November 15-16, 2022) (in Ukrainian) https://old.nas.gov.ua/UA//Messages/Pages/View.aspx?MessageID=9686
Nosko B.S. (1987). Changes in the humus state of typical chernozem under the influence of fertilization. *Pochvovedenie*, **5**, 26-32 (in Russian).
Nosko B.S. and Chesnyak G.Ya. (1988). Extended reproduction of soil fertility in intensive agriculture in the conditions of Ukraine. *Zemledelie*, **1**, 27-28 (in Russian).
Nosko B.S., Batsula A.A. and Chesnyak G.Ya. (1992). The humus condition of the soils of Ukraine and ways of its regulation. *Pochvovedenie*, **10**, 33-39 (in Russian).

NutritionalValue (2024) Nutritional Values For Common Foods And Products. https://www.nutritionvalue.org/Corn_grain%2C_white_nutritional_value.html
Orlov, D.S. (1990) *Humic Acids of Soils and the General Theory of Humification*. Moscow: Moscow State University Publishing, 180 pp. (in Russian).
Pang, J., Ryan, M. H., Lambers, H., & Siddique, K. H. (2018). Phosphorus acquisition and utilisation in crop legumes under global change. *Current Opinion in Plant Biology, 45*, 248-254.
Pisarenko V.M. and Pisarenko P.B. (2022). Organic fertilizers to protect soil fertility (Ed. V.M. Pisarenko). Public union "Poltava Society of Agriculture", Poltava, 156 p. (in Ukrainian). https://www.pdau.edu.ua/sites/default/files/academicdepartment/kafedra-zahyst-roslyn/organichnidobryvaknyga2022.pdf
Pinchuk, V., Symochko, L., Palapa, N., Ustymenko, O., Kichigina, O., Demyanyuk, O. (2021). Agroecological soil status in agroecosystems with monoculture. *International Journal of Ecosystems and Ecology Sciences,* **11** (1), 1-12 (in Ukrainian). https://doi.org/10.31407/ijees11.101
Plaza-Bonilla, D., Nolot, J. M., Raffaillac, D., & Justes, E. (2017). Innovative cropping systems to reduce N inputs and maintain wheat yields by inserting grain legumes and cover crops in southwestern France. *European journal of agronomy*, *82*, 331-341.
Ponomareva, V.V. and Plotnikova, T.A. (1980). *Humus and Soil Formation.* Leningrad: Nauka, 319 pp. (in Russian).
Recio, J., Montoya, M., Álvarez, J. M., & Vallejo, A. (2020). Inhibitor-coated enhanced-efficiency N fertilizers for mitigating NOx and N2O emissions in a high-temperature irrigated agroecosystem. *Agricultural and Forest Meteorology*, *292*, 108110.
Ren, B., Huang, Z., Liu, P., Zhao, B., & Zhang, J. (2023). Urea ammonium nitrate solution combined with urease and nitrification inhibitors jointly mitigate NH3 and N2O emissions and improves nitrogen efficiency of summer maize under fertigation. *Field Crops Research*, *296*, 108909.
Ren, F., Sun, N., Misselbrook, T., Wu, L., Xu, M., Zhang, F., & Xu, W. (2022a). Responses of crop productivity and reactive nitrogen losses to the application of animal manure to China's main crops: A meta-analysis. *Science of The Total Environment*, *850*, 158064. 10.1016/j.scitotenv.2022.158064
Ren, K. *et al.* (2022b). Optimizing nitrogen fertilizer use for more grain and less pollution. *Journal of Cleaner Production*, *360*, 132180. https://doi.org/10.1016/j.jclepro.2022.132180
Romanova S. (2023). How the condition of the soil changes over the years, how it was and what we have come to. Recordings of the 2023 conference "National Challenge: Soil Degradation or Restoration of Their Fertility?". BTU Soil Health (in Ukrainian). https://soil.btu-center.com/records2023 https://www.youtube.com/watch?v=sHZiBQfqXJ8
Romanova, S.A., Zapasny, V.S., Hryshchenko, O.M. (2022). Current state of the soil cover of Ukraine. Collection of scientific works of Soil Protection. *Materials of the international conference "Soil conservation imperatives. Challenges today" (Kyiv, December 5, 2022)*, 7-12 (in Ukrainian). https://www.iogu.gov.ua/literature/soil/9-Спеціальний%20випуск%20(2018).pdf
Russell, E.W. (1973). *Soil Conditions and Plant Growth*. 10th Edition. Longman, London, 849 pp.
Sapkota, T. B. *et al.* (2021). Crop nutrient management using Nutrient Expert improves yield, increases farmers' income and reduces greenhouse gas emissions. *Scientific reports*, **11**, 1564. https://doi.org/10.1038/s41598-020-79883-x
Shapiro, C., Johnson, L. J., Schmidt, A., & Koelsch, R. (2021). Determining crop available nutrients from manure (revision: June 2021). University of Nebraska-Lincoln Extension. https://extensionpubs.unl.edu/publication/9000017123651/determining-crop-available-nutrients-from-manure-g1335/
Sharma, N., Kumar, R., Singh, A. P., Sharma, R., Sharma, P., MeCarty, J. S., & Farooq, F. (2025). Legumes in Cropping System for Soil Ecosystem Improvement: A Review. *Legume Research-An International Journal*, *1*, 9.
Seraya T.M., Bogatyreva E.N., Kidrun T.M., Zhabrovskaya N.Yu. (2024) Organic fertilizers: types and chemical composition. *Our agriculture,* **7**(8) (in Russian).
Serra, J. *et al.* (2024). Assessing nitrate groundwater hotspots in Europe reveals an inadequate designation of Nitrate Vulnerable Zones. *Chemosphere*, *355*, 141830.
Serra, J., Medinets, S., Lassaletta, L., Zhang, X., Boincean B., Aguilera, E. (2025). Missing inputs and outputs. In: Lassaletta, L., Sanz-Cobena, A. (Eds.), *Guidance document on nitrogen use efficiency methodology across multiple scales*. INMS Guidance Document Series. International Nitrogen Management System, UK Centre for Ecology & Hydrology, Edinburgh, UK.
Soares, J. R. *et al.* (2023). Mitigation of nitrous oxide emissions in grazing systems through nitrification inhibitors: a meta-analysis. *Nutrient cycling in agroecosystems*, **125**, 359-377. https://doi.org/10.1007/s10705-022-10256-8
Spears, B. M., Harpham, Q., Brown, E., Barnett, C. L., Barwell, L., Collell, M. R. *et al.* (2024). A rapid environmental risk assessment of the Kakhovka Dam breach during the Ukraine conflict. *Nature ecology & evolution*, **8**, 834-8361.

SSSU (2025). State Statistics Service of Ukraine (UkrStat). http://ukrstat.gov.ua/
Stutter, M. I., Shand, C. A., George, T. S., Blackwell, M. S., Bol, R., MacKay, R. L. *et al.* (2012). Recovering phosphorus from soil: a root solution? *Environmental Science & Technology*, **46**, 1977−1978.
Superagronom.com (2017). We "fertilize" the land: organic fertilizers for soil reproduction and increased fertility (February 14, 2017). https://superagronom.com/articles/40-zadobryuyemo-zemlyu-organichni-dobriva-dlya-vidtvorennya-gruntiv-i-pidvischennya-rodyuchosti
Superagronom (2023). The quality of the soils of Ukraine and their suitability for agricultural production — the results of an agrochemical survey. Press article (December 20th, 2023). https://superagronom.com/articles/699-yakist-gruntiv-ukrayini-ta-yih-pridatnist-dlya-silgospvirobnitstva--rezultati-agrohimichnogo-obstejennya
Superagronom.com (2024). Ukrainian fertilizer producers increased prices (June 21, 2024). https://superagronom.com/news/19123-ukrayinski-virobniki-azotnih-dobriv-pidvischili-tsini
Svitlychnyi, O.O. *et al.* (2022) Establishing the Scale and Consequences of Chernozem Degradation in Ukraine under Contemporary Climate Change and Agricultural Use. Final Report. Odesa: Odesa National University. State registration number: 0120U102180.
Teng L., Massey R., McCann L., Canter T., Omura S., Willett C., Roach A., Key N., Dodson L. (2023). Increasing the Value of Animal Manure for Farmers (March 2023), AP-109, U.S. Department of Agriculture, Economic Research Service. https://www.ers.usda.gov/webdocs/publications/106089/ap-109.pdf?v=2879.4
Thapa, R., Chatterjee, A., Awale, R., McGranahan, D. A., & Daigh, A. (2016a). Effect of enhanced efficiency fertilizers on nitrous oxide emissions and crop yields: A meta-analysis. *Soil Science Society of America Journal*, *80*(5), 1121-1134.
Tipping, E. *et al.* (2014). Atmospheric deposition of phosphorus to land and freshwater. *Environmental Science: Processes & Impacts*, **16**(7), 1608-1617.
tni.org (2023). *Ukrainian agriculture in wartime: Resilience, reforms, and markets.* Press article (November 9, 2023). https://www.tni.org/en/article/ukrainian-agriculture-in-wartime
Ukraine (2024). *Law of Ukraine on Amendments Regarding Military Service, Mobilization, and Military Records* (May 8, 2024). https://zakon.rada.gov.ua/laws/show/3633-20#Note
USDA (2020). FoodData Central. US Department of Agriculture. Agricultural Research Centre. https://fdc.nal.usda.gov/fdc-app.html#/food-details/790085/nutrients
USDA (2022). Ukraine Agricultural Production and Trade (July 2022). United States Department of Agriculture. Foreign Agricultural Service. https://fas.usda.gov/sites/default/files/2022-07/Ukraine-Factsheet-July2022.pdf
USDA (2024). Grains: World Markets and Trade (February 8, 2024). Production, Supply and Distribution. Reports and Data. United States Department of Agriculture. Foreign Agricultural Service. https://apps.fas.usda.gov/psdonline/app/index.html#/app/downloads
von Lampe, M. *et al.* (2014-11-28), "Fertiliser and Biofuel Policies in the Global Agricultural Supply Chain: Implications for Agricultural Markets and Farm Incomes", *OECD Food, Agriculture and Fisheries Papers*, No. 69, OECD Publishing, Paris. https://www.oecd.org/content/dam/oecd/en/publications/reports/2014/11/fertiliser-and-biofuel-policies-in-the-global-agricultural-supply-chain_g17a257d/5jxsr7tt3qf4-en.pdf
World Bank (2023a). *Ukraine Rapid Damage and Needs Assessment (February 2022 - February 2023).* https://documents1.worldbank.org/curated/en/099184503212328877/pdf/P1801740d1177f03c0ab180057556615497.pdf
World Bank (2023b). *Ukraine: Supporting Small Farms Amidst War.* Press article (October 30, 2023). https://www.worldbank.org/en/news/feature/2023/10/30/ukraine-supporting-small-farms-amidst-war
Yang, M., & Yang, H. (2021). *Utilization of soil residual phosphorus and internal reuse of phosphorus by crops. PeerJ 9: e11704*.
Yang, X. *et al.* (2024). Diversifying crop rotation increases food production, reduces net greenhouse gas emissions and improves soil health. *Nature Communications*, *15*(1), 198.
Yatsuk, I. P. & Baliuk S.A. (2019). Methodology of agrochemical certification of agricultural lands: a regulatory document (2nd ed.), Kyiv, 108 p. (in Ukrainian). https://www.iogu.gov.ua/literature/instructions/1.pdf
Yu, H., Wang, F., Shao, M., Huang, L., Xie, Y., Xu, Y., & Kong, L. (2021). Effects of rotations with legume on soil functional microbial communities involved in phosphorus transformation. *Frontiers in microbiology*, *12*, 661100.
Zander, P. *et al.* (2016). Grain legume decline and potential recovery in European agriculture: a review. *Agronomy for sustainable development*, *36*, 1-20. https://doi.org/10.1007/s13593-016-0365-y
