# Supplementary material for: Nutrient asymmetry challenges the sustainability of Ukrainian agriculture
Source: Commun Earth Environ. 2025 Nov 4;6(1):845. doi: 10.1038/s43247-025-02826-9 (PMC12588375; doi:10.1038/s43247-025-02826-9)
Supplement: Supplementary file 3 — Reporting Summary [file 43247_2025_2826_MOESM3_ESM.pdf]

nature portfolio

Corresponding author(s): Sergiy Medinets, Mark Sutton

Last updated by author(s): Aug 18, 2025

Reporting Summary

Nature Portfolio wishes to improve the reproducibility of the work that we publish. This form provides structure for consistency and transparency in reporting. For further information on Nature Portfolio policies, see our Editorial Policies and the Editorial Policy Checklist.

Statistics

For all statistical analyses, confirm that the following items are present in the figure legend, table legend, main text, or Methods section.

n/a	Confirmed	
☐	☒	The exact sample size (*n*) for each experimental group/condition, given as a discrete number and unit of measurement
☒	☐	A statement on whether measurements were taken from distinct samples or whether the same sample was measured repeatedly
☐	☒	The statistical test(s) used AND whether they are one- or two-sided<br>*Only common tests should be described solely by name; describe more complex techniques in the Methods section.*
☐	☒	A description of all covariates tested
☐	☒	A description of any assumptions or corrections, such as tests of normality and adjustment for multiple comparisons
☐	☒	A full description of the statistical parameters including central tendency (e.g. means) or other basic estimates (e.g. regression coefficient) AND variation (e.g. standard deviation) or associated estimates of uncertainty (e.g. confidence intervals)
☒	☐	For null hypothesis testing, the test statistic (e.g. *F*, *t*, *r*) with confidence intervals, effect sizes, degrees of freedom and *P* value noted<br>*Give P values as exact values whenever suitable.*
☒	☐	For Bayesian analysis, information on the choice of priors and Markov chain Monte Carlo settings
☒	☐	For hierarchical and complex designs, identification of the appropriate level for tests and full reporting of outcomes
☒	☐	Estimates of effect sizes (e.g. Cohen's *d*, Pearson's *r*), indicating how they were calculated

Our web collection on statistics for biologists contains articles on many of the points above.

Software and code

Policy information about availability of computer code

Data collection	No software were used to collect data.
Data analysis	We developed custom code to visualize and partially analyze data using R Statistical Software v4.4.2.

For manuscripts utilizing custom algorithms or software that are central to the research but not yet described in published literature, software must be made available to editors and reviewers. We strongly encourage code deposition in a community repository (e.g. GitHub). See the Nature Portfolio guidelines for submitting code & software for further information.

Data

Policy information about availability of data

All manuscripts must include a data availability statement. This statement should provide the following information, where applicable:

- Accession codes, unique identifiers, or web links for publicly available datasets
- A description of any restrictions on data availability
- For clinical datasets or third party data, please ensure that the statement adheres to our policy

Agricultural statistical data used in this study are freely available from the State Statistics Service of Ukraine (https://ukrstat.gov.ua/). These data are also compiled in XLS files and can be provided upon request. The crop removal coefficients are available from the global dataset by Ludemann et al., 2023 (https://doi.org/10.5061/dryad.n2z34tn0x). Additionally, we used global datasets on fertilizer production, fertilizer use, and staple crop exports, freely available from FAO

and Ludemann et al., 2022 (https://doi.org/10.5061/dryad.2rbnzs7qh). Our own estimates of the weighted content of N, P, and K in 'undefined' manure reported by Ukrainian statistics are presented in the Supplementary Information.

Human research participants

Policy information about studies involving human research participants and Sex and Gender in Research.

Reporting on sex and gender	N/A
Population characteristics	N/A
Recruitment	N/A
Ethics oversight	N/A

Note that full information on the approval of the study protocol must also be provided in the manuscript.

Field-specific reporting

Please select the one below that is the best fit for your research. If you are not sure, read the appropriate sections before making your selection.

☐ Life sciences ☐ Behavioural & social sciences ☒ Ecological, evolutionary & environmental sciences

For a reference copy of the document with all sections, see nature.com/documents/nr-reporting-summary-flat.pdf

Ecological, evolutionary & environmental sciences study design

All studies must disclose on these points even when the disclosure is negative.

Study description	We analysed publicly-available Ukraine's national statistical data to estimate agricultural balances for NPK and fertilizer-derived nutrient use efficiencies in production of wheat, maize and sunflower. We used annual statistical data (yield, synthetic and organic fertilizer use per crop) reported by SSSU14 to calculate average synthetic and organic NPK fertilizer inputs as well as yield per hectare of utilized agricultural area (UAA). Where county-level data were available (since 2007 for sunflower and maize, and since 2009 for wheat), we used these to calculate UAA-weighted annual magnitudes. We included nutrient inputs via organic (including manure) and synthetic fertilizers only, thereby neglecting inputs via atmospheric deposition (see below), irrigation, soil weathering and mineralization. Thus, annual agricultural balance of each crop (wheat, sunflower, maize) for each nutrient (NPK) was calculated as the difference between a total fertilizer input, that is, the sum of synthetic and organic fertilizer, applied as annual mass of NPK per ha of UAA, minus the crop yield, expressed as the corresponding nutrient mass per ha of UAA. Positive nutrient balances indicate nutrient inputs via fertilizers, while negative balances reflect a deficit. We estimated total fertilizer NPK use efficiencies (NUE, PUE, KUE) for each cropping system as the ratio of nutrient in harvested yield to nutrient inputs via organic and synthetic fertilizers applied. We highlight general trends for wheat, maize and sunflower crops, while recognizing that considering crop rotations may influence results at the site level.
Research sample	We used annual data reported by Ukraine's State Statistics Service at two spatial scales in this study: the country scale (i.e., the entire Ukraine) and the county scale (i.e., an administrative unit). County data, available from 2007/2009, were used in a weighted manner to accurately represent both country and regional data.
Sampling strategy	We used annual data reported by Ukraine's State Statistics Service at two spatial scales in this study: the country scale (i.e., the entire Ukraine) and the county scale (i.e., an administrative unit). County data, available from 2007/2009, were used in a weighted manner to accurately represent both country and regional data.
Data collection	All the data collected were publicly available. Agricultural statistical data were downloaded from the State Statistics Service of Ukraine (https://ukrstat.gov.ua/). The compilation of crop removal coefficients was obtained from Ludemann et al., 2023 (https://doi.org/10.5061/dryad.n2z34tn0x). Other global datasets, inter alia fertilizer production, fertilizer use, and staple crop exports were downloaded from the corresponding sources, including FAO and Ludemann et al., 2022 (https://doi.org/10.5061/dryad.2rbnzs7qh).
Timing and spatial scale	We trace nutrient management during Ukraine's transition from the Soviet era (from 1980) through the challenges of independence (since 1991), culminating in the pre-war period of high agricultural productivity (2019-2021), and extending up to two years of war (2022-2023). Nationwide (country) and county spatial scales were used for data collection, while country and regional scales were used for data analysis.
Data exclusions	No data were excluded, except data for 2023 for Chernihivska county, as it reported an unexplained increase in fertilizer application: 1.6-fold (155 kg N ha-1) for sunflower and 1.7-fold (245 kg N ha-1) for maize in the 2nd year of the large-scale war, compared to 2021, the pre-war year, which was most likely an error. This is explained in the Supplementary Information.
Reproducibility	Due to data are either open access or available upon request, anyone can reproduce the results of our study
Randomization	N/A

Blinding	N/A

Did the study involve field work? ☐ Yes ☒ No

Reporting for specific materials, systems and methods

We require information from authors about some types of materials, experimental systems and methods used in many studies. Here, indicate whether each material, system or method listed is relevant to your study. If you are not sure if a list item applies to your research, read the appropriate section before selecting a response.

Materials & experimental systems

n/a	Involved in the study
☒	☐ Antibodies
☒	☐ Eukaryotic cell lines
☒	☐ Palaeontology and archaeology
☒	☐ Animals and other organisms
☒	☐ Clinical data
☒	☐ Dual use research of concern

Methods

n/a	Involved in the study
☒	☐ ChIP-seq
☒	☐ Flow cytometry
☒	☐ MRI-based neuroimaging
